# S3RL: Enhancing Spatial Single‐Cell Transcriptomics With Separable Representation Learning

**DOI:** 10.1002/advs.202516178

**Published:** 2026-01-20

**Authors:** Laiyi Fu, Penglei Wang, Gaoyuan Xu, Jitao Lu, Qinke Peng, Danyang Wu, Hequan Sun

**Affiliations:** ^1^ School of Automation Science and Engineering Xi'an Jiaotong University Xi'an China; ^2^ Research Institute Xi'an Jiaotong University Zhejiang Zhejiang China; ^3^ Sichuan Digital Economy Industry Development Research Institute Xi'an Jiaotong University Sichuan China; ^4^ School of Software Engineering South China University of Technology Guangzhou Guangdong China; ^5^ College of Information Engineering Northwest A&F University Xianyang Shannxi China

**Keywords:** cell–cell communication, graph neural networks, hyperspherical prototype learning, single‐cell RNA‐seq, spatial transcriptomics

## Abstract

Spatial transcriptomics enables in situ mapping of gene expression, offering insights into tissue organization and cell–cell interactions. However, its utility is limited by data sparsity and technical noise for decoding complex tissue microenvironments. Here, we introduce S3RL, a separable representation learning framework designed to enhance the fidelity of raw spatial transcriptomic data. By effectively denoising sparse measurements and amplifying biologically relevant signals, S3RL enables the recovery of fine‐grained spatial expression patterns and regulatory relationships that are otherwise lost. Applied across diverse human, mouse and plant tissues, S3RL not only achieved improved accuracy in spatial domain identification and multi‐slice alignment (up to 170% ARI improvement), but also uncovered previously unrecognized ligand–receptor signaling and spatial gene expression gradients that are critical for understanding immune‐tumor crosstalk and plant developmental trajectories. These results establish S3RL as a powerful tool for extracting latent biological programs from noisy spatial transcriptomic datasets, paving the way for deeper exploration of tissue biology and disease mechanisms.

## Graphical abstract

Separable Spatial Representation Learning (S3RL) is introduced to enhance the reconstruction of spatial transcriptomic landscapes by disentangling spatial structure and gene expression semantics. By integrating multimodal inputs with graph‐based representation learning and hyperspherical prototype modeling, S3RL enables high‐fidelity spatial domain recovery and improves downstream analyses, including expression enhancement, spatial alignment, and trajectory inference.

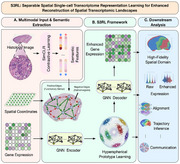

## 1 Introduction

The spatial arrangement of cells within tissues forms the foundation for organ function and developmental processes. From the layered architecture of the human cerebral cortex to the vascular bundles in plant seeds, cellular organization orchestrates cell fate decisions, intercellular signaling, and responses to environmental cues. Disruption of these spatial structures is often associated with pathological conditions such as cancer, neurodegeneration, and inflammatory diseases [1, 2, 3]. Identifying the spatial transcriptomic landscape is critifcal for advancing developmental biology and disease research [4, 5, 6].

The advent of spatial transcriptomics (ST) has enabled in situ mapping of the transcriptome landscape  [7]. Platforms such as 10X Visium [8], Slide‐seq [9], and Stereo‐seq [10] have provided unprecedented insights into spatially restricted gene expression programs. However, these technologies present inherent trade‐offs: spot‐based sequencing often captures signals from multiple cells, limiting the resolution of fine spatial features, while imaging‐based methods (e.g., SeqFISH [11]) achieve higher spatial resolution but can only profile a limited number of genes. Additionally, technical variability, data sparsity, and high dropout rates are particularly pronounced in complex tissues such as tumors and neurodegenerative regions [12].

Recent advances in ST technologies now enable the simultaneous generation of multimodal data, including gene expression profiles, spatial coordinates, and histological images (e.g., H&E‐stained sections) [8, 9, 10]. This integration of transcriptomics and tissue morphology opens new opportunities to analyze cellular organization and functional heterogeneity, particularly in spatially complex tissues [13].

To effectively integrate these multimodal datasets, numerous computational approaches have been proposed. For instance, Giotto [14] employs Hidden Markov Random Fields to capture spatial continuity, while SpaGCN [15] and CCST [16] use graph convolutional networks to incorporate spatial and gene expression features. stLearn [17] extracts morphological features from histological images and integrates them with transcriptomic data. Bayesian frameworks such as BayesSpace [18] improve spatial resolution via probabilistic inference. More recently, graph autoencoder‐based methods, including STAGATE [19], GraphST [20], SiGra [21], and SEDR [22] have advanced latent spatial representation learning. These approaches have collectively propelled spatial transcriptomics forward, enabling insights into cell fate specification, microenvironmental regulation, and cell‐cell communication networks [1, 2, 3]. To address the sparse signals and restricted gene coverage limitations of ST technologies, SpaIM disentangles shared content and modality‐specific styles to integrate rich gene expression from single‐cell RNA sequencing data with the spatial context of ST profiles, enabling accurate imputation of unmeasured or missing gene expressions [23].

Despite notable progress in spatial transcriptomics (ST) analysis, several critical challenges remain unresolved. Current methods often adopt a cell‐type‐free representation learning strategy, resulting in latent embeddings that fail to align with true biological cell types. This leads to overlapping clusters, blurred boundaries, and poor separability in latent space, limiting the interpretability and fidelity of downstream reconstruction. Additionally, histological images, while offering complementary spatial information, frequently exhibit structural homogeneity and subtle color variation. For instance, SiGra [21] is an image‐augmented graph transformer method that integrates multi‐channel immunohistochemistry images and gene expression data into a single‐cell spatial graph, enabling simultaneous spatial domain identification and enhancement of sparse, noisy spatial transcriptomics data. Although SiGra outperforms state‐of‐the‐art methods on both single‐cell and spot‐level spatial transcriptomics datasets, it simply flattens H&E‐stained images for neural network input and struggle to extract meaningful semantic features. Furthermore, graph construction approaches typically rely on physical proximity (e.g., Euclidean distance or k‐nearest neighbors) [15, 20, 22], overlooking functionally similar but spatially distant spots and occasionally connecting adjacent yet biologically distinct regions. These limitations, combined with the inherent sparsity and noise of ST data, obscure critical biological patterns such as spatially restricted ligand‐receptor interactions or developmental regulatory gradients.

To address these challenges, a unified computational framework is needed that integrates multimodal information, models cell‐type‐aware representations, and reconstructs high‐fidelity spatial transcriptomic landscapes, thereby enhancing both clustering accuracy and downstream biological analyses such as trajectory inference and cell‐cell communication mapping.

In this work, we present Separable Spatial Single‐cell Transcriptome Representation Learning (S3RL), a unified computational framework designed to address these unmet needs. By effectively extracting high‐level semantic features from histological images and combining them with gene expression similarity, S3RL preserves both local neighborhood relationships and long‐range functional connections within the cellular graph. This design overcomes the limitations of relying solely on physical proximity, allowing the model to reconstruct spatial structures that better reflect biological reality in complex tissue microenvironments. Importantly, S3RL introduces explicit constraints on cluster separability in latent space, ensuring that different cell types form clear boundaries in both spatial distribution and expression patterns, thereby markedly enhancing tissue structure resolution and reconstruction quality.

We systematically validated S3RL across diverse spatial transcriptomics datasets spanning multiple platforms (10X Visium, Nanostring CosMx, Stereo‐seq, STARmap‐PLUS, and Slide‐seqV2) and biological systems, including human, mouse, and plant tissues. S3RL consistently outperformed existing methods, achieving significant improvements in spatial clustering (e.g., nearly 170% mean ARI gain on Nanostring and 26% over baselines on DLPFC) and robust cross‐slice alignment. Notably, S3RL reconstructed high‐quality data that revealed biologically meaningful patterns–such as tumor‐immune interactions, localized ligand‐receptor networks, and functional tissue architecture–that were obscured in raw data. These results highlight S3RL's unique capacity to decode complex microenvironments and support comprehensive spatial omics analysis across platforms and species.

## 2 Results

### 2.1 Overview of the S3RL Method

S3RL was designed to address the key challenges in spatial transcriptomics by providing a unified framework capable of denoising, reconstructing, and analyzing complex spatial transcriptomic data. Rather than relying solely on physical proximity or raw expression profiles, S3RL integrates multimodal information–including gene expression, spatial coordinates, and histological image features–into a coherent representation of tissue architecture. This design enables the model to recover fine‐grained spatial organization even in noisy or sparse datasets. The S3RL workflow comprises three conceptual components. First, it extracts high‐level semantic features from histological images using contrastive learning, allowing the model to capture subtle morphological patterns that often correlate with biological function. Next, S3RL constructs a signed‐edge graph where positive edges reflect functional similarity between spots (even if spatially distant) and negative edges help separate neighboring spots with distinct transcriptional signatures or histological features. This graph serves as a biologically informed scaffold for learning spatial relationships beyond simple adjacency. Finally, the model performs representation learning and clustering on a hyperspherical manifold, where each spot's low‐dimensional embedding is mapped relative to cell‐type prototypes distributed evenly on the hypersphere. This separable representation ensures clear boundaries between distinct cellular domains, facilitating accurate spatial segmentation and improved recovery of biologically meaningful patterns. All datasets analyzed in this study are bona fide spatial transcriptomics datasets with explicit spatial coordinates, ensuring that the representations learned by S3RL are grounded in true spatially resolved gene expression measurements.

**FIGURE 1:**
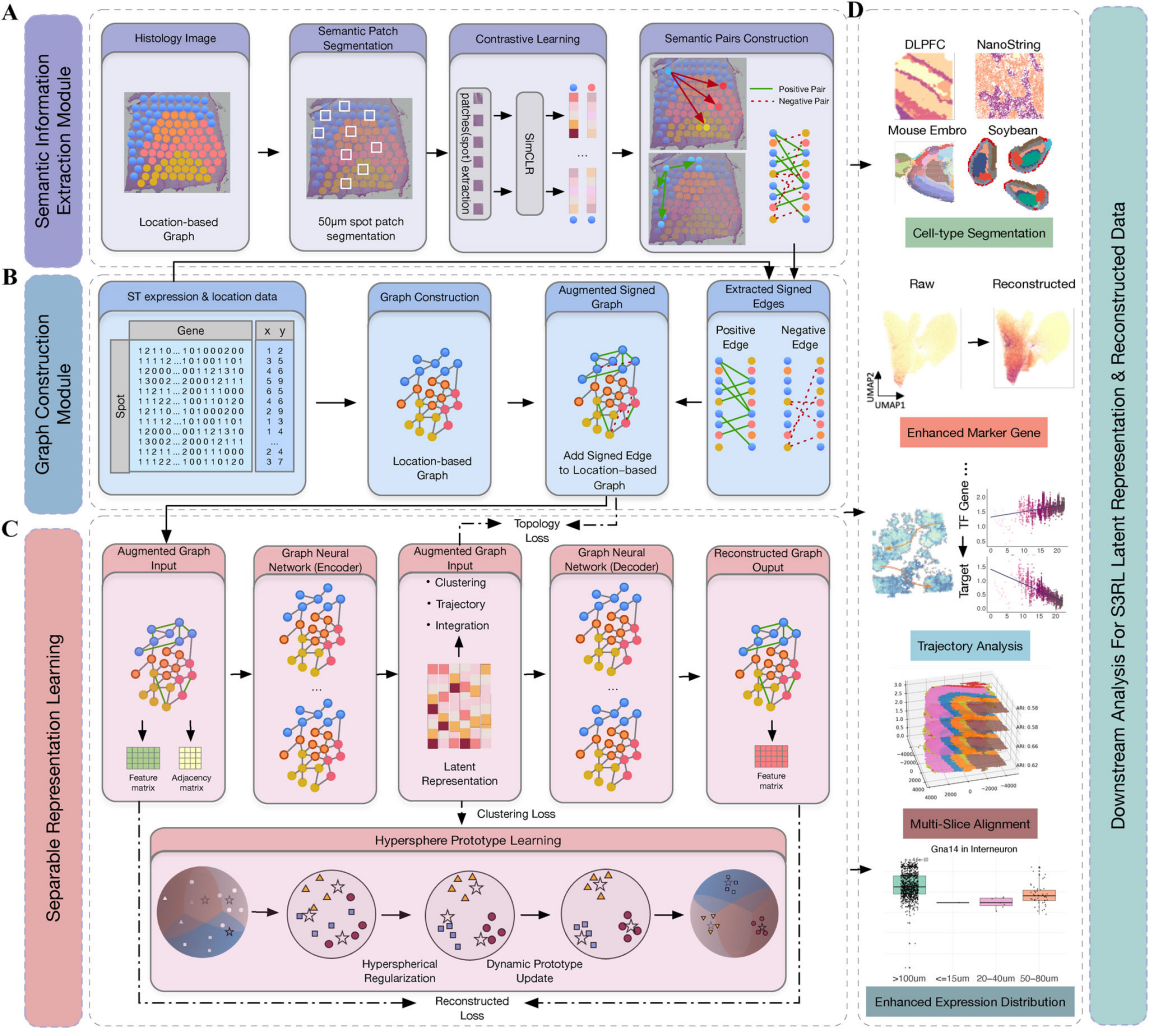
Overview of the S3RL framework for spatial transcriptomics analysis with four key modules. (A) Semantic Information Extraction Module: Histological images are segmented into spatially coherent regions using a 50μm spot patch segmentation approach. Contrastive learning (SimCLR) is then applied to extract high‐level semantic features, enabling the construction of positive/negative pairs based on similarity. (B) Signed Edge Extraction and Graph Construction Module: A spatial k‐nearest neighbor graph is first built using spot coordinates. This graph is augmented by integrating signed edges (positive for functionally similar spots, negative for dissimilar ones) derived from semantic and expression similarities, providing a biologically informed scaffold that captures both local and global spatial relationships. (C) Separable Representation Learning and Hyperspherical Prototype Clustering: A graph neural network (GNN) encoder‐decoder architecture is used to learn low‐dimensional representations that preserve the topological structure of signed edges. Representations are mapped to a unit hypersphere, where uniformly distributed prototypes define cluster centers. Hyperspherical regularization promotes cluster separability, and dynamic prototype updates adapt to dataset‐specific variations, ensuring accurate tissue segmentation and expression reconstruction. (D) Downstream Analysis of Enhanced Data: The reconstructed high‐fidelity data and learned embeddings enable a variety of downstream tasks, including multi‐slice alignment, pseudotime trajectory inference, ligand‐receptor interaction analysis, and differential gene expression mapping, facilitating biologically meaningful discoveries from complex spatial transcriptomic datasets.

Importantly, S3RL operates end‐to‐end: from raw multimodal inputs to refined cell type annotations and enhanced spatial gene expression profiles. Beyond improving clustering accuracy, the reconstructed data enables a wide range of downstream biological analyses, including cell‐type deconvolution, spatial trajectory inference, functional enrichment analyses (e.g., GSEA, GO), identification of spatially variable genes (SVGs), and construction of transcriptional regulatory networks. The overall workflow of S3RL framework is shown in (Figure 1A–D).

### 2.2 S3RL's Spatial Clustering of Human Dorsolateral Prefrontal Cortex 10x Visium Data Improves Layer Identification

As one of the most widely studied and benchmarked datasets in spatial transcriptomics, the human dorsolateral prefrontal cortex (DLPFC) dataset provides an ideal starting point for evaluating S3RL. With its well‐characterized cortical layer architecture, this dataset serves as a gold standard for assessing the ability of computational methods to resolve fine‐grained tissue structures. To this end, we first applied S3RL to the 10× Visium DLPFC dataset [24], which offers spatially resolved transcriptomic profiles across 12 slices. These slices capture four to six cortical layers and the white matter (WM) region, covering 33 538 genes from three neurotypical adult donors. Each donor contributed two adjacent 10 μm sections and a third slice 300 μm posterior, creating a rigorous benchmark for testing spatial alignment and tissue reconstruction performance.

**FIGURE 2:**
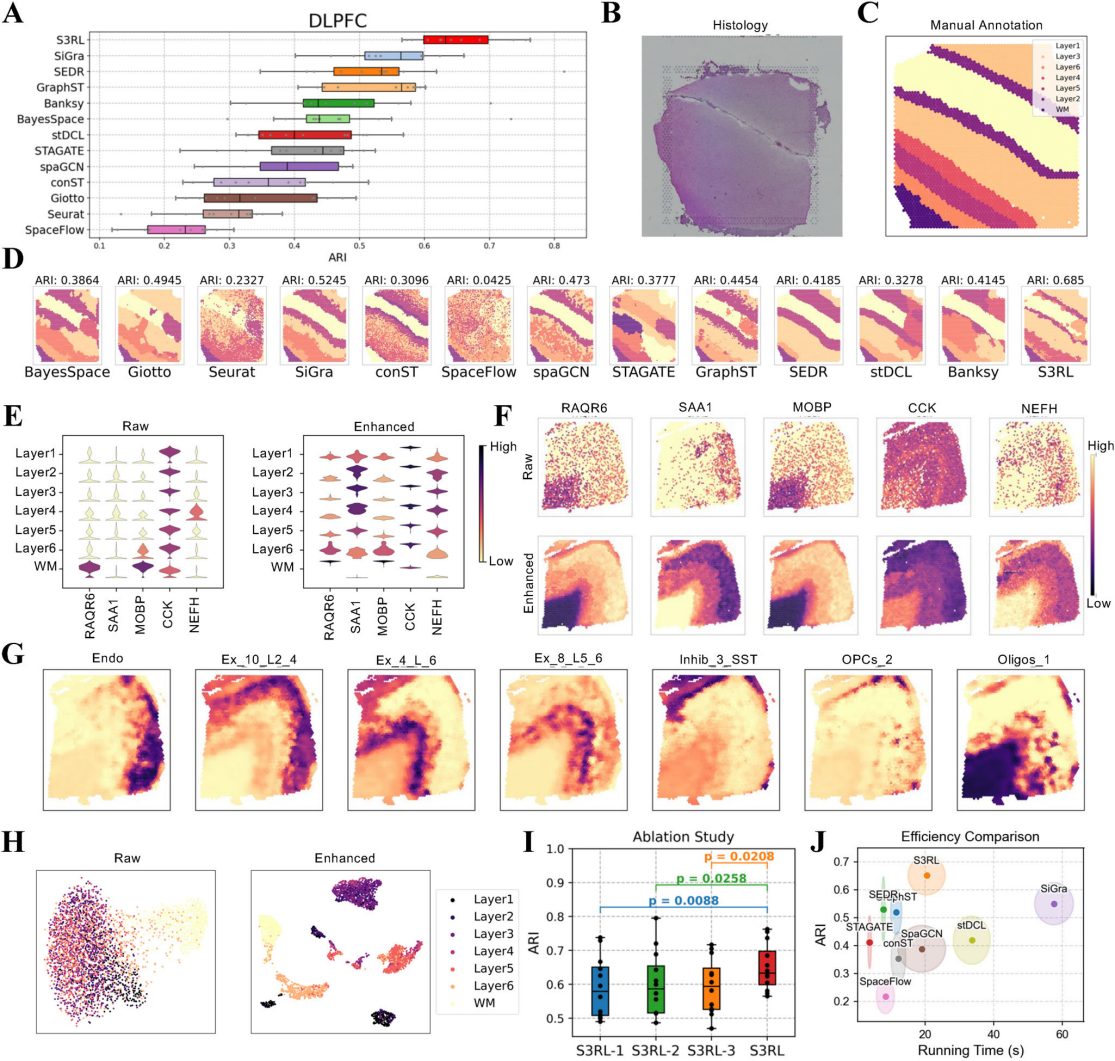
Spatial clustering and deconvolution analysis of human dorsolateral prefrontal cortex (DLPFC) using S3RL. (A) Comparison of Adjusted Rand Index (ARI) across different clustering methods on the DLPFC dataset. S3RL achieves the highest ARI of 0.57, outperforming other methods such as BayesSpace, Giotto, Seurat, SiGra, GraphST, and STAGATE. (B‐C) Histology image (B) and manually annotated spatial layers (C) used as ground truth for clustering evaluation. (D) Clustering results from various methods on slice 151673, where S3RL achieves an ARI of 0.57, demonstrating improved spatial organization and boundary accuracy compared to alternative approaches. (E) Violin plots comparing raw and enhanced expression distributions for marker genes across cortical layers, showing improved inter‐layer variability in enhanced data. (F) Spatial expression maps of representative marker genes (e.g., PD4R6, SAA1, MOBP, CCK, NEFH), highlighting sharper gene expression boundaries in the enhanced data. (G) Deconvolution analysis using scRNA‐seq and ST data on slice 151673, revealing spatial distributions of key cell types, including endothelial cells (Endo), excitatory neurons (Ex), inhibitory neurons (Inhib), oligodendrocyte precursor cells (OPCs), and oligodendrocytes (Oligos). (H) t‐SNE visualization of clustering results, demonstrating improved spatial structure in S3RL‐enhanced data. (I) Ablation study results, showing ARI scores for different variants of S3RL, where the full model configuration achieves the highest clustering performance. Statistical significance was determined using paired t‐tests between each variant and the full S3RL model, and different colors denote the specific variant being compared to the full model for visual clarity. (J) Efficiency comparison among different algorithms and the S3RL method. S3RL achieves the highest clustering accuracy (ARI ≈ 0.65) while maintaining a moderate running time of about 20 s.

Compared to state‐of‐the‐art methods including BayesSpace [18], Giotto [14], Seurat [25], SiGra [21], conST [26], SpaceFlow [27], spaGCN [15], STAGATE [19], SEDR [22], and GraphST [20], S3RL achieved the highest mean Adjusted Rand Index (ARI) of 0.65. This represents a 26.8% improvement over the average performance of all baselines and a 10.2% gain over the best‐performing competitor (SiGra), underscoring its superior ability to preserve layer boundaries and avoid over‐segmentation (Figure 2A–D, detailed clustering results are shown in Figure S1 and S2). In addition, to further verify that these improvements are not dataset‐specific fluctuations, we conducted paired t‐tests across all 12 DLPFC slice. As reported in Figure S3 and Table S1, S3RL consistently achieves significantly positive T‐values and low P‐values compared with all baseline methods, confirming that its performance gains are statistically robust rather than arising from random variation.

To illustrate S3RL's advantages in data reconstruction, we examined gene expression patterns across cortical layers in both raw and S3RL‐enhanced data (Figure 2E). Notably, the enhanced data exhibited sharper layer demarcations and restored subtle spatial gradients that were obscured in raw profiles. This refinement was particularly evident for marker genes such as *PAQR6*, *SAA1*, *CCX*, and *MOBP* [28], where S3RL‐enhanced data aligned more closely with known cortical structures (Figure 2F,H). Figure S4 further shows that embeddings from S3RL's latent space clustering were more compact and displayed clearer separation between cortical layers, indicating a more biologically faithful reconstruction of tissue architecture.

S3RL's reconstruction also improved downstream spatial analyses. Trajectory inference across the 12 DLPFC slices revealed biologically consistent inter‐layer transitions, visualized as connectivity graphs where node sizes reflect layer identities and edge thickness encodes interaction strengths (Figure S5). These graphs revealed robust connections between superficial layers (Layer 1) and deeper cortical regions (Layer 6, WM), a feature less discernible in raw data, highlighting S3RL's capacity to recover biologically meaningful spatial connectivity.

To further assess the biological interpretability of the multimodal features in S3RL, we examined the semantic information extracted from H&E images by applying Leiden clustering directly to the visual embeddings generated by the contrastive‐learning module. We found that these visual features alone can recover coarse but biologically coherent spatial patterns, such as approximate cortical laminar organization and gray–white matter separation in the DLPFC, as well as partial alignment with tumor‐ and stroma‐enriched regions in the Nanostring lung cancer dataset (Figure S6). However, the resulting boundaries remain diffuse and less accurate than those produced by the full S3RL framework, indicating that visual semantics capture meaningful histological cues but are insufficient on their own for precise spatial domain identification. These results confirm that the learned visual representations are interpretable rather than black‐box features, while highlighting the necessity of multimodal integration with transcriptomic information to achieve robust and accurate spatial reconstruction.

To dissect the mechanistic contributions of S3RL's components, we conducted a systematic ablation study comparing the full model against variants lacking visual semantic guidance (S3RL‐2), dynamic prototype updating (S3RL‐1), or both (S3RL‐3). Quantitative benchmarking (Figure 2I) confirms that the full S3RL model consistently outperforms all variants, with S3RL‐3 showing the lowest accuracy as expected. Qualitatively, our analysis reveals distinct roles for the two key modules: a) Visual Semantics as a Spatial Regularizer: Incorporating histological features effectively acts as a denoiser, preventing the “salt‐and‐pepper” fragmentation observed in variants relying solely on gene expression (S3RL‐2), thereby yielding smoother and more continuous tissue boundaries; b) Dynamic Prototypes for Latent Separability: The dynamic update mechanism adapts to expression heterogeneity, pulling intra‐class samples together to form compact, well‐separated clusters in the latent space, whereas static prototypes (S3RL‐1) result in blurred inter‐class boundaries. For a comprehensive visual comparison of spatial reconstructions and latent embeddings across all ablation variants, please refer to the Supplementary Information.

In addition to its superior clustering performance, S3RL demonstrates high computational efficiency. We benchmarked the training runtime and clustering accuracy (ARI) of S3RL against representative deep learning‐based methods on the DLPFC dataset. As shown in Figure 2J, S3RL occupies the optimal region of the performance‐runtime trade‐off, achieving the highest clustering accuracy (ARI ≈ 0.65) while maintaining a moderate runtime of approximately 20 s. Notably, S3RL is approximately three times faster than the transformer‐based method SiGra while yielding significantly better segmentation results, striking a favorable balance between computational cost and model performance.

### 2.3 Deconvolution Analysis on S3RL‐Reconstructed Spatial Data in DLPFC Slice 151673

Building on S3RL's improved spatial clustering and layer differentiation in the human dorsolateral prefrontal cortex (DLPFC) dataset, we next selected one representative slice (151673) for a more detailed analysis of cell type deconvolution performance on S3RL‐reconstructed data. This allowed us to assess how the enhanced spatial resolution and denoised expression profiles provided by S3RL impact downstream integration with single‐cell RNA sequencing (scRNA‐seq) data. We first applied S3RL to enhance the ST data, resulting in a spatial expression profile with clearer boundaries and improved expression fidelity. As for the reference scRNA‐seq data, we used a publicly available single‐nucleus RNA‐seq (snRNA‐seq) dataset from the human dorsolateral prefrontal cortex (BA9) [29], generated by the 10x Genomics Chromium platform (GEO accession: GSE144136). This dataset comprises 78,886 nuclei and expression profiles of 30 062 genes.

As illustrated in Figure S7, the deconvolution framework draws inspiration from GraphST but adopts a simplified contrastive learning‐based design. Specifically, we learn a projection matrix that maps scRNA‐seq expression into the spatial domain. Unlike GraphST, which utilizes an autoencoder‐reconstructed scRNA‐seq matrix, our method directly uses the raw input matrix, which helps preserve intrinsic expression variability across cells and avoids potential information loss introduced by latent‐space compression. The learned projection matrix transforms the scRNA‐seq expression into a reconstructed spatial gene expression matrix, which is then aligned to the S3RL‐enhanced ST matrix through contrastive learning. Positive and negative spot pairs are defined based on spatial proximity, enabling the model to maintain topological consistency while biologically estimating cell‐type compositions at each spatial location. This framework allows biologically meaningful deconvolution of spatial transcriptomics guided by single‐cell resolution information.

As shown in Figure 2G, our method effectively captures the spatial distributions of different cell types across the tissue, with distinct and well‐defined regions corresponding to different cell types. The heatmaps illustrate the proportions of key cell types, including various excitatory neurons (Ex), oligodendrocytes (Oligos) and endothelial cells (Endo). Compared to the raw ST data, the deconvolved results provide a much clearer depiction of the underlying cellular architecture, highlighting the enriched presence of specific cell types in certain layers of the DLPFC. What is more, the results also achieves high spatial resolution and reveals biologically meaningful patterns. For example, the excitatory neurons (Ex) are predominantly found in the outer layers, while oligodendrocytes are concentrated in the white matter regions (Figure 2G) [30]. The deconvolution results demonstrate the ability of our method to accurately map the cell‐type composition and provide a clearer representation of cellular heterogeneity in complex tissues like the DLPFC.

In addition, we present a qualitative comparison in Figure S8, where several representative reconstruction‐based methods (e.g., STAGATE, SEDR, GraphST, and SiGra) are evaluated using the same deconvolution framework as ours. Although it is challenging to perform precise quantitative evaluation, visual inspection reveals that S3RL consistently produces the clearest expression boundaries and the most coherent spatial patterns. This further highlights the superior spatial consistency and structural fidelity of the representations learned by S3RL, offering strong support for its effectiveness in downstream deconvolution analysis.

### 2.4 S3RL Improves Cell Type Clustering and Gene Expression Delineation on Nanostring Lung Slices

**FIGURE 3:**
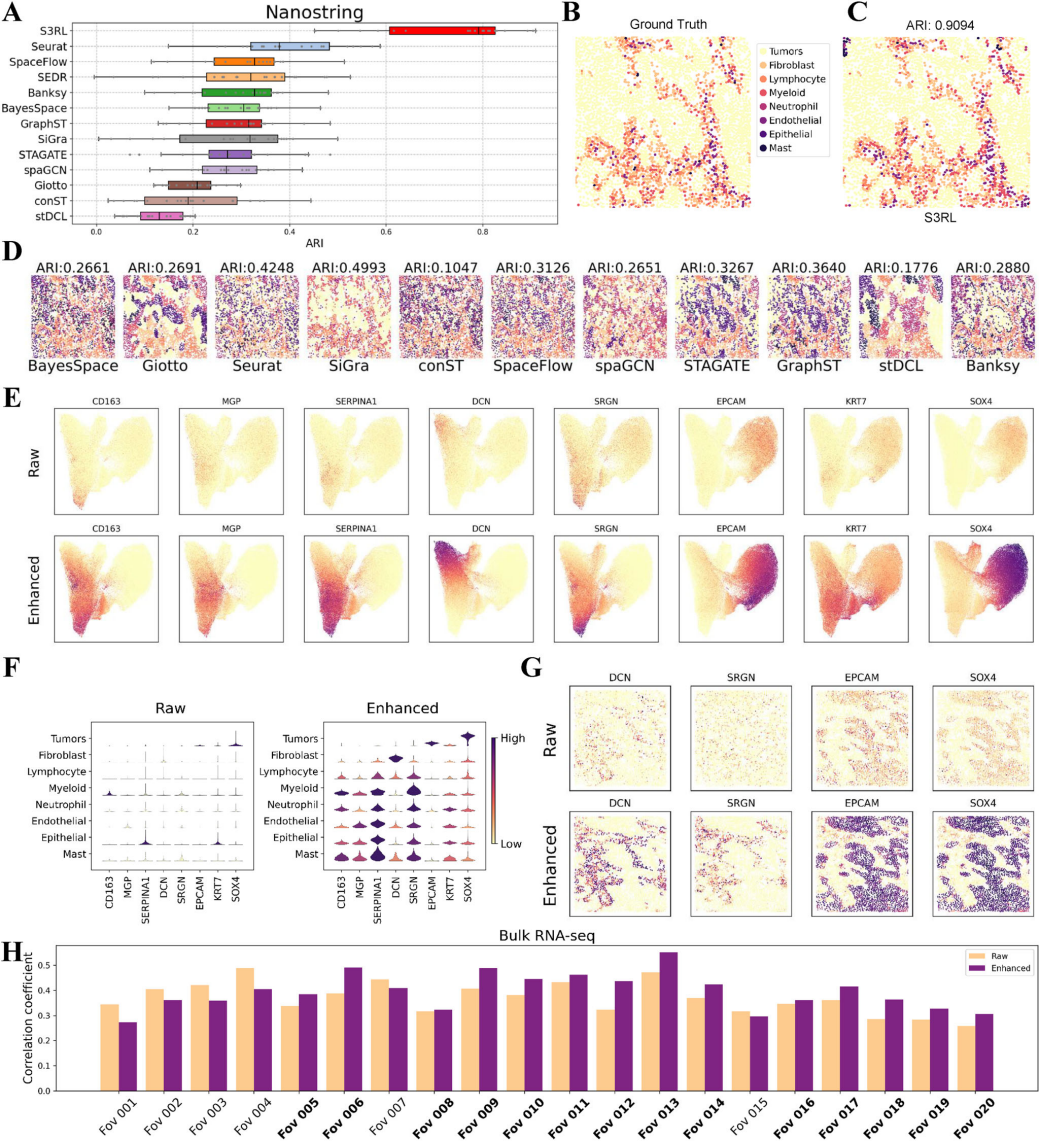
Nanostring Lung Slice Analysis and Correlation with TCGA Bulk RNA‐seq. (A) Comparison of clustering performance across multiple methods using Adjusted Rand Index (ARI) on the Nanostring dataset. Our model achieves the highest ARI of 0.9094, significantly outperforming state‐of‐the‐art methods such as Seurat, BayesSpace, SiGra, STAGATE, and GraphST. (B,C) Ground truth annotations (B) and the clustering result obtained using our model (C), demonstrating high spatial alignment with known cell types. (D) UMAP visualization of different clustering methods, showing clearer cluster separation in our enhanced data representation. (E) Spatial gene expression plots for selected markers (CD163, EPCAM, SOX4, etc.) before and after enhancement. The enhanced data exhibits sharper expression boundaries, revealing improved biological signal resolution. (F) Violin plots comparing raw and enhanced gene expression across different cell types, highlighting increased expression specificity in the enhanced data. (G) Spatial distribution of selected marker genes (EPCAM, DCN, SRGN, SOX4) across individual lung slices, where enhancement improves tissue‐specific gene localization. (H) Correlation analysis between TCGA bulk RNA‐seq data and Nanostring spatial transcriptomics (ST) data across 20 slices. Each bar represents the Pearson correlation coefficient between the bulk data and either raw (orange) or S3RL‐enhanced (purple) spatial data. Slices where the enhanced data outperforms the raw are highlighted in bold, indicating improved alignment with bulk gene expression profiles in 14 out of 20 slices.

To explore S3RL's performance in complex pathological tissues, we first analyzed the Nanostring lung cancer dataset, which consists of 20 lung tissue slices [31]. This dataset offers a challenging benchmark due to its high cell‐type heterogeneity and the presence of intricate tumor microenvironments. Using the latent space representations learned by our model, we performed unsupervised clustering and compared the results against several state‐of‐the‐art spatial transcriptomics analysis methods (Figure 3A, Figure S9 presents the clustering results for all 20 slices in the latent space and Figures S10 and S11 presents the clustering results over all the methods across all the slices). S3RL achieved a mean Adjusted Rand Index (ARI) of 0.7274 across all slices–substantially outperforming Seurat (0.3989), the best baseline method, with a relative improvement of 82.4%. Remarkably, S3RL's average performance exceeded the mean of all other methods by 170.7%, underlining its superior ability to delineate spatial domains and cell types even in highly heterogeneous tissues (Figure 3B‐D). This significant gain highlights S3RL's capacity to reconstruct cleaner, biologically coherent spatial representations from noisy raw data. We additionally assessed the statistical robustness of these improvements by performing paired t‐tests across all 20 Nanostring slices. As shown in Figure S3 and Table S1, S3RL achieves consistently positive T‐values and significantly low P‐values relative to all competing methods, demonstrating that its advantages in highly heterogeneous pathological tissues are statistically reliable rather than arising from random variation.

To investigate whether S3RL‐enhanced(reconstructed) data better preserves biologically meaningful structures at the single‐slice level, we conducted a comparison of raw and enhanced data on the Nanostring dataset using slice 014. Our model achieved the highest ARI of 0.9094, significantly outperforming other methods (Figure 3D). The UMAP visualization plot of gene expression across all 20 slices (Figure 3E) shows that the S3RL‐enhanced data exhibits a clearer separation of clusters compared to raw data, indicating improved resolution of cell‐type heterogeneity (Figure S10 provides the clustering results across all 20 slices of all the compared methods). In addition, these plots (Figure 3E) also demonstrate that key marker genes such as CD163, EPCAM, and SOX4 exhibit sharper boundaries in the enhanced data, highlighting S3RL's ability to amplify biologically relevant signals. Violin plots (Figure 3F) further emphasize these improvements, showing significant differences in gene expression patterns between raw and enhanced data.

For a more detailed examination, we selected one individual slice (slice 14) and analyzed the spatial distribution of genes EPCAM, DCN, SRGN, and SOX4. As shown in Figure 3G, enhanced data provides clearer spatial patterns, confirming our model's improved sensitivity and specificity in capturing critical biological signals within lung tissue samples. Notably, EPCAM is highly expressed in lung tumors and serves as a biomarker for epithelial tumor cells [32]. In contrast, DCN acts as a tumor suppressor, with reduced expression correlating with poor prognosis in lung adenocarcinoma [33]. Additionally, SOX4 has been implicated in promoting epithelial‐to‐mesenchymal transition and tumor progression in lung cancer [34]. Building on these observations, we benchmarked S3RL's reconstruction fidelity against other methods, including GraphST, SEDR, STAGATE, and SiGra. Supplementary Figure S12 compares the gene expression reconstructions of these marker genes produced by several representative generative methods. These methods each reconstructed the data based on their own model outputs. From the visualization results, S3RL shows overall clearer spatial localization and sharper expression boundaries, indicating stronger reconstruction fidelity. While STAGATE produces comparable reconstructions for most genes, S3RL exhibits a more pronounced contrast in the spatial pattern of *SRGN*, demonstrating improved distinguishability in marker gene recovery. What's more, Supplementary Figure S13 displays the UMAP visualizations of latent embeddings across all 20 Nanostring lung slices, comparing raw and S3RL‐enhanced representations. The enhanced data reveals more compact and well‐separated clusters, indicating improved spatial structure and cell‐type distinguishability within the latent space.

Finally, to evaluate generalizability across all slices, Figure S14 illustrates the spatial distribution of these four marker genes (EPCAM, DCN, SRGN, and SOX4) across all 20 Nanostring slices, comparing both raw and S3RL‐enhanced data. The enhancement results consistently demonstrate improved gene expression resolution, reinforcing the effectiveness and generalizability of S3RL across multiple tissue samples.

We further evaluated S3RL's reconstruction fidelity by comparing Nanostring ST data with TCGA bulk RNA‐seq profiles, finding that S3RL‐enhanced data achieved higher correlation in 14 out of 20 slices (Figure 3H). Unlike other reconstruction‐based methods, which either showed minimal improvement (SEDR, SiGra) or suffered from over‐smoothing (GraphST)(Figure S15), S3RL maintained clear gene‐specific expression boundaries while improving bulk alignment, demonstrating a unique balance between biological resolution and statistical consistency(Details in Note S1 and Figures S14–S17).

### 2.5 S3RL Enhances Spatial Gene Expression and Cell–Cell Communication in Brain and Lung Tissues

**FIGURE 4:**
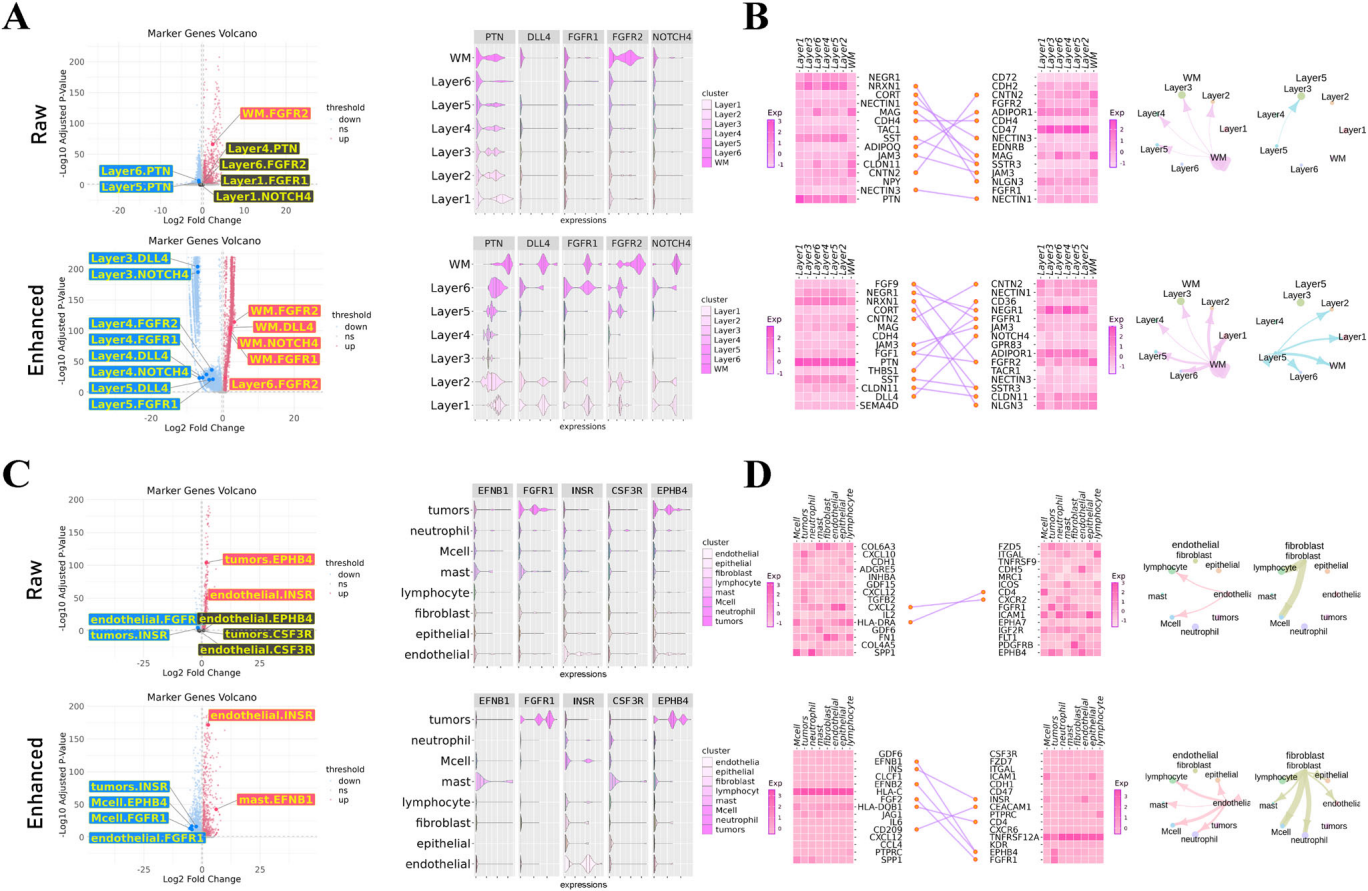
Differential Gene Expression and Cell‐Cell Communication in DLPFC Slice 151509 and Nanostring Lung Cancer Slice 001. (A) Volcano plots and violin plots for differential gene expression analysis in DLPFC slice 151509, comparing raw and S3RL‐enhanced data. The enhanced data reveals more layer‐specific expression of key marker genes such as FGFR1, FGFR2, DLL4, and NOTCH4, along with clearer stratification across cortical layers. (B) Cell‐cell communication analysis of the same DLPFC slice. The heatmaps, interaction plots, and chord diagrams show enhanced, particularly signaling in the FGF and Notch pathways after applying S3RL. (C) Volcano plots and violin plots for Nanostring lung cancer slice 001, showing improved localization and signal clarity for genes like INSR, EPHB4, and FGFR1 in the enhanced data, particularly within tumor and immune‐related clusters. (D) Cell‐cell communication analysis of the same lung slice. The heatmaps and interaction networks illustrate more pronounced tumor‐immune signaling, highlighting enhanced detection of the INS–INSR, FGF–FGFR, and EFNB–EPHB pathways following S3RL enhancement.

To assess whether S3RL's data reconstruction can recover biologically meaningful patterns masked in noisy and sparse spatial transcriptomics, we analyzed two representative datasets: human dorsolateral prefrontal cortex (DLPFC) slice 151509 and Nanostring lung cancer slice 003. We compared gene expression and inferred cell–cell communication between raw and S3RL‐enhanced data, highlighting S3RL's capacity to improve spatial resolution and biological interpretability (Figure 4).

In the DLPFC slice, S3RL‐enhanced data revealed sharper layer‐specific expression patterns for key genes such as FGFR1, FGFR2, DLL4, and NOTCH4, which are involved in cortical development and vascularization. Volcano plots (Figure 4A) and violin plots illustrated that these genes exhibited clearer localization and stronger inter‐layer differences after enhancement. Using CellChat [3], we identified strengthened ligand‐receptor interactions like DLL4‐NOTCH4 and FGF‐FGFR in the reconstructed data (Figure 4B), which are crucial for cerebral vascular formation [35, 36] and neuronal organization.

In the Nanostring lung cancer slice, S3RL enhancement enabled more precise identification of tumor‐associated transcriptional profiles and intercellular signaling within the tumor microenvironment. Genes such as INSR, FGFR1, EPHB4, and CSF3R showed clearer, cluster‐specific expression in the enhanced data (Figure 4C). Violin plots and CellChat‐based interaction networks (Figure 4D) demonstrated more defined patterns of expression and signaling, highlighting key pathways such as INS‐INSR, FGF‐FGFR, and EFNB‐EPHB. These pathways are implicated in tumor vascularization, proliferation, and immune modulation [37, 38, 39, 40] (Details in Note S2). In addition, we further compared S3RL with several representative reconstruction‐based methods, including STAGATE, stDCL, SiGra, and GraphST. As shown in Figure S18, many baseline methods exhibit over‐smoothed representations with diminished cell‐type specificity and pervasive, non‐selective cell–cell communication patterns, whereas S3RL more effectively preserves biologically coherent expression heterogeneity and selectively enhances meaningful tumor–immune and tumor–stromal signaling structures.

### 2.6 Comparison Between S3RL and SiGra in L‐R Pair Identification and Cell–Cell Communication Analysis

Accurate identification of ligand‐receptor (L‐R) interactions is essential for deciphering cell‐cell communication in single‐cell spatial transcriptomics. To construct a comprehensive and high‐confidence L‐R interaction set, we integrated candidate L‐R pairs from SiGra [21], which aggregates interactions from multiple databases, and the updated International Union of Pharmacology (IUPHAR) database [41]. After removing redundancies and ensuring uniqueness, we curated a final set of 2864 reliable L‐R interaction pairs, providing a robust foundation for downstream analyses of intercellular signaling dynamics. Among them, genes of 480 L‐R pairs are included in this NanoString CosMx dataset (189 ligands and 159 receptors).

In Figure S19, we compared S3RL against four representative enhancement‐capable methods (STAGATE, GraphST, SEDR, and SiGra) in L‐R pair significance analysis. To further quantify their capabilities in detecting functionally meaningful L‐R interactions, we further introduced three evaluation metrics and visualized them using a radar plot (bottom panel of Figure S19): the Enhanced Specificity Ratio, Effective Enhancement Percentage, and Specificity Stability Percentage. These metrics assess the tradeoff between discovering new specific interactions and preserving biologically important existing ones. Results show that both S3RL and STAGATE achieve the strongest overall performance, which further confirm the robustness of S3RL in resolving cell‐cell communication within spatial transcriptomic data.

Take SiGra method as an example, our proposed S3RL approach enhances data quality by identifying a greater number of biologically meaningful L‐R pairs while effectively reducing false positives (Figure S19). In the L‐R pair significance analysis (scatter plot), S3RL significantly increases the number of shared L‐R pairs (orange, 452 vs. 446 in SiGra) and specifically enhanced L‐R pairs (green, 21 vs. 16 in SiGra), demonstrating its capacity to reveal previously undetected but biologically relevant communication patterns. Furthermore, S3RL identifies two lung cancer‐associated L‐R interactions, ANXA1–FPR1 and OSM–LIFR, which are absent in SiGra's results. The ANXA1–FPR1 pathway is implicated in tumor immune evasion via immunosuppressive microenvironment formation [42], while OSM–LIFR signaling contributes to tumor progression through STAT3 activation and EMT induction [43]. Additionally, S3RL detects a large number of L‐R pairs at the upper right corner $\left(-\log_{10}\text{FDR}\approx 10\right)$, indicating that these ligand‐receptor interactions exhibit extremely high statistical significance (FDR close to zero) in the enhanced data, further validating their biological relevance. Moreover, other competitors retains a greater number of false‐positive L‐R pairs in the lower right quadrant, where interactions are significant in the raw data but become non‐significant after enhancement. This suggests that these methods may still preserve low‐confidence signals, whereas S3RL more effectively eliminates such noise, leading to a more precise L‐R interaction identification.

### 2.7 S3RL Reveals Spatial Architecture and Marker Gene Patterns in Neural and Tumor Tissues

To investigate how S3RL enhances the resolution of tissue architecture and amplifies biologically meaningful signals, we applied it to diverse spatial transcriptomics datasets covering neural and tumor microenvironments. These included Mouse Brain Anterior, Human Breast Cancer, Mouse Olfactory Bulb (Stereo‐seq), and Mouse Hippocampus (Slide‐seqV2), enabling us to assess S3RL's robustness across species and tissue types.

**FIGURE 5:**
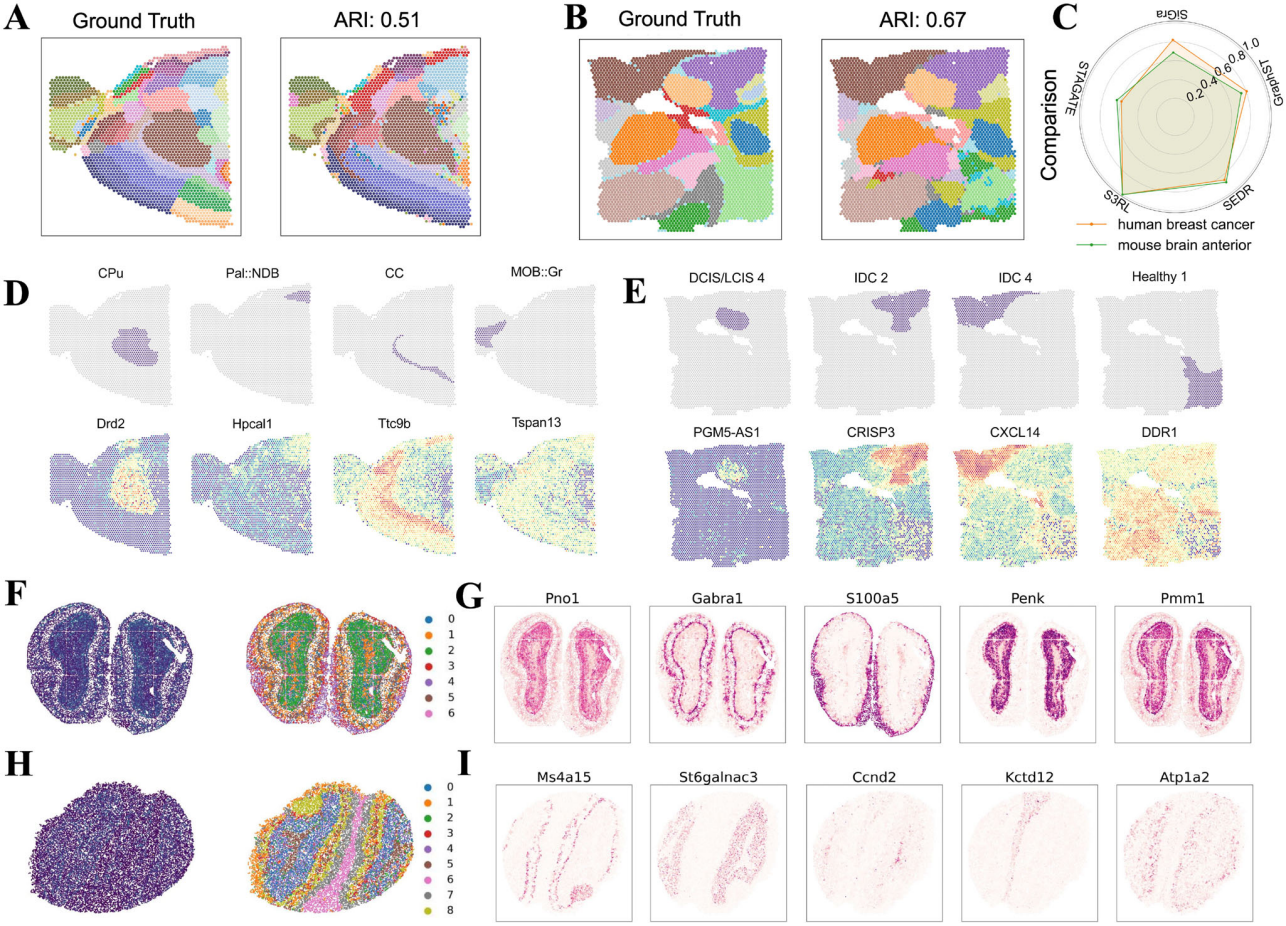
S3RL enables accurate spatial domain segmentation and functional annotation in neural and tumor tissues. (A,B) Segmentation results on the mouse brain anterior and human breast cancer datasets. S3RL accurately reconstructs spatial domains aligned with ground truth annotations, achieving Adjusted Rand Index (ARI) scores of 0.51 and 0.67, respectively. Distinct cortical and subcortical structures in the brain and tumor‐stroma boundaries in breast cancer are effectively captured. (C) Radar plot comparison of ARI scores across GraphST, SiGra, STAGATE, SEDR, and S3RL on both datasets, normalized to S3RL's performance (set to 1.0). (D,E) Spatial gene expression patterns of key marker genes. In the mouse brain dataset, genes such as Drd2, Hpcal1, Ttc9b, and Tspan13 show region‐specific localization. In the breast cancer dataset, markers including PGM5‐AS1, CRISP3, CXCL14, and DDR1 reveal distinct enrichment across tumor microenvironmental regions. (F‐G) Expression of marker genes in the mouse olfactory bulb (Pno1, Gabra1, S100a5, Penk, Pmm1) and hippocampus (Ms4a15, St6galnac3, Ccnd2, Kctd12, Atp1a2), highlighting S3RL's ability to resolve fine‐grained anatomical subregions. (H‐I) Gene ontology enrichment analysis for hippocampal clusters identifies biological processes such as cognition, memory, and synapse assembly (clusters 2, 6, and 7), while olfactory bulb clusters are enriched in mitochondrial ATP synthesis and synaptic remodeling pathways. These results demonstrate S3RL's effectiveness in reconstructing spatial domains, enhancing gene expression resolution, and uncovering functional tissue architecture in complex neural and tumor environments.

In the Mouse Brain Anterior dataset (Figure 5A), S3RL accurately reconstructed cortical and subcortical regions, achieving an Adjusted Rand Index (ARI) of 0.51. Similarly, in Human Breast Cancer tissues (Figure 5B), S3RL obtained an ARI of 0.67, effectively delineating tumor, stromal, and immune‐enriched regions. The radar plot (Figure 5C) highlights S3RL's consistent superiority or comparable performance relative to GraphST, SiGra, STAGATE, and SEDR across datasets (Figure S20).

Beyond tissue segmentation, S3RL‐enhanced data revealed clearer marker gene spatial patterns. In Mouse Brain Anterior (Figure 5D), genes like Drd2 and Hpcal1 showed distinct enrichment in caudoputamen (CPu) and basal forebrain regions, respectively, aligning with their known roles in dopaminergic signaling and neuronal calcium regulation [44, 45]. In Human Breast Cancer (Figure 5E), genes such as CXCL14 (tumor suppressor) and DDR1 (associated with poor prognosis) [46, 47] displayed sharper and more interpretable spatial expression compared to raw data. Figures S21 and S22 further illustrate that baseline compared models often produce diffuse or blurred spatial patterns such as GraphST, SiGra etc., highlighting S3RL's advantage in recovering fine‐grained gene expression landscapes in both neural and tumor tissues.

We further applied S3RL to the Mouse Olfactory Bulb and Hippocampus datasets (Figure 5F–I). Spatial clustering uncovered layered structures and functional zones validated by marker genes. In the olfactory bulb, genes such as Gabra1 (GABA receptor subunit) and S100a5 (neurogenesis marker) [48, 49] highlighted mitral and glomerular layers. In the hippocampus, Ccnd2 (linked to neurogenesis) and Atp1a2 (Na+/K+ ATPase) distinguished dentate gyrus and CA regions [50, 51]. These clear spatial patterns, absent in raw data, demonstrate S3RL's capability to reconstruct biologically meaningful structures and amplify subtle expression signals critical for functional annotation. The improved spatial fidelity across neural and tumor tissues underscores S3RL's potential to enhance interpretability in both developmental biology and cancer research.

### 2.8 S3RL Reveals Functional Landscapes and Regulatory Trajectories in Human Lung Cancer

**FIGURE 6:**
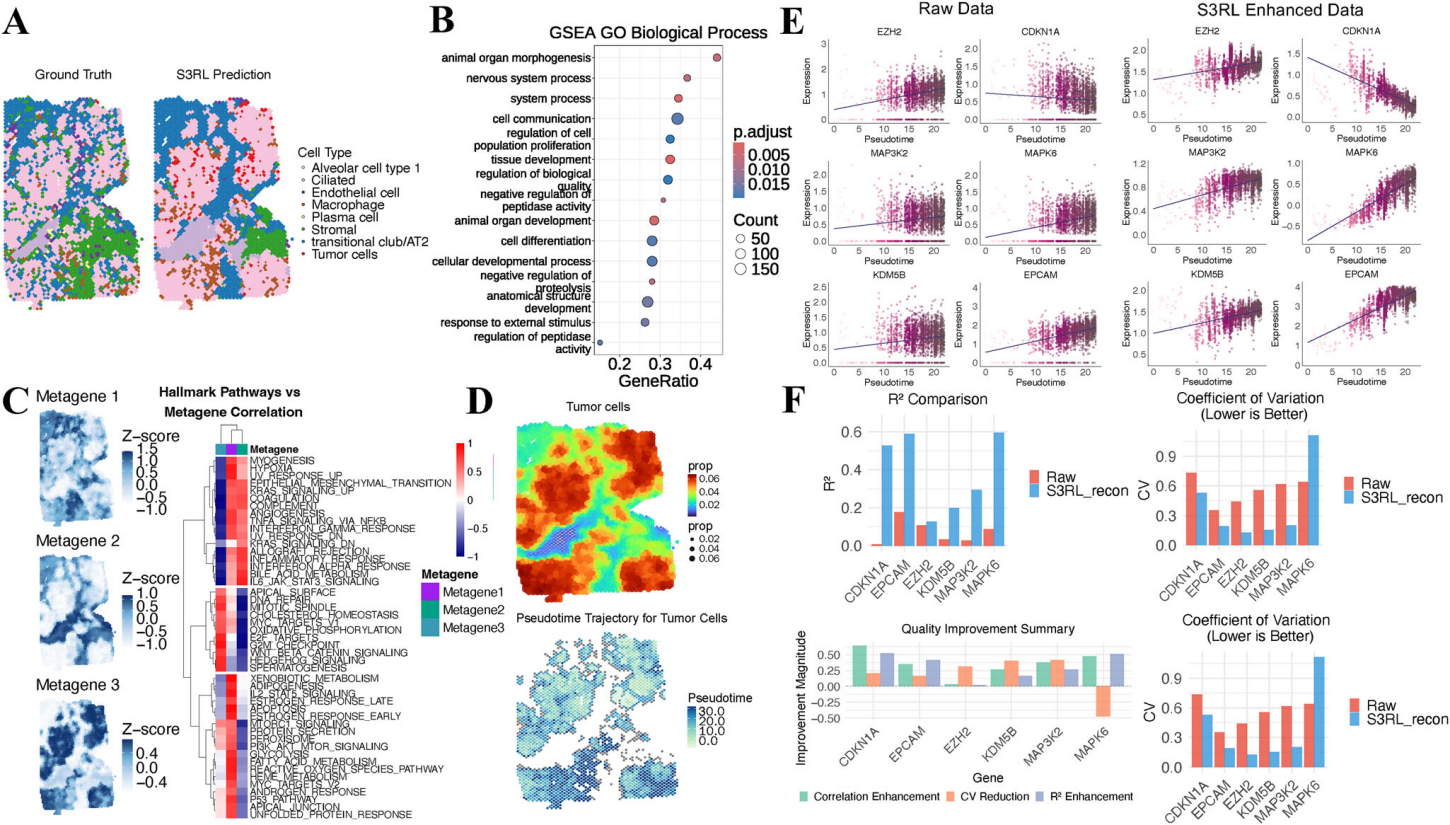
Functional zonation and regulatory trajectory revealed in human lung cancer by S3RL. (A) Comparison of ground truth cell type annotations (left) and S3RL‐predicted cell type distributions (right) in the 10X Visium human lung cancer dataset, showing accurate reconstruction of tumor microenvironment architecture. (B) Gene Set Enrichment Analysis (GSEA) of tumor‐associated spatially variable genes (SVGs) identified by CELINA on S3RL‐enhanced data, revealing activation of cancer‐related biological processes. (C) Three distinct metagenes with unique spatial expression patterns and enriched pathways, illustrating functional zonation within tumor regions. (D) Spatial cell type proportion estimation using CARD and pseudotime trajectory inference from S3RL‐derived latent embeddings using Monocle3. (E) Expression dynamics of key gene pairs along the inferred pseudotime, showing enhanced temporal coherence and regulatory relationships in S3RL‐reconstructed data. (F) Quantitative evaluation of biological signal recovery using $R^{2}$, Pearson correlation, and coefficient of variation (CV), where S3RL consistently improves temporal fitting, developmental coherence, and reduces noise.

Building on S3RL's ability to resolve complex tissue architectures across diverse neural and tumor datasets, we next examined its performance at a finer resolution to assess how well it reconstructs subtle biological signals. To this end, we performed in‐depth analyses on selected human and mouse datasets. As a first step, we applied S3RL to map functional landscapes and regulatory dynamics within 10X Visium human lung cancer tissue. As demonstrated in Figure 6A, S3RL accurately reconstructed the spatial architecture of the tumor tissue, with predicted cell type distributions closely matching the ground truth annotations. The model successfully delineated distinct cellular compartments including tumor cell clusters, infiltrating macrophages, vascular endothelial cells, and surrounding stromal regions, preserving the native spatial organization of the lung cancer microenvironment.

Figure 6B presents Gene Set Enrichment Analysis (GSEA) of tumor‐associated spatially variable genes (SVGs) identified through CELINA [52] analysis on S3RL‐reconstructed data. The functional enrichment analysis revealed significant activation of key cancer‐related biological processes, including animal organ morphogenesis, nervous system processes, epithelial‐mesenchymal transition, cell differentiation, and cellular developmental processes. These enriched pathways demonstrate that S3RL successfully captures the fundamental biological processes underlying tumor spatial organization and progression. A detailed visualization of GSEA enrichment curves for representative pathways such as cell adhesion, cell differentiation, and cell‐cell signaling is provided in Figure S23, highlighting their relevance to tumor progression and microenvironment remodeling.

Based on these tumor‐associated SVGs, we further performed kmeans clustering analysis to group them into three functional modules (metagenes) according to their spatial expression similarity. Figure 6C shows these three metagenes, each exhibiting a unique spatial expression pattern corresponding to specific regions. Metagene 1 (purple regions) demonstrated significant enrichment in epithelial‐mesenchymal transition (EMT), KRAS signaling, hypoxia response, angiogenesis, TNF‐α/NF‐κB signaling, interferon responses, and inflammatory pathways, indicating regions of enhanced tumor invasiveness and active immune‐tumor interactions where cancer cells undergo phenotypic transitions. Metagene 2 (green regions) showed selective enrichment primarily in apical surface organization and early developmental pathways while exhibiting marked depletion across most metabolic processes, suggesting immune‐infiltrated regions or transitional zones with maintained epithelial characteristics but reduced proliferative activity. Metagene 3 (blue regions) exhibited strong enrichment in proliferation‐associated pathways including DNA repair, mitotic spindle organization, G2M checkpoint, E2F targets, and MYC targets, coupled with metabolic reprogramming signatures such as oxidative phosphorylation, glycolysis, fatty acid metabolism, and mTORC1/PI3K‐AKT‐mTOR signaling, as well as developmental pathways like WNT/β‐catenin and Hedgehog signaling. This expression profile characterizes metabolically hyperactive tumor regions with high proliferative potential that may represent areas primed for malignant progression or aggressive tumor growth. Importantly, this spatial‐functional zonation revealed by S3RL‐enhanced data provides insights into potential future tumor differentiation trajectories, distinguishing between immune‐infiltrated transitional zones (Metagene 2) and metabolically active proliferative regions (Metagene 3) that may drive tumor evolution and therapeutic resistance. Supporting evidence from recent spatial transcriptomic studies in lung cancer has identified similar microenvironment niches, such as invasive fronts and immune‐rich regions, which are strongly associated with prognosis and response to immunotherapy [53].

Figure 6D presents spatial cell type proportion estimation using CARD [54] on S3RL‐reconstructed data and pseudotime trajectory inference using Monocle3 [55] on the S3RL‐derived latent embeddings. The CARD‐based deconvolution revealed spatially coherent tumor cell proportion patterns across the tissue, while the reconstructed pseudotime trajectories suggested biologically plausible developmental progressions, showing clear spatial organization of tumor cell states from early to advanced stages. A detailed UMAP visualization of pseudotime values and cluster labels is provided in Supplementary Figure S24.

To investigate the underlying regulatory mechanisms, Figure 6E examines the expression dynamics of key gene pairs along the inferred pseudotime axis. Compared to raw data, S3RL‐enhanced profiles exhibited markedly improved temporal coherence and expression gradient consistency. The enhanced signal clarity revealed several important regulatory relationships: COL1A2‐ITGA3 interactions facilitate extracellular matrix (ECM) remodeling and cell adhesion, promoting tumor cell migration and invasion through ECM‐receptor interactions [56] and PI3K‐Akt signaling pathways as documented in pancreatic ductal adenocarcinoma studies. TGFBI‐COL1A2 regulatory axis reflects TGF‐β‐induced ECM remodeling, where TGFBI enhances COL1A2 expression to increase tumor invasiveness. The SPARC‐COL1A2 interaction involves matrix‐associated protein regulation of collagen deposition, affecting tumor growth and metastasis [57]. Additionally, SPP1‐ITGB4 binding (osteopontin‐integrin interaction) activates downstream signaling pathways promoting tumor cell migration, while MAP3K2‐MAPK6 interactions within the MAPK signaling cascade regulate cell proliferation and survival. The KDM5B‐EPCAM regulatory relationship involves histone demethylase‐mediated epigenetic control of epithelial cell adhesion molecule expression, influencing tumor stem cell properties and differentiation states [58].

To quantitatively assess the biological signal recovery, Figure 6F evaluates three complementary metrics: (i) coefficient of determination ($R^{2}$) measuring the proportion of expression variance explained by pseudotime progression, indicating how well cellular dynamics account for transcriptional changes; (ii) Pearson correlation with pseudotime, reflecting the monotonic relationship between gene expression and developmental progression; (iii) coefficient of variation (CV), quantifying expression noise levels, where lower values indicate improved signal‐to‐noise ratios.

S3RL consistently outperformed raw data across all three metrics, demonstrating superior temporal fitting (higher $R^{2}$), stronger developmental coherence (increased correlation), and reduced technical noise (lower CV). These improvements collectively validate S3RL's effectiveness in extracting latent biological regulatory programs from noisy spatial transcriptomic measurements, thereby enhancing the reliability of downstream developmental and regulatory analyses.

### 2.9 Spatial Gene Expression Patterns Around Amyloid Plaques Revealed by S3RL Reconstruction

**FIGURE 7:**
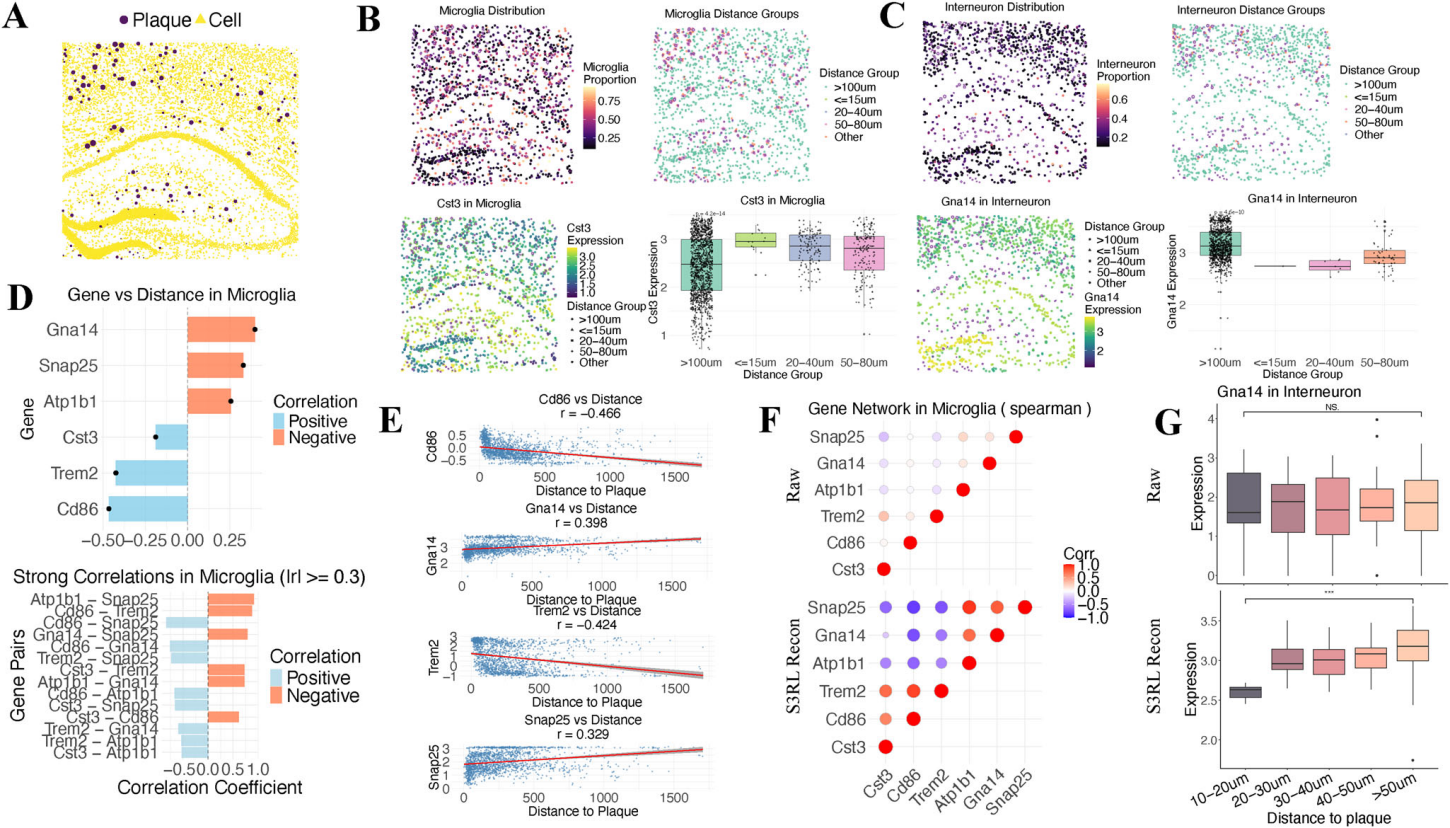
S3RL reconstruction reveals spatial gene expression gradients and co‐expression networks in microglia and interneurons around amyloid plaques. (A) Spatial map of plaques (purple) and cells (yellow) in the mouse hippocampus. (B) Microglial spatial distribution and Cst3 expression. Left: microglia proportion across the hippocampus. Middle: microglia grouped by plaque proximity. Right: Cst3 expression in microglia across distance groups, showing significant upregulation near plaques (≤15μm). (C) Interneuron spatial distribution and Gna14 expression. Left: interneuron proportion across the hippocampus. Middle: interneurons grouped by plaque proximity. Right: Gna14 expression levels in interneurons, showing significant downregulation in plaque‐proximal regions. (D) Correlations of selected genes with plaque distance in microglia (top) and strong gene co‐expression pairs (|r|≥0.3, bottom). Blue and orange bars represent positive and negative correlations, respectively. (E) Scatter plots of gene expression versus plaque distance for Cd86, Gna14, Trem2, and Snap25, showing clear trends in S3RL‐reconstructed data. (F) Gene co‐expression networks in microglia derived from raw (top) and S3RL‐reconstructed (bottom) data. Nodes represent genes; edges are colored by Spearman correlation coefficients. S3RL reconstruction enhances the clarity and density of co‐expression relationships. (G) Boxplots comparing Gna14 expression in interneurons across plaque distance groups in raw data (top) and S3RL‐reconstructed data (bottom). S3RL reconstruction uncovers significant spatial expression differences that are undetectable in raw data. Together, these results highlight S3RL's ability to reveal subtle but biologically meaningful spatial expression gradients and regulatory networks in noisy spatial transcriptomics datasets.

Next, by using the STARmap‐PLUS spatial transcriptomics dataset of the mouse hippocampus [52], we systematically analyzed the spatial distribution of different cell types within amyloid plaque regions and their relationship with specific gene expression patterns (Figure 7). Figure 7A shows the spatial distribution patterns of microglia and inhibitory interneurons within the tissue. Notably, microglia exhibited significant enrichment in regions proximal to plaques (≤15μm), displaying pronounced chemotactic aggregation, while interneurons were predominantly distributed in distal regions (>100μm from plaques), suggesting that the plaque microenvironment exerts differential effects on distinct neural cell populations. However, in the raw data, these patterns were less apparent, and in interneurons, the spatial distribution even showed trends contrary to existing biological knowledge (Figure S25).

In the analysis of spatial gene expression patterns, S3RL reconstructed data demonstrated significantly superior detection capabilities compared to raw data (Figures S26 and S27). Figure 7B focused on the Cst3 gene in microglia, where S3RL reconstruction clearly revealed a strong negative correlation between gene expression levels and plaque distance (p = 4.2$e^{-14}$), indicating higher Cst3 expression in closer proximity to plaques. Cst3 encodes cystatin C, a cysteine protease inhibitor, and its upregulation likely reflects the activated state of microglia in plaque‐adjacent regions and enhanced protease regulation functions, which consists with previous work [59].

Similarly, Figure 7C illustrates the spatial expression pattern of the Gna14 gene in interneurons. S3RL reconstructed data revealed significantly decreased expression of this gene in plaque‐proximal regions (p = 4.6$e^{-10}$), suggesting potential suppression of G‐protein signaling in the plaque microenvironment. Critically, this biologically significant spatial regulatory pattern was barely detectable in raw data, while the S3RL method successfully captured these subtle yet biologically meaningful expression gradients through effective denoising and signal enhancement [60].

Figure 7D,E further quantifies the correlations between multiple key genes and plaque distance. In microglia, inflammatory activation markers such as Cd86 (r = ‐0.466), Trem2 (r = –0.424), and Cst3 all exhibited significant negative correlations with plaque distance, while in interneurons, synaptic function‐related genes including Gna14 and Snap25 (r = 0.329) showed positive correlations. These results revealed a spatial segregation pattern of inflammatory activation and neural function suppression in the peri‐plaque microenvironment.

To investigate coordinated regulatory relationships among these genes, Figure 7F constructed a gene co‐expression network for microglia (based on Spearman correlation coefficients, |r|≥0.3). Figure S28 provides additional comparisons of gene–gene co‐expression networks between raw and S3RL‐reconstructed data for both microglia and interneurons. In raw data, minimal detectable correlations were observed, with sparse co‐expression patterns and only a single gene pair (Cst3–Trem2) surpassing the threshold. By contrast, S3RL‐enhanced data revealed multiple strongly correlated gene pairs forming distinct co‐expression modules, particularly among inflammatory marker genes. Notably, negative correlations between inflammatory activation genes and synaptic function genes suggest antagonistic neuro‐immune interactions within the plaque microenvironment. This observation not only provides mechanistic insights into how inflammatory responses may suppress neuronal functionality in neurodegenerative settings but also highlights potential therapeutic opportunities for simultaneously modulating microglial activation and synaptic preservation. The ability of S3RL to recover these subtle yet critical regulatory patterns underscores its utility in identifying actionable targets from noisy spatial transcriptomics data.

Figure 7G illustrates the enhanced resolution of S3RL in detecting spatial gene expression trends. By stratifying spots into distance‐based groups from plaques (0–10, 10–20, 20–40, 40–80, >100μm), we assessed Gna14 expression in interneurons. While raw data showed weak and inconsistent distance‐related differences, S3RL‐reconstructed data revealed a clear trend of higher Gna14 expression in plaque‐distal regions (>40μm), with robust statistical significance and reduced variance. This improvement underscores S3RL's ability to amplify subtle biological gradients and reduce technical noise, enabling more reliable interpretation of spatial regulatory patterns.

The superior performance of the S3RL method in revealing spatial gene expression patterns around plaques not only improved data quality but, more importantly, uncovered potential biological regulatory mechanisms that were masked by noise in raw data. These results indicate the existence of distinct spatial molecular gradients in Alzheimer's disease pathological environments: plaque‐proximal regions are dominated by microglial activation and inflammatory responses, while plaque‐distal regions maintain relatively normal neuronal functions. Through effective integration of spatial neighborhood information and intrinsic correlations in gene expression, the S3RL method successfully reconstructed this complex spatial molecular landscape, providing a more precise and reliable analytical tool for understanding the pathological mechanisms of neurodegenerative diseases.

### 2.10 Spatial Transcriptional Regulatory Networks in Soybean Cotyledon Stage Seeds

**FIGURE 8:**
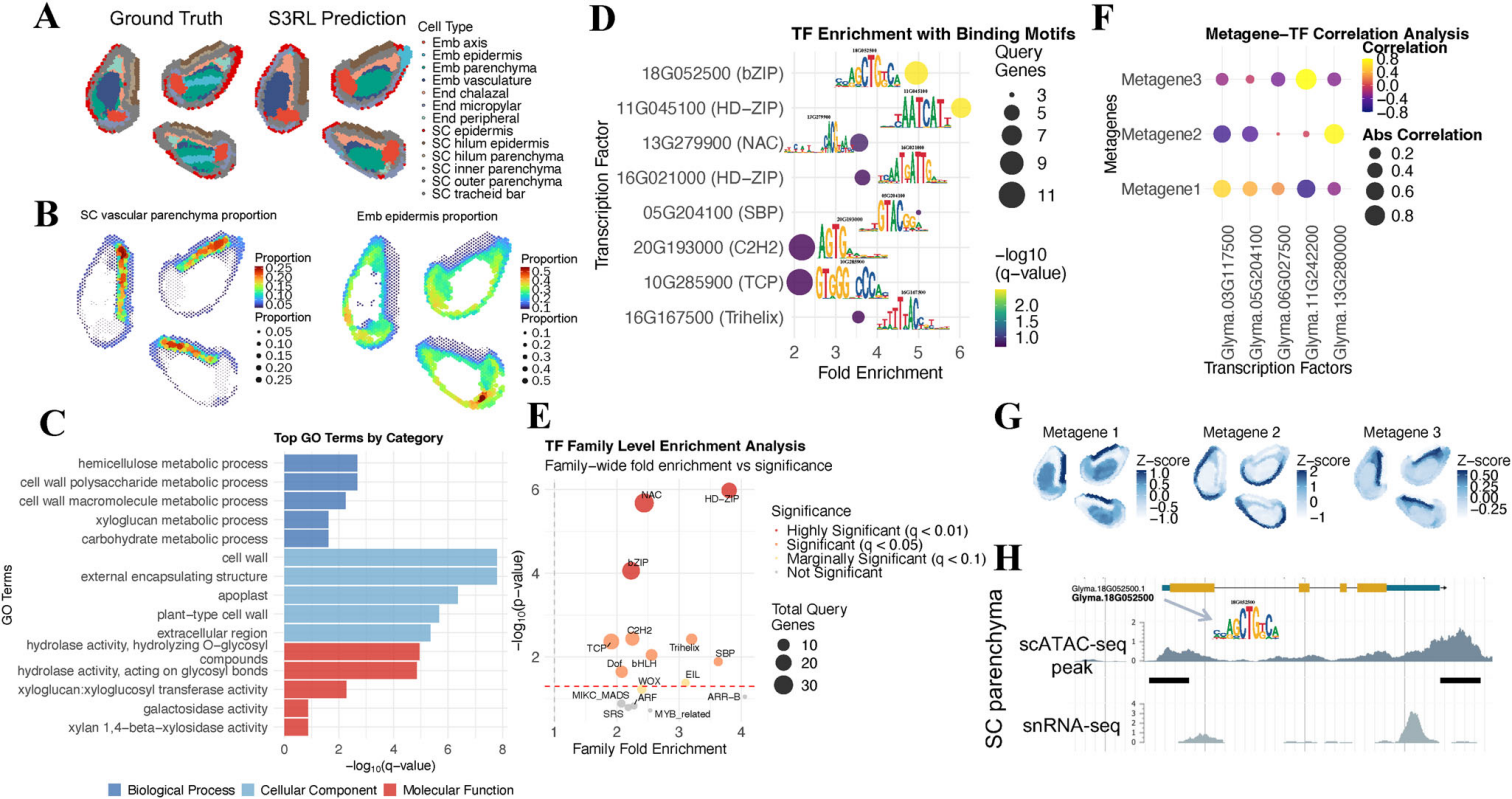
S3RL reconstruction supports functional enrichment and transcriptional regulatory network analysis in soybean cotyledon stage seeds. (A) Spatial clustering results of soybean cotyledon stage seeds. Ground truth (left) and S3RL predictions (right) show high concordance across 11 annotated tissue types, including embryonic axis, parenchyma, and vascular bundles. (B) Spatial deconvolution analysis using CARD on S3RL‐reconstructed data reveals the proportion of SC vascular parenchyma and embryonic epidermis, showing distribution patterns consistent with expected anatomical locations. (C) GO enrichment analysis of differentially expressed genes identified from S3RL data highlights biological processes related to cell wall organization, polysaccharide metabolism, and structural macromolecule biosynthesis–key functional attributes of SC vascular parenchyma cells. (D) Top 8 transcription factors enriched in the query gene set, with fold enrichment, motif logos, and significance. (E) Transcription factor family‐level enrichment analysis shows highly significant enrichment for HD‐ZIP, NAC, and bZIP families, all of which play critical roles in vascular tissue differentiation and stress responses. (F) Correlation analysis between metagene expression and transcription factors reveals spatially distinct regulatory relationships; Metagene 3 shows the strongest correlation with Glyma.11G242200, a regulator involved in water deficit responses. (G) Spatial expression patterns of three metagenes derived from k‐means clustering of DEGs, illustrating distinct expression zones across cotyledon tissues. (H) Epigenomic validation of Glyma.18G052500 (bZIP) using scATAC‐seq and snRNA‐seq data from the Plant Epigenome Browser. Open chromatin regions identified in scATAC‐seq align with transcriptional activity detected in snRNA‐seq, supporting active regulation at this locus. Together, these results demonstrate S3RL's ability to produce high‐fidelity data suitable for functional genomics and regulatory network discovery in plant tissues.

To further validate the effectiveness of the S3RL method in plant tissue spatial transcriptomics analysis, we conducted a systematic analysis of soybean embryonic development samples from a recent work (Figure 8) [61]. Figure 8A demonstrates the results of S3RL clustering analysis. By comparing the ground truth annotations with S3RL predictions, we observed that our model accurately identifies and distinguishes different tissue types, including embryonic axis, embryonic epidermis, parenchyma cells, vascular bundles, and other key anatomical structures, validating its excellent discriminative capability in complex plant tissues.

Using the CARD tool for cell type deconvolution analysis on S3RL reconstructed data, Figure 8B revealed the spatial distribution patterns of two key cell types: SC vascular parenchyma and embryonic epidermis. SC vascular parenchyma cells were predominantly distributed in the vascular bundle regions of the embryo, exhibiting a distinct linear distribution pattern, while embryonic epidermis cells were mainly located in the outer layers of the embryo, forming a protective barrier. These patterns were highly consistent with the tissue structure predictions in Figure 8A, further confirming S3RL's reconstruction accuracy.

Based on high‐quality data reconstructed by S3RL, we successfully identified spatially specific differentially expressed genes in SC vascular parenchyma cells using the CELINA tool. Functional enrichment analysis of these genes, shown in Figure 8C, revealed significant enrichment in cell wall‐related processes, including hemicellulose metabolic process, cell wall polysaccharide metabolic process, and xyloglucan metabolic process(Figure S29). These findings align with the structural support and material transport functions of vascular parenchyma cells–hemicelluloses such as xyloglucan form a network with cellulose microfibrils, contributing to cell wall strength and extensibility [62], demonstrating that S3RL enhances the biological interpretability of plant spatial transcriptomics data.

In transcriptional regulatory network analysis, we identified 33 transcription factors (TFs) with significant target gene enrichment via PlantRegMap [63](Figure 8D and Figure S30). Among these, Figure 8D highlights the top eight TFs with the highest enrichment fold changes and their corresponding binding motifs, including 18G052500 (bZIP), 11G045100 (HD‐ZIP), 13G279900 (NAC), and others(Figure S31). The identification of these TFs benefited from S3RL's ability to denoise raw data and amplify biologically relevant signals, enabling precise capture of key regulators in vascular parenchyma cells.

Additionally, Figure 8E presents a family‐level TF enrichment analysis, showing that HD‐ZIP, NAC, and bZIP families were highly enriched. These families are known to play crucial roles in vascular bundle development, secondary cell wall formation, and stress response regulation [64, 65]. The alignment between predicted TF functions and known biology underscores S3RL's ability to generate high‐fidelity data for plant systems.

To further explore spatial regulatory patterns, we performed k‐means clustering on differentially expressed genes to define three metagenes (Figure 8G). These metagenes showed distinct spatial expression profiles: Metagene1 was highly expressed in specific embryonic regions, Metagene2 displayed a gradient distribution, and Metagene3 was enriched around vascular bundles. The correlation between metagene expression and TF expression, analyzed in Figure 8F, revealed strong associations. For example, Metagene1 showed a positive correlation with Trihelix TFs, linked to cell wall organization, while Metagene3 was highly correlated with TF 11G242200, implicated in ABA signaling regulation and water stress responses.

Finally, epigenomic validation of TF 18G052500 using the Plant Epigenome Browser (Figure 8H) [66] confirmed consistency between scATAC‐seq open chromatin peaks and snRNA‐seq expression signals, supporting the regulatory role of this TF in vascular parenchyma cells. This highlights S3RL's utility in decoding plant tissue molecular mechanisms during development.

### 2.11 Clustering Performance under Multi‐Slice Alignment

In spatial transcriptomics analysis, aligning and integrating multiple tissue slices is essential for reconstructing coherent tissue architectures and capturing biologically meaningful spatial patterns across sections. This step is particularly challenging due to technical variability, incomplete slices, and the inherent heterogeneity of biological tissues. Notably, S3RL's ability to reconstruct high‐fidelity spatial features provides an ideal foundation for robust multi‐slice alignment.

Among existing methods, GraphST has been one of the few designed to perform multi‐slice alignment. However, its alignment capability is restricted to fully paired slices and often fails in the presence of partial data. In contrast, S3RL supports both complete and partially missing slices, leveraging its enhanced spatial representations to improve alignment accuracy and clustering consistency. Here, we systematically compare the multi‐slice clustering performance of S3RL and GraphST under two conditions: full‐slice alignment and partial‐slice alignment.

S3RL first reconstructs refined gene expression profiles, allowing adjacent tissue slices to be embedded into a unified coordinate space. This spatial unification enables joint clustering across slices, where biologically similar regions from different sections are aligned and integrated into a single spatial graph. By incorporating context from neighboring slices, S3RL enhances the definition of structural boundaries and improves clustering fidelity.

#### 2.11.1 Clustering Performance on Complete Slices

**FIGURE 9:**
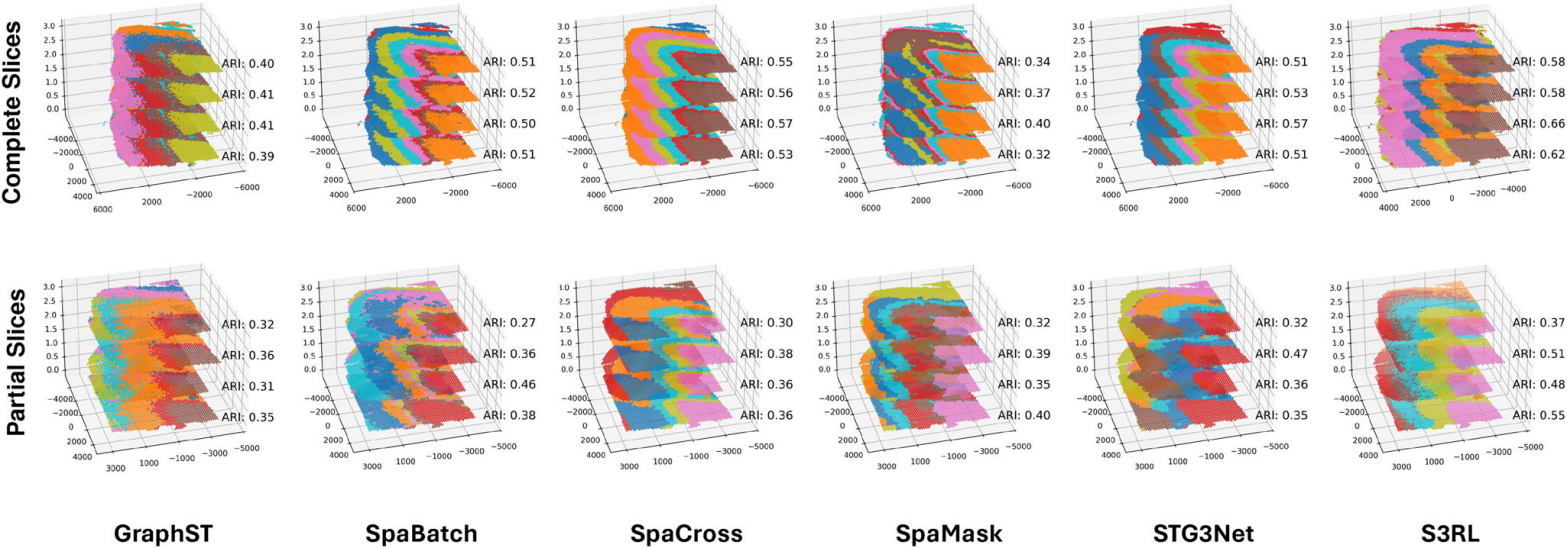
Multi‐Slice Clustering Performance Evaluation. Top Row (Complete Slices): Comparison of spatial clustering performance on four complete tissue slices. From left to right, the panels display the predicted clustering from GraphST, SpaBatch, SpaCross, SpaMask, STG3Net, and S3RL. S3RL achieves higher Adjusted Rand Index (ARI) scores (0.58–0.62) compared to other methods, demonstrating superior preservation of spatial continuity and biological structure. Bottom Row (Partial Slices): Evaluation of robustness using datasets with 30% partial slice removal. Panels display results for GraphST, SpaBatch, SpaCross, SpaMask, STG3Net, and S3RL. While most baseline methods show significant performance degradation and misaligned boundaries under data loss conditions, S3RL maintains high alignment accuracy and ARI scores, highlighting its robustness in handling incomplete spatial transcriptomics data.

We first evaluated the alignment of four complete 10X Visium slices. Here GraphST, SpaBatch [67], SpaCross [68], SpaMask [69], STG3Net [70] are employed as competitors. As illustrated in Figure 9, S3RL achieves the highest overall clustering accuracy, with mean ARI values exceeding 0.60 across slices. SpaCross and STG3Net exhibit good performances, while GraphST and SpaMask perform well in certain slices but produce less spatially coherent boundaries.

#### 2.11.2 Robustness to Partial Slice Removal

To simulate real‐world datasets where slices may be incomplete or have limited overlap, we randomly cropped 30% of each slice before alignment and clustering. This setting presents greater challenges for preserving spatial continuity. The advantage of S3RL becomes more apparent under the partial‐slice condition, where methods such as SpaBatch and SpaCross experience substantial degradation due to the loss of spatial context. In contrast, S3RL maintains stable performance, suggesting that its Semantic Information Extraction module and dynamic prototype learning effectively mitigate the impact of missing regions by leveraging intrinsic biological and morphological patterns. Besides, S3RL also benefits from S3RL's high‐quality data reconstruction, which clarifies spatial structures and amplifies biologically relevant signals. The enhanced spatial context enables the model to effectively integrate and align information across heterogeneous tissue sections, supporting downstream analyses such as multi‐slice trajectory inference and region‐specific functional annotation. Finally, we examined SpaViT [71], SpaDiT [72], and scstGCN [73]. These methods are based on supervised or semi‐supervised learning and rely on external labels, whereas S3RL is designed as a fully unsupervised framework for spatial domain inference. Owing to this fundamental difference in task formulation, direct quantitative comparisons using ARI or similar unsupervised clustering metrics are not methodologically appropriate.

## 3 Discussion

Spatial transcriptomics has transformed our ability to study tissue organization by providing spatially resolved gene expression profiles, bridging the gap between molecular states and tissue morphology. Yet, existing analytical frameworks face persistent challenges in extracting meaningful multimodal features and reconstructing biologically faithful spatial representations. First, histological images–despite being a rich source of spatial context–often exhibit high structural homogeneity and blurred region boundaries, limiting their contribution to downstream analyses. Second, most graph construction strategies rely solely on spatial proximity (e.g., Euclidean distance or k‐nearest neighbors), overlooking functional relationships between spatially distant but biologically similar spots and sometimes introducing noise from adjacent but distinct tissue regions. Finally, the absence of explicit constraints on representation separability in latent space often leads to overlapping clusters and poor boundary definitions, compromising biological interpretability and robustness.

In this study, we present S3RL, a unified spatial transcriptomics framework that synergizes graph neural networks (GNNs) with hyperspherical prototype clustering. By integrating gene expression, spatial coordinates, and high‐level semantic features from histological images, S3RL reconstructs refined spatial landscapes that enable more accurate cell‐type clustering and spatial structure delineation. Notably, S3RL is not just an enhancement of existing graph‐based models or a conventional denoising approach. Instead, it introduces a representation learning paradigm where dynamic prototype updates and hyperspherical constraints ensure that high‐dimensional expression features are reorganized into biologically meaningful, low‐dimensional embeddings. This design allows S3RL to achieve high‐fidelity spatial reconstructions, which in turn enhance downstream analyses such as cross‐slice alignment and intercellular signaling inference.

We validated S3RL's performance across diverse spatial transcriptomics datasets, including 10X DLPFC, Nanostring lung cancer, 10X human lung cancer, human breast cancer, mouse brain (10X Visium), mouse hippocampus (Slide‐seqV2), soybean cotyledon (10X Visium), and mouse olfactory bulb (Stereo‐seq). Across these datasets, S3RL consistently outperformed state‐of‐the‐art methods like GraphST, STAGATE, and BayesSpace, achieving average ARI improvements of 26%–170%. Its reconstructed data demonstrated clearer tissue segmentation, sharper expression boundaries, and enhanced biological signal recovery. For instance, in DLPFC data, S3RL revealed more distinct cortical layers and improved cell‐type deconvolution accuracy. In Nanostring lung cancer slices, S3RL‐enhanced data uncovered robust ligand‐receptor signaling networks and refined tumor microenvironment mapping. Notably, S3RL recovered biologically relevant spatial patterns–such as immune‐tumor interactions and localized regulatory programs–that were previously obscured by noise in raw data. Some of these findings align with reported experimental evidence, highlighting S3RL's capability to not only restore known biological structures but also discover novel insights across human, animal, and plant tissues. The multi‐slice alignment experiments further underscore the strength of S3RL's reconstruction. By providing more coherent spatial representations, S3RL facilitates accurate tissue section integration even when slices are partially missing–a common challenge in real‐world datasets. This robustness is particularly advantageous for building 3D tissue atlases and studying complex developmental processes.

Despite these advances, several aspects warrant further optimization. First, while histological features contribute to S3RL's improved performance, more sophisticated strategies such as self‐supervised or multimodal contrastive learning could enhance their generalizability across datasets. Second, in datasets with extreme sparsity or low coverage (e.g., Slide‐seq), additional graph‐based regularization may further stabilize the learned representations. Finally, extending S3RL to jointly model multi‐omics spatial data–including proteomics and epigenomics–represents a promising future direction. In addition, integrating single‐cell RNA sequencing (scRNA‐seq) information more directly into the S3RL training process represents another promising extension. While our current analyses already demonstrate that S3RL‐enhanced spatial representations substantially improve downstream scRNA‐seq–guided deconvolution, future versions of the framework may incorporate joint representation learning or cross‐modal prototype alignment to allow single‐cell transcriptomic variation to explicitly shape the latent space during training. Such integration has the potential to further enhance clustering accuracy, refine spatial domain boundaries, and strengthen the biological interpretability of reconstructed spatial landscapes.

In summary, S3RL provides a biologically meaningful, robust, and versatile computational framework that enhances spatial transcriptomics analysis through high‐fidelity reconstruction. By amplifying latent biological signals and improving spatial resolution, S3RL sets the stage for deeper exploration of tissue organization and intercellular communication across diverse biological systems, from human disease to plant development.

## 4 Method

The proposed S3RL framework consists of three main components: Semantic Information Extraction Module, Signed Edge Extraction based Graph Construction Module, and Separable Representation Learning Module. We first introduce the data preprocessing and notations, followed by detailed descriptions of each module.

### 4.1 Data Preprocessing and Notations

We evaluated S3RL on a wide range of spatial transcriptomics datasets generated by multiple platforms to demonstrate its generalizability across species, tissue types, and technological resolutions. Specifically, we included (1) the NanoString CosMx SMI lung cancer dataset (Lung‐91), which provides high‐resolution single‐molecule imaging data of formalin‐fixed, paraffin‐embedded (FFPE) human lung tissues; (2) the Vizgen MERSCOPE mouse liver dataset L1R1, representing MERFISH‐based high‐plex spatial profiling; (3) 10X Visium datasets from the human dorsolateral prefrontal cortex (DLPFC), breast cancer tissues, and human lung cancer FFPE sections; (4) mouse brain anterior and hippocampus slices (10X Visium and Slide‐seqV2, respectively), and (5) plant tissue datasets such as soybean cotyledon stage seeds (10X Visium). These datasets cover both animal and plant tissues, normal and diseased conditions, and capture diverse spatial resolutions, ranging from spot‐based measurements (Visium) to subcellular single‐molecule resolution (CosMx and MERSCOPE). For all datasets except Lung‐91, we used scanpy.pp.highly_variable_genes to select 3,000 highly variable genes to construct the input expression matrices for training. All datasets processed in this study are bona fide spatial transcriptomics datasets with explicit spatial coordinates, ensuring that S3RL is evaluated exclusively on spatially resolved gene expression profiles across all platforms.

All raw datasets were preprocessed into a uniform representation format consisting of three components: (i) gene expression matrix $X=\left\{x_{1},\text{…},x_{n}\right\}\in R^{n\times m}$, where n is the number of spots (or cells) and m is the number of selected genes; (ii) spatial coordinates $S=\left\{s_{1},\text{…},s_{n}\right\}\in R^{n\times 2}$; and (iii) histological image patches $P=\{p_{1},\text{…},p_{n}\}$. For Visium data, we extracted square patches of size 16×16 pixels centered on each spot; for CosMx data, patches of size 60×60 pixels were used; and for MERFISH data, patches of size 40×40 pixels were selected to accommodate higher spatial resolution. Gene expression matrices were normalized using log‐transformation and highly variable genes were selected following the Scanpy pipeline.

As an unsupervised framework, S3RL aims to learn latent representations $Z=\left\{z_{1},\text{…},z_{n}\right\}\in R^{n\times d}$ of spots and simultaneously assign labels $l=\{l_{1},\text{…},l_{n}\}$, where d is the latent space dimension and c is the expected number of cell types or spatial domains. A spatial graph is constructed as G=(V,E), where nodes V represent spots and edges E capture neighborhood relationships. The adjacency matrix $A\in R^{n\times n}$ encodes connectivity, with $a_{ij}=1$ if (i,j)∈E and $a_{ij}=0$ otherwise. This unified preprocessing approach ensures S3RL's compatibility across datasets with heterogeneous resolutions and modalities, enabling seamless integration and comparative analysis of diverse spatial transcriptomics platforms.

### 4.2 Semantic Information Extraction Module

This module aims to extract high‐level semantic information from histological images. To this end, we employ the SimCLR framework to learn the visual features of spots by maximizing the agreement between various augmented views of the same spot in a contrastive learning manner. For the i‐th spot with image $p_{i}$, two augmented views $p_{i}^{\left(1\right)}$ and $p_{i}^{\left(2\right)}$ are regarded as a positive pair, and the augmented views of different images are regarded as negative pairs. Then we adopt the well‐trained ResNet‐50 network followed by simply Multilayer Perceptron (MLP) as the encoder f(·) to extract the visual features $h_{i}^{\left(1\right)}=f\left(p_{i}^{\left(1\right)}\right)\in R^{256}$ and $h_{i}^{\left(2\right)}=f\left(p_{i}^{\left(2\right)}\right)\in R^{256}$. Contrastive learning aims to maximize the similarity between positive pairs and minimize the similarity between negative pairs, which can be formulated as the following loss function:

(1) $$\begin{array}{cc} & L_{cons}=-\log\left(\right.\\ & \left.\frac{\exp(\mathrm{sim}\left(h_{i}^{\left(1\right)},h_{i}^{\left(2\right)}\right)/\tau )}{\sum_{j\neq i}\exp\left(\mathrm{sim}\left(h_{i}^{\left(1\right)},h_{j}^{\left(1\right)}\right)/\tau \right)+\exp\left(\mathrm{sim}\left(h_{i}^{\left(1\right)},h_{j}^{\left(2\right)}\right)/\tau \right)}\right) \end{array}$$

where sim(·,·) represents the cosine similarity between two features and τ is the temperature parameter. In this work, the random augmentations include random cropping, random flipping, and random color distortion. The learning rate is set to 0.005, τ is set to 0.5, the weight decay is set to $1e^{-6}$, and the model is trained for 500 epochs with the Adam optimizer and the batch size of 256. After training, we can obtain the visual features $H=\left\{h_{1},\text{…},h_{n}\right\}\in R^{n\times 256}$ of n spots, encoding the high‐level semantic information of histological images.

### 4.3 Signed Edge Extraction based Graph Construction Module

This module first converts the spatial location S into an undirected graph G=(V,E). Each spot in the graph G connects to their k nearest neighbors such that $E=\{\left(i,j\right)|j\in N_{i}\}$, where $N_{i}$ denotes the set of k nearest neighbors of the i‐th spot. Note that the neighboring nodes $N_{i}$ can be determined by different distance or similarity metrics, and we adopt the commonest Euclidean distance in this work. Besides, this module further constructs a signed similarity graph based on the visual features to extract reliable pairwise relations (i.e. Signed Edges) between spots. Specifically, each spot is linked to another spot with a positive edge if they have the highest similarity and a negative edge if they have the lowest similarity based on the visual features. We denote the positive and negative edges by $E_{p}^{+}$ and $E_{p}^{-}$, respectively. Similarly, we extract the prior knowledge inherent in gene profiles X. We calculate the pairwise similarity between spots based on the expression profiles X and retain the positive and negative edges according to the highest and lowest similarity, which are represented by $E_{g}^{+}$ and $E_{g}^{-}$, respectively.

The spatial graph G presents local spatial information where spatially neighboring spots are more likely of the same cell type, but at the same time, it is incapable of capturing the global structure and high‐level semantic information. Thus we introduce the positive and negative edges from the visual features and gene expression profiles to enhance the spatial graph. The positive edges indicate the possible homogeneity between spots, while the negative edges indicate the possible heterogeneity. We integrate the positive edges into the spatial graph such that the semantic graph $G^{\prime }=\left(V,E^{\prime }\right)$ and $E^{\prime }=E\cup E_{p}^{+}\cup E_{g}^{+}$. In this way, the graph $G^{\prime }$ contains the spatial information and the semantic information from histological images and gene expression profiles, which facilitates the identification of distinct cell types and separating heterogeneous domains. Besides, the negative graph $G^{-}=\left(V,E^{-}\right)$, where $E^{-}=E_{p}^{-}\cup E_{g}^{-}$, indicates dissimilarity of spots and is used to push them apart in the embedding space, for which we will explain more detailed in the following section.

### 4.4 Separable Representation Learning

S3RL employs the popular GNNs as the encoder and decoder to learn informative low‐dimension representations of spots by collaboratively taking into account the spatial information, the high‐level semantic information, and the gene expression profiles. The encoder $f_{\theta_{e}}\left(\cdot \right)$ parameterized by $\theta_{e}$ takes the enhanced semantic graph $G^{\prime }$ and the gene expression profiles X as input and outputs the low‐dimension representation Z, which is followed by the symmetrical decoder $f_{\theta_{d}}\left(\cdot \right)$ parameterized by $\theta_{d}$ to reconstruct the gene expression profiles. The encoder $f_{\theta_{e}}\left(\cdot \right)$ and decoder $f_{\theta_{d}}\left(\cdot \right)$ both consist of two‐layer Graph Transformer Networks (GTNs) [74]. Formally, the (l+1)‐th layer of the encoder is formulated as:

(2) $$\begin{array}{c} z_{i}^{\left(l+1\right)}=\sigma \left(\beta_{i}^{\left(l\right)}W_{1}^{\left(l\right)}z_{i}^{\left(l\right)}+\left(1-\beta_{i}^{\left(l\right)}\right)\sum_{j\in N_{i}}\alpha_{ij}W_{2}^{\left(l\right)}z_{j}^{\left(l\right)}\right) \end{array}$$

in which $z_{i}^{\left(l\right)}$ is the representation of the i‐th spot in the l‐th layer, $W_{1}^{\left(l\right)}$ and $W_{2}^{\left(l\right)}$ are the learnable transformation matrix. σ(·) is the activation function and S3RL adopts the ELU function. $\alpha_{ij}$ is the attention weight calculated by the scaled dot‐product attention mechanism, and $\beta_{i}^{\left(l\right)}$ is a gated residual connection that balances the importance between neighboring nodes' features and the i‐th node's own feature.

The symmetrical decoder aims to reconstruct the gene expression profiles in the same way as depicted in Equation (2). Supposing the reconstructed profiles are $\tilde{X}=f_{\theta_{d}}\left(Z\right)$, S3RL employs the Consine Error (CE) loss to measure the difference between the input and the reconstructed profiles as follows:

(3) $$\begin{array}{c} L_{rec}=\frac{1}{n}\sum_{i=1}^{n}\left(1-\frac{x_{i}^{⊤}{\tilde{x}}_{i}}{∥x_{i}{∥}_{2}{∥{\tilde{x}}_{i}∥}_{2}}\right) \end{array}$$

where ${∥\cdot ∥}_{2}$ denotes the $\ell_{2}$‐norm of vectors.

Besides, the learned representation Z should maintain the topological structure of graphs $G^{\prime }$ and $G^{-}$, such that the spatial and semantic information is maximally preserved. S3RL introduces the next loss function to achieve it:

(4) $$\begin{array}{c} L_{topo}=\sum_{\left(i,j\right)\in E^{\prime }}-\log\left(\sigma \left(z_{i}^{⊤}z_{j}\right)\right)-\sum_{\left(i,j\right)\in E^{-}}\log\left(1-\sigma \left(z_{i}^{⊤}z_{j}\right)\right) \end{array}$$

Minimizing $L_{topo}$ encourages spatially neighboring spots and semantically similar spots to be close in the embedding space while pushing dissimilar spots apart. In the training stage, S3RL randomly masks partial spots and replaces their profiles with a shard and learnable token to avoid overfitting and make the learned representation more expressive.

With the learned representations, one can apply a variety of off‐the‐shelf clustering algorithms to identify the cell type of each spot, such as k‐means [75], Leiden [76], and Mclust [77], which are commonly used in many spatial transcriptomics studies [15, 19, 20]. However, this two‐stage learning fashion, in which representation learning and clustering are performed separately, will lead to error propagation and information loss, resulting in suboptimal performance. Thus, S3RL proposes the hyperspherical prototype learning submodule to obtain the clustering results in an end‐to‐end manner.

S3RL first defines c prototypes $\left\{p_{1},p_{2},\text{…},p_{c}\right\}$ on the d‐dimension unit‐hypersphere space such that the pairwise distance between any two prototypes is as large as possible. More importantly, each prototype is regarded as a centroid of a cell type and they are evenly scattered in the unit‐hypersphere space. It can be achieved by minimizing the following problem using the gradient descent algorithm:

(5) $$\begin{array}{c} \min\max_{i\neq j}\;p_{i}^{⊤}p_{j} \end{array}$$

Then, S3RL maps the representation $z_{i}$ of the i‐th spot into the same unit‐hypersphere space as:

(6) $$\begin{array}{c} {\tilde{z}}_{i}=\frac{z_{i}}{∥z_{i}{∥}_{2}}R \end{array}$$

where $R\in R^{d\times d}$ is a learnable orthogonal rotation matrix to align representations with c prototypes. In this way, the cell type affinity of the i‐th spot with respect to the j‐th prototypes can be calculated as:

(7) $$\begin{array}{c} y_{ij}=\frac{\exp({\tilde{z}}_{i}^{⊤}p_{j})}{\sum_{k=1}^{c}\;\exp\left({\tilde{z}}_{i}^{⊤}p_{k}\right)} \end{array}$$

S3RL can obtain the final clustering label by assigning the i‐th spot to the prototypes with the largest similarity as:

(8) $$\begin{array}{c} l_{i}=\underset{j}{\arg\max}\;y_{ij} \end{array}$$

S3RL further introduces a hyperspherical regularization to promote the separability of the learned representations:

(9) $$\begin{array}{c} L_{clu}=\frac{1}{\sqrt{c}-1}\left(\sqrt{c}-\frac{1}{\sqrt{n}}\sum_{j=1}^{c}{∥y_{j}∥}_{2}\right) \end{array}$$

$L_{clu}$ encourages each spot to associate with a single prototype with higher confidence, while promoting separation between different types of spots, which improves the final clustering performance. Moreover, it ensures an even distribution of spots across prototypes, preventing empty clusters.

Instead of keeping the prototypes unchanged, prototypes are dynamically updated to adapt to diverse histology. The ideal situation derived by minimizing $L_{clu}$ is that each prototype is crowded with $\left\lfloor \frac{n}{c}\right\rfloor$ spots, i.e., for j‐th prototype, the vector $y_{j}^{*}$ has $\left\lfloor \frac{n}{c}\right\rfloor$ ones and $n-\lfloor \frac{n}{c}\rfloor$ zeros. Then, S3RL can measure the difference between the current clustering results with the ideal one as follows:

(10) $$\begin{array}{c} d_{j}=t*\mathrm{KL}\left(\frac{y_{j}}{∥y_{j}{∥}_{1}},\frac{y_{j}^{*}}{∥y_{j}^{*}{∥}_{1}}\right) \end{array}$$

where KL(·) denotes the Kullback–Leibler divergence and it can be replaced by other metrics, such as Spearman correlation, Jensen–Shannon divergence, and so on. t is a hyperparameter to adjust the scale of $d_{j}$, and ${∥\cdot ∥}_{1}$ denotes the $\ell_{1}$‐norm of vectors. The coefficient $d_{j}$ reflects the degree of spots assigned to the j‐th prototype deviating from the ideal one. Thus, S3RL can update the j‐th prototype to minimize the deviation as:

(11) $$\begin{array}{c} p_{j}\leftarrow p_{j}+{\tilde{p}}_{j} \end{array}$$

where ${\tilde{p}}_{j}$ is sampled from the guassian distribution $N(0,d_{j}I)$ with the mean of 0 and the variance of $d_{j}I$. S3RL employs the reparameterization trick maintain the differentiability of the prototype update process.

The overall loss function of this separable representation learning module consists of the reconstruction loss, the topological loss, and the clustering loss:

(12) $$\begin{array}{c} L=L_{rec}+\lambda_{1}L_{topo}+\lambda_{2}L_{clu} \end{array}$$

where $\lambda_{1}$ and $\lambda_{2}$ are hyperparameters to balance the importance of different losses. The model is trained end‐to‐end by minimizing the loss function L using the Adam optimizer, for at most 2000 epochs.

### 4.5 Multi‐Slice Alignment and Joint Training

In multi‐slice spatial transcriptomics analysis, balancing clustering performance with alignment and data integration remains a crucial challenge. While S3RL demonstrates outstanding performance in cell clustering, there exists an inherent trade‐off between achieving optimal alignment across slices and preserving robust clustering results. To mitigate this conflict, we employ a spatial translation‐only alignment strategy, avoiding distortions that may artificially enhance alignment at the cost of reduced clustering performance.

Specifically, we leverage PASTE2, a state‐of‐the‐art partial alignment method, to perform global and local multi‐slice alignment. PASTE2 optimally registers **only the overlapping regions between slices using a weighted Gromo–Wasserstein optimal transport strategy, thereby reducing interference from non‐overlapping areas and enhancing robustness. We adopt the default parameter settings and use the partial_pairwise_align function to align the slices. For complete slices and partially cropped slices, we set the *s* parameter to 1 and 0.7, respectively, ensuring that the alignment remains effective under different levels of tissue overlap.

Once all slices are aligned to a common reference coordinate system, we merge them into a unified dataset for joint representation learning with S3RL. This enables similar spots to leverage neighboring spot expression patterns more effectively, ensuring both spatial coherence and feature consistency. As a result, our approach not only enhances the integration of multiple spatial slices but also maintains superior clustering accuracy, setting a robust foundation for downstream spatial transcriptomics analysis.

### 4.6 Ligand‐Receptor Pair Construction and Evaluation Metrics

To evaluate the effectiveness of different enhancement methods in ligand‐receptor (L‐R) identification, we first constructed a reliable L‐R interaction set by integrating data from the IUPHAR/BPS Guide to Pharmacology [41] and the SiGra‐aggregated database [21]. After redundancy removal, we curated 2864 unique L‐R pairs, among which 480 (189 ligands and 159 receptors) were present in the NanoString CosMx dataset. Using CellChat, we calculated the significance of each L‐R interaction across cell clusters based on $-\log_{10}\left(\text{FDR}\right)$ and visualized the results in a scatter plot, comparing raw and enhanced data. In the scatter plot (Figure S19 top), the x‐axis represents raw significance, and the y‐axis represents significance after enhancement. Each point corresponds to a specific L‐R pair: the top‐left quadrant represents L‐R pairs that are newly significant only after enhancement, the bottom‐right quadrant shows those that lost significance after enhancement, the top‐right quadrant indicates consistently significant L‐R pairs, and the bottom‐left quadrant marks pairs that remain non‐significant in both conditions.

To further quantify the model performance in L‐R identification, we defined three complementary metrics. The *Enhanced Specificity Ratio (ESR)* is defined as $\text{ESR}=\frac{N_{\text{top-left}}}{N_{\text{bottom-right}}}$, reflecting the method's ability to discover new L‐R pairs not detected in the raw data relative to those lost after enhancement. The *Effective Enhancement Percentage (EEP)* is defined as $\text{EEP}=\frac{N_{\text{top-left}}}{N_{\text{top-left}}+N_{\text{bottom-right}}}\times 100$, indicating the proportion of newly discovered significant L‐R pairs among all changed ones. The *Specificity Stability Percentage (SSP)* is computed as $\text{SSP}=\frac{N_{\text{top-right}}}{N_{\text{top-right}}+N_{\text{bottom-right}}}\times 100$, measuring how well the method preserves originally significant L‐R pairs during enhancement.

Here, $N_{\text{top-left}}$ denotes the number of L‐R pairs that become significant only after enhancement, $N_{\text{top-right}}$ refers to those consistently significant before and after enhancement, and $N_{\text{bottom-right}}$ represents L‐R pairs that were significant in raw data but lost significance post‐enhancement. Together, these metrics offer a comprehensive and interpretable assessment of a model's capacity to enhance biologically meaningful cell‐cell communication signals while minimizing disruption to existing ones.

### 4.7 Cell Type Deconvolution, Spatially Variable Gene Identification, and Pseudotime Analysis

To estimate cell type compositions across spatial transcriptomics spots, we performed deconvolution using the CARD framework(v1.1) [54] with default parameter settings. CARD integrates scRNA‐seq reference data with spatial transcriptomics to infer cell type proportions at each spatial location.

For identification of spatially variable genes (SVGs), we employed CELINA(v1.0) [52], also using default configurations. CELINA detects genes exhibiting significant spatial expression variability, serving as the foundation for downstream functional and regulatory analyses.

Pseudotime trajectory inference was performed using Monocle3(v2.30.1) [55] on the latent embeddings generated by S3RL. The denoised and biologically enriched representation of spatial data provided by S3RL enabled Monocle3 to capture coherent developmental and regulatory trajectories across tissue sections.

### 4.8 Gene Set Enrichment and Transcription Factor Analysis

To explore functional pathways and transcriptional regulation in spatial transcriptomics data, we performed a series of enrichment analyses. For the human lung cancer dataset, we applied Gene Set Variation Analysis (GSVA) using the ssgseaParam function in the GSVA package(v2.0.7) [78], with default settings adjusted to minSize = 10, maxSize = 500, and alpha = 0.25. Gene Ontology (GO) enrichment analysis was conducted using the gseGO function from the clusterProfiler package(v4.14.6) [79], focusing on Biological Process (BP) terms with a significance threshold of p‐value < 0.05.

For soybean cotyledon stage seed data, we utilized the PlantRegMap [63] platform for GO and transcription factor (TF) enrichment analysis. Enriched TFs were further cross‐referenced with the PlantTFDB database(v5.0) to evaluate enrichment fold changes. To identify TF binding motifs, we employed FIMO (Find Individual Motif Occurrences) [80] from the MEME Suite(v4.4).

For visualization of sequencing coverage and chromatin accessibility, we used the Plant Epigenome Browser, as provided by Zhang etc [61], allowing interactive exploration of genomic loci in soybean tissues.

### 4.9 Benchmarking Methods and Performance Evaluation

To evaluate the performance of our proposed model, we conducted a comprehensive comparison against several state‐of‐the‐art methods, including BayesSpace, Giotto, Seurat, SEDR, SiGra, conST, SpaceFlow, spaGCN, and STAGATE, using Adjusted Rand Index (ARI) as the evaluation metric (Figure 2). The ARI measures the alignment between predicted spatial clusters and the ground truth annotations, providing an indication of how well each method captures the underlying biological structure of the tissue samples. For a fair and consistent comparison, all baseline methods were executed using their default parameters as recommended in their original publications or official implementations.

Our model consistently outperforms the competing methods across multiple datasets, including lung cancer, mouse brain anterior, and human breast cancer etc, demonstrating superior accuracy in segmenting distinct spatial regions. Specifically, our method achieves a significantly higher ARI score, indicating that it better preserves the spatial and biological context of the tissue. The enhanced representation of gene expression and spatial structure in our model allows for more accurate delineation of complex tissue microenvironments compared to existing methods.

### 4.10 Identifying Differentially Expressed Genes and Adjacent Cell Communications

In addition to clustering performance, we also explored the identification of differentially expressed genes (DEGs) and the analysis of adjacent cell communications. For this, we used the Wilcoxon test to identify DEGs in each spatial region, comparing gene expression between neighboring cell clusters. We set a threshold of log2 fold change greater than 1 and adjusted p‐value less than 0.05 for significant DEGs.

Furthermore, we analyzed cell–cell communication by calculating the interaction strength between adjacent cell pairs. This was done by aggregating the ligand‐receptor interaction scores for neighboring cells and normalizing the interaction strengths based on the expression levels of key signaling molecules. The communication strength between adjacent cell pairs reflects the level of molecular crosstalk, which is critical in understanding the role of different cell types in the tissue microenvironment.

The results revealed several important communication pathways, including growth factor signaling, immune cell interactions, and tumor‐stroma crosstalk. Notably, enhanced data from our model provided a clearer view of these communication patterns, uncovering interactions that were less visible in the raw data. This enhanced resolution of cell‐cell communication networks highlights the biological relevance of our model and its ability to capture key molecular interactions in spatial transcriptomics datasets.

To validate the effectiveness of the enhanced count matrix, we analyzed both the original and enhanced matrices using Seurat [25] and CellChat [3]. First, we employed Seurat's Wilcoxon Rank Sum test function to identify marker genes, setting the significance level at 0.05. Following the identification of marker genes, we selected upregulated genes with $\log_{2}\text{FC}\geq 0.2$ and adjusted p-value≤0.05, as well as downregulated genes with $\log_{2}\text{FC}\leq -0.25$ and adjusted p-value≤0.05, then plotted a volcano plot.

Subsequently, utilizing the CellChatDB (v1) [3] database of human ligand‐receptor interactions, we modeled ligand‐receptor mediated signaling interactions based on the law of mass action. This approach enabled us to determine the communication probabilities between all cell groups for each ligand‐receptor pair. We then calculated the communication probabilities between each cell type by weighting the probabilities of the relevant ligand‐receptor pairs, and plotted ligand‐receptor heatmaps and communication strength plots between different cell types.

We then compared the ligand‐receptor networks and marker genes of the enhanced and original matrices, identifying newly emerged ligand‐receptor pathways in the enhanced matrix. These corresponding genes were highlighted in the marker genes volcano plot. Additionally, we grouped the data by cell type and used the count matrix to plot violin plots of the gene intensities for the highlighted genes in the marker genes volcano plot.

## 5 Statistics and Reproducibility

No statistical method was used to predetermine the sample size. No data were excluded from the analyses. The experiments were not randomized. The investigators were not blinded to allocation during experiments and outcome assessment.

## 6 Reporting Summary

Further information on research design is available in the Nature Portfolio Reporting Summary linked to this article.

## 7 Data Availability 

All datasets used in this study are publicly available and widely adopted as benchmarks for evaluating spatial transcriptomics methods. The 10X Visium spatial transcriptomics dataset of the human dorsolateral prefrontal cortex (DLPFC), comprising 12 tissue slices with manually annotated cortical layers, was obtained from Maynard et al. [24]. The Mouse Brain Anterior and Human Breast Cancer datasets were downloaded from the official 10X Genomics website (https://www.10xgenomics.com/resources/datasets), both sequenced using the Visium platform. The 10X Visium dataset of human lung cancer is also publicly available (https://www.10xgenomics.com/resources/datasets/human‐lung‐cancer‐ffpe‐2‐standard).

To assess the model's robustness on large‐scale pathological tissues, we utilized the Nanostring CosMx Spatial Molecular Imaging (SMI) dataset, which includes 20 formalin‐fixed, paraffin‐embedded (FFPE) lung cancer slices with expert‐labeled cell type annotations. This dataset is publicly available via the Nanostring official website (https://nanostring.com/products/cosmx‐spatial‐molecular‐imager/nsclc‐ffpe‐dataset/). We further validated S3RL on high‐resolution datasets, including the mouse olfactory bulb Stereo‐seq data and mouse hippocampus Slide‐seqV2 data from their original publications [10, 81], as well as the STARmap‐PLUS dataset of the mouse hippocampus, available at the Broad Single Cell Portal (ID SCP1375; data ID: spatial_13months‐disease‐replicate_1). For integration analysis, we used publicly available scRNA‐seq references: the human lung cancer scRNA‐seq data from CellxGene (https://cellxgene.cziscience.com/collections/edb893ee‐4066‐4128‐9aec‐5eb2b03f8287), and a mouse hippocampus scRNA‐seq dataset from DropViz (http://dropviz.org) corresponding to STARmap‐PLUS. Additionally, we incorporated a single‐nucleus RNA‐seq (snRNA‐seq) dataset from the human dorsolateral prefrontal cortex (BA9), comprising 78,886 nuclei and 30 062 genes, obtained from GEO (accession number GSE144136) [29]. Some of the data can be retrieved from CELINA work [52].

For plant datasets, all soybean spatial transcriptomics data generated in this study have been deposited in GEO under accession number GSE270392.

The processed data used in this study have been uploaded to Zenodo and are freely available at: https://zenodo.org/records/15222342.

## Author Contributions

L.F., D.W. and H.S. conceptualized and supervised the study, L.F., P.W., G.X., J.L., H.S. and D.W. performed model design, software implementation and data analysis. L.F., P.W, D.W and H.S. wrote the manuscript with input from all authors. All authors read and approved the final manuscript.

## Ethics Statement

This research does not involve any ethical concerns related to resource‐poor settings, nor does it present any risks or harm to the participants.

## Conflicts of Interest

The authors declare no conflicts of interest.

## Supporting information


**Supporting File**: advs73804‐sup‐0001‐SuppMat.pdf.

## Data Availability

The data that support the findings of this study are openly available in Zenodo at https://zenodo.org/records/15222342, reference number 15222341. An open‐source Python implementation of the proposed method is available at: https://github.com/AI4Bread/S3RL.
