# Supplementary material for: S3RL: Enhancing Spatial Single‐Cell Transcriptomics With Separable Representation Learning
Source: Adv Sci (Weinh). 2026 Jan 20;13(17):e16178. doi: 10.1002/advs.202516178 (PMC13042551; doi:10.1002/advs.202516178)
Supplement: Supplementary file 1 — Supporting File: advs73804‐sup‐0001‐SuppMat.pdf. [file ADVS-13-e16178-s001.pdf]

Supplementary Materials for S3RL: Enhancing Spatial Single-cell Transcriptomics with Separable Representation Learning

Laiyi Fu[1,4,5]⋆, Penglei Wang[2] ⋆, Gaoyuan Xu[1] ⋆, Jitao Lu[1], Qinke Peng[1], Danyang Wu[1] ⋆⋆, and Hequan Sun[3] ⋆⋆

[1] Systems Engineering Institute, School of Electronic and Information Engineering, Xi'an Jiaotong University, Xi'an, Shannxi, 710049, China
[2] School of Software Engineering, South China University of Technology, Guangzhou, Guangdong, 510641, China
[3] College of Information Engineering, Northwest A&F University, Xianyang, Shannxi, 712100, China
[4] Research Institute of Xi'an Jiaotong University, Zhejiang, Hangzhou, Zhejiang, 311200, China
[5] Sichuan Digital Economy Industry Development Research Institute, Chengdu, Sichuan, 610036 , China

⋆ Equal contributions
⋆⋆ To whom correspondence should be addressed

Table of Contents

1 Correlation Analysis Between TCGA Lung Cancer Bulk RNA-seq and Nanostring ST Data . . . 4
2 S3RL Enhances Spatial Gene Expression and Cell-Cell Communication in Brain and Lung Tissues . . . 5
2.1 Differential Gene Expression and Cell-Cell Communication in DLPFC Slice 151509 . . . 5
2.2 Differential Expression and Cell-Cell Communication in Nanostring Lung Cancer Slice 003 . . . 6
3 Latent Space Clustering Results on DPFLC dataset . . . 8
4 Paired t-Test Analysis of S3RL Versus Baseline Methods . . . 10
5 Biological Interpretability of Histological Semantic Features . . . 12
6 Latent Space Clustering Results on NanoString dataset . . . 17
7 Spatial Clustering Results on Mouse Brain Anterior and Human Breast Cancer Datasets . . . 26
8 Detailed Ablation Study and Module Contributions . . . 36
8.1 Definition of Ablation Variants . . . 36
8.2 Visual Semantics as a Spatial Denoiser . . . 37
8.3 Dynamic Prototype Learning Enhances Latent Separability . . . 37
9 Supplementary References . . . 39

List of Figures

S1 S3RL Clustering Results on 10X Visium dataset . . . 8
S2 Comparison of Clustering Results on 10X Visium DLPFC Dataset . . . 9
S3 Statistical significance of S3RL performance improvements. . . . 10
S4 Raw and enhanced clustering performance in DLFPC data. . . . 11
S5 Cell Trajectory Analysis on 10X Visium DLPFC Dataset . . . 12
S6 Biological interpretability of S3RL's visual features on DLPFC and Nanostring datasets. . . . 14
S7 Cell Type Deconvolution Analysis on 10X Visium DLPFC Dataset . . . 15
S8 Cell Type Deconvolution Results on different methods . . . 16
S9 S3RL Clustering Results on NanoString dataset . . . 17
S10 Comparison of clustering results across 20 Nanostring lung cancer slices using different methods. . . . 18
S11 Clustering visualization. . . . 18
S12 Marker gene reconstruction performance across different methods . . . 19
S13 Latent space clustering performance in NanoString data. . . . 20
S14 Comparison of marker gene expression in raw and enhanced NanoString data. . . . 20

S15 Correlation analysis between bulk RNA-seq and raw/enhanced spatial transcriptomics data across 20 Nanostring lung slices of different methods. . . . 21
S16 Correlation analysis between bulk RNA-seq and raw spatial transcriptomics data. . . . 22
S17 Correlation analysis between bulk RNA-seq and enhanced spatial transcriptomics data. . . . 23
S18 Comparison of reconstruction-based methods on Nanostring lung cancer slice 001. . . . 24
S19 Comparison of ligand-receptor (L-R) pair significance across different enhancement methods. . . . 25
S20 Clustering comparison on Mouse Brain Anterior and Human Breast Cancer datasets. . . . 26
S21 Gene expression reconstructions by baseline models in Mouse Brain Anterior dataset. . . . 27
S22 Marker gene reconstruction across methods in human breast cancer tissue. . . . 28
S23 GSEA enrichment plots of biological processes in lung cancer. . . . 29
S24 Pseudotime inference in S3RL latent space. . . . 30
S25 Spatial distribution and gene expression in raw data. . . . 30
S26 Spatial gene expression in raw mouse hippocampal data. . . . 31
S27 Spatial gene expression in S3RL-reconstructed mouse hippocampal data. . . . 32
S28 Gene co-expression networks and spatial correlations in microglia and interneurons. . . . 33
S29 Metagene-specific GO enrichment analysis in soybean vascular parenchyma cells. . . . 34
S30 Transcription factor enrichment analysis in soybean vascular parenchyma cells. . . . 35
S31 Predicted transcription factor (TF) binding site motifs for soybean SC vascular parenchyma regulatory network. . . . 36
S32 Qualitative comparison of spatial clustering across ablation variants. . . . 37
S33 UMAP visualization of latent embeddings across ablation variants. . . . 38

List of Tables

S1 Qualitative comparison of spatial clustering across all comparisons of DLPFC and Nanostring Datasets. . . . 38

1 Correlation Analysis Between TCGA Lung Cancer Bulk RNA-seq and Nanostring ST Data

We evaluated the correlation between TCGA bulk RNA-seq data (TCGA-LUAD) and single-cell spatial transcriptomics (ST) data from the TCGA database and Nanostring dataset to assess our model's effectiveness in capturing biologically relevant expression patterns. Pearson correlation coefficients were calculated for each slice, comparing raw and enhanced ST data against bulk RNA-seq profiles. As shown in Fig. 3H, the enhanced data yields higher correlation values in 14 out of 20 slices(over 70%), demonstrating that S3RL more effectively aligns spatial transcriptomic signals with bulk tissue-level expression patterns.

To further validate this finding, we compared our method with four representative spatial transcriptomics methods that include data reconstruction capabilities—GraphST, SEDR, SiGra, and STAGATE. Supplementary Fig. S12 presents the correlation bar plots for each method across all 20 slices, comparing their reconstructed outputs (enhanced) and raw ST data. Notably, our S3RL-enhanced data consistently achieves higher correlation values than the corresponding raw data in nearly all slices. In contrast, SEDR and SiGra show little to no improvement, while STAGATE exhibits modest gains.

Interestingly, GraphST displays a unique pattern: its reconstructed data yields higher correlation values than raw data across all slices. However, as shown in Supplementary Fig. S9, the reconstructed spatial expression of marker genes by GraphST appears overly smoothed and lacks distinct expression boundaries. We hypothesize that the inflated correlation values may result from this oversmoothing effect, where uniform expression values lead to artificially high similarity with bulk profiles despite reduced biological interpretability. In contrast, S3RL maintains sharper gene-specific expression patterns while simultaneously improving alignment with bulk RNA-seq data, underscoring its strength in balancing biological resolution and statistical correlation.

To further investigate this trend, we analyzed the scatter plots of correlation values between bulk RNA-seq and spatial transcriptomics for each field of view (FOV), as shown in Supplementary Fig. S13 and Fig. S14. While the raw ST data exhibits a more concentrated distribution along the diagonal, its overall correlation with bulk RNA-seq is lower. This suggests that although some genes in the raw data match bulk RNA-seq values more closely, the overall expression patterns remain suboptimal due to noise and data sparsity. In contrast, the enhanced ST data demonstrates a higher correlation coefficient, signifying a more consistent alignment with bulk RNA-seq expression trends. However, the scatter plots reveal that enhanced data points exhibit slightly increased dispersion, indicating that while the global consistency with bulk RNA-seq improves, local variability remains, likely due to the non-linear transformation applied during enhancement.

These findings further validate that our model enhances the consistency between bulk RNA-seq and single-cell ST data, thereby bridging the gap between bulk and single-cell perspectives. The enhancement process improves global gene

expression alignment while maintaining meaningful biological variability, ultimately providing a more comprehensive and biologically interpretable representation of the tumor microenvironment in lung cancer tissues.

2 S3RL Enhances Spatial Gene Expression and Cell-Cell Communication in Brain and Lung Tissues

2.1 Differential Gene Expression and Cell-Cell Communication in DLPFC Slice 151509

To further explore whether S3RL's data representation and model reconstruction could more accurately capture and recover valuable signals and hidden biological meanings that are often overlooked due to sparse and noisy single-cell data, we chose to observe two distinct datasets in the following two subsections. Specifically, we selected slices from two datasets and analyzed their gene expression and cell-cell communication patterns both in the raw data and in the S3RL-enhanced reconstructed data. In the case of the DLPFC 151509 slice, we focused on differential gene expression across cortical layers and evaluated the effectiveness of S3RL-based data enhancement in improving spatial transcriptomic resolution.

As shown in Figure 4A, marker genes were identified across different cortical layers, and volcano plots were used to compare their expression between raw data and S3RL-reconstructed data. The enhanced data exhibited a stronger layer-specific expression pattern for key genes such as FGFR1, FGFR2, NOTCH4, and DLL4. For instance, FGFR1/FGFR2 were significantly expressed in Layer 1, Layer 5, and Layer 6, whereas DLL4/NOTCH4 showed a pronounced preference for Layer 3 and Layer 5. These results demonstrate that S3RL effectively refines biologically meaningful gene expression patterns, offering an improved representation of spatially resolved transcriptional activity.

To further investigate the impact of S3RL enhancement, violin plots were generated to visualize expression distributions of these key genes before and after reconstruction. In raw data, some genes exhibited diffuse or ambiguous distribution across layers, making it difficult to delineate layer-specific patterns. However, in S3RL-enhanced data, genes such as FGFR1, FGFR2, and NOTCH4 displayed sharper layer distinctions, demonstrating S3RL's ability to enhance spatial resolution. We further employed CellChat[1] to infer ligand-receptor interactions between cortical layers. The heatmap illustrates the intensity of ligand-receptor interactions, showing increased expression levels for key receptor-ligand pairs such as DLL4-NOTCH4 and FGF-FGFR axes in the enhanced data (Figure 4B). Additionally, network diagrams highlight inter-layer communication patterns, where S3RL-enhanced data reveal more pronounced inter-layer signaling, particularly between Layer 3, Layer 5, and white matter (WM), further validating S3RL's capacity to reconstruct intercellular communication networks with greater accuracy.

Building upon these findings, we examined two major signaling pathways in cortical organization. FGF8/FGF16 - FGFR1/FGFR2 (Fibroblast Growth Factor Pathway) is critical for GnRH neuron maturation and plays a key role in

tumor cell survival[2–4]. In S3RL-enhanced data, FGFR1 and FGFR2 showed stronger expression in Layer 1, Layer 5, and Layer 6, indicating S3RL's ability to refine FGF signaling representation in cortical layers. Similarly, DLL4-NOTCH4 signaling is essential for cerebral vascularization and blood-brain barrier maintenance, and it plays a crucial role in glioblastoma angiogenesis[5, 6]. In S3RL-enhanced data, DLL4 and NOTCH4 exhibit clearer expression in Layer 3 and Layer 5, confirming S3RL's improved reconstruction of Notch-mediated vascular formation. These results collectively demonstrate S3RL's effectiveness in refining cortical layer-specific gene expression and cell-cell communication networks, providing a powerful framework for enhancing spatial transcriptomics in complex brain tissues.

2.2 Differential Expression and Cell-Cell Communication in Nanostring Lung Cancer Slice 003

To investigate tumor-specific transcriptional patterns and intercellular signaling in lung tissue, we further applied S3RL enhancement to the Nanostring slice 003. This allowed for a more precise identification of differentially expressed genes across spatial clusters and a clearer characterization of cell-cell communication events within the tumor microenvironment. As shown in Figure **??**C, volcano plots compare gene expression patterns between raw and S3RL-reconstructed data. The enhanced data exhibits stronger cluster-specific expression for key genes, such as INSR, FGFR1, EPHB4, and CSF3R, with INSR enriched in endothelial cells, FGFR1 in macrophages (Mcell), and EPHB4 in tumor cells. These findings suggest that S3RL effectively sharpens the spatial resolution of key gene expressions, enhancing the biological interpretability of the data.

To further explore gene expression distribution, violin plots were generated to illustrate the spatial localization of FGFR1, INSR, CSF3R, and EPHB4 across different clusters. Compared to raw data, the S3RL-enhanced dataset reveals clearer expression patterns, including increased FGFR1 expression in macrophages, INSR enrichment in tumor and endothelial cells, and more pronounced EPHB4 expression in tumor cells. Additionally, we employed CellChat[1] to construct cell-cell communication networks and used heatmaps to quantify ligand-receptor interactions between clusters (Figure **??**D). The enhanced data reveals a significant increase in biologically meaningful intercellular interactions, particularly in the INS-INSR, FGF-FGFR, and EFNB-EPHB signaling pathways. Network diagrams further illustrate strengthened communication between tumor cells, endothelial cells, and immune cells (mast, lymphocyte, and neutrophil). Notably, fibroblasts also show increased interactions with neutrophils, mast cells, and lymphocytes, suggesting an enhanced immune regulatory role within the tumor microenvironment.

Building upon these findings, we further investigated the oncogenic mechanisms of the INS-INSR, FGF2-FGFR1, and EFNB1/EFNB3-EPHB4 signaling pathways. The INS-INSR axis is overexpressed in endothelial cells and serves as a hallmark of tumor vascularization[7]. The S3RL-enhanced data confirms a significant upregulation of INSR in tumor and endothelial cells, highlighting its role

in tumor angiogenesis and tumor-endothelial interactions. The FGF2-FGFR1 axis, a key fibroblast growth factor signaling pathway, is known to drive cancer cell proliferation, angiogenesis, and metastasis[3, 8]. In the enhanced dataset, FGFR1 expression is markedly increased in macrophages and tumor cells, consistent with FGF-FGFR signaling aberrations observed in NSCLC, breast cancer, and gastrointestinal cancers[9]. Furthermore, the EFNB1/EFNB3-EPHB4 axis, a receptor tyrosine kinase (RTK) pathway, plays a crucial role in promoting tumor survival, migration, and vascularization[10, 11].In the S3RL-enhanced data, EPHB4 expression is significantly upregulated in tumor cells (violin plot in Fig.4B), consistent with a 3-fold increase in lung cancer tissues compared to normal tissues [12], and our data show enhanced interaction with immune cells, particularly mast and lymphocyte clusters. These results demonstrate that the S3RL method improves the resolution of key oncogenic signaling pathways, enhancing the biological relevance of spatial transcriptomics in tumor microenvironment studies.

3 Latent Space Clustering Results on DPFLC dataset

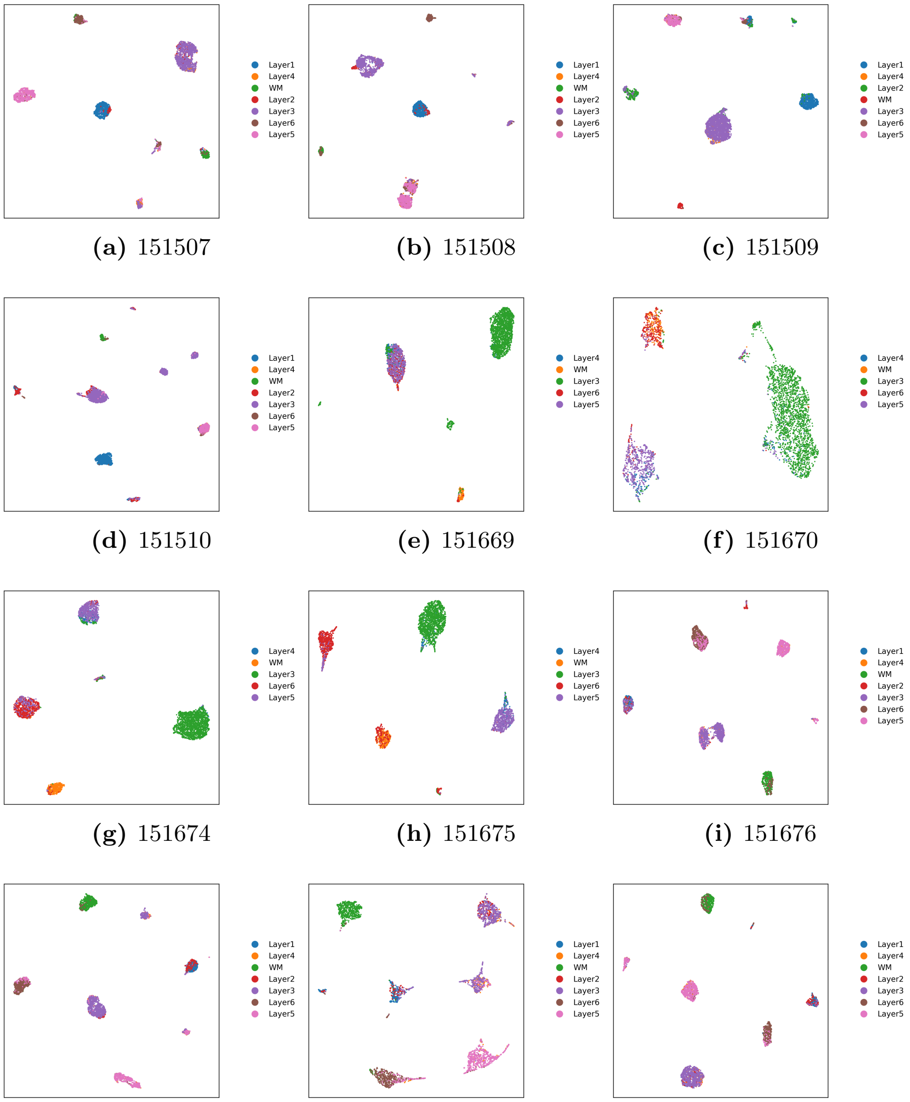

(a) 151507 **(b)** 151508 **(c)** 151509

(d) 151510 **(e)** 151669 **(f)** 151670

(g) 151674 **(h)** 151675 **(i)** 151676

(j) 151674 **(k)** 151675 **(l)** 151676

Fig. S1: Clustering results in the latent space for 12 DLPFC slices from the 10X Visium dataset using S3RL. Each subplot represents the latent space embedding of one slice, where spots are colored according to their predicted clusters.

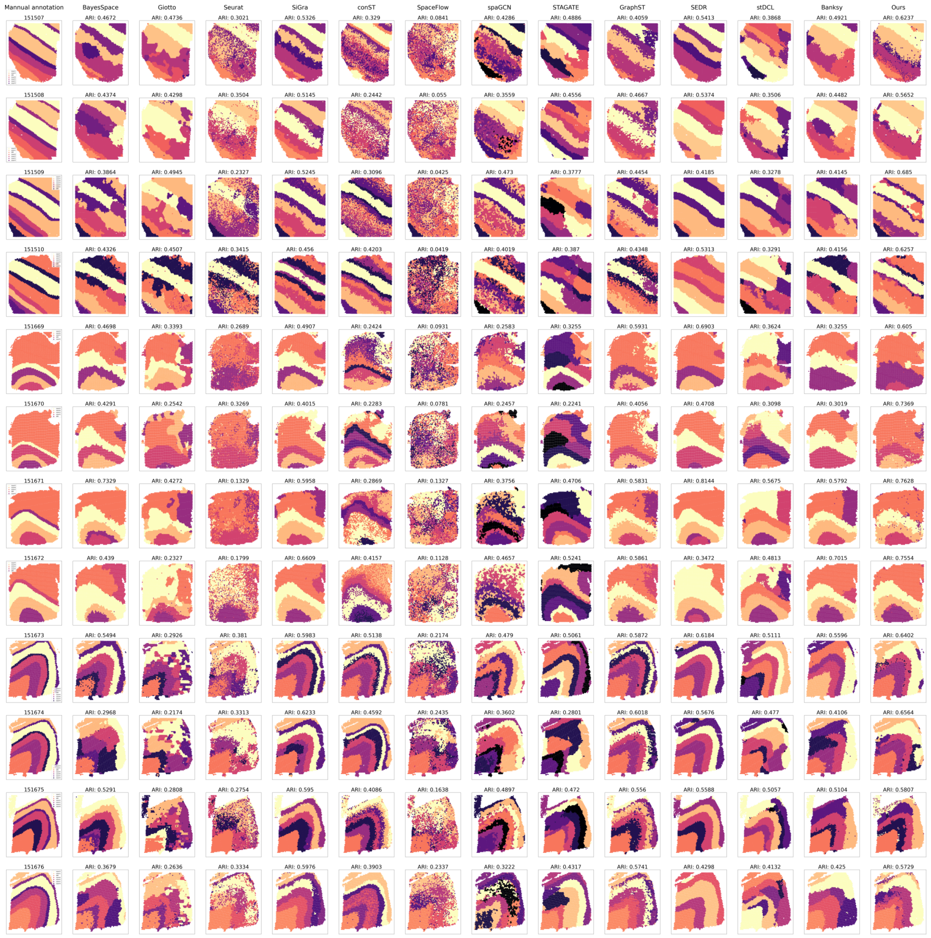

Fig. S2: Visualization of clustering results across 12 DLPFC slices from the 10X Visium dataset. The first column presents manual annotations as ground truth, while the remaining columns show clustering results obtained from various spatial transcriptomics analysis methods, including BayesSpace, Giotto, Seurat, SiGra, conST, SpaceFlow, spaGCN, STAGATE, GraphST, SEDR, stDCL, Bansky, and our proposed method. Each subplot represents a different tissue slice, with spots colored according to their predicted clusters. The Adjusted Rand Index (ARI) values are displayed for each method, indicating clustering performance relative to ground truth annotations.

4 Paired t-Test Analysis of S3RL Versus Baseline Methods

To rigorously assess the statistical robustness of these gains, we conducted paired t-tests across all slices of the DLPFC (12 slices) and Nanostring (20 slices) datasets. As visualized in Fig. S3, S3RL yields uniformly positive T-values and significantly low P-values (often $p \ll 0.05$), rejecting the null hypothesis that its performance advantages arise from random variation. The consistently positive T-values further indicate that S3RL's mean performance is systematically higher than that of all competing methods.

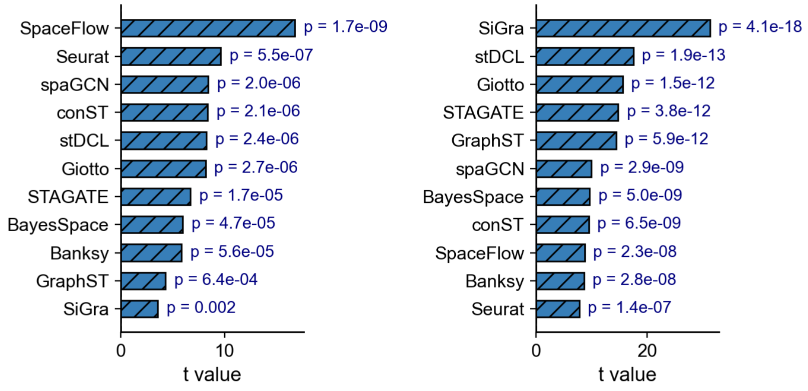

(a) DLPFC **(b)** Nanostring

Fig. S3: This plot visualizes the T-values and P-values derived from paired t-tests between S3RL and baseline methods on (a) DLPFC and (b) Nanostring datasets. S3RL demonstrates statistically significant superiority across diverse tissue architectures.

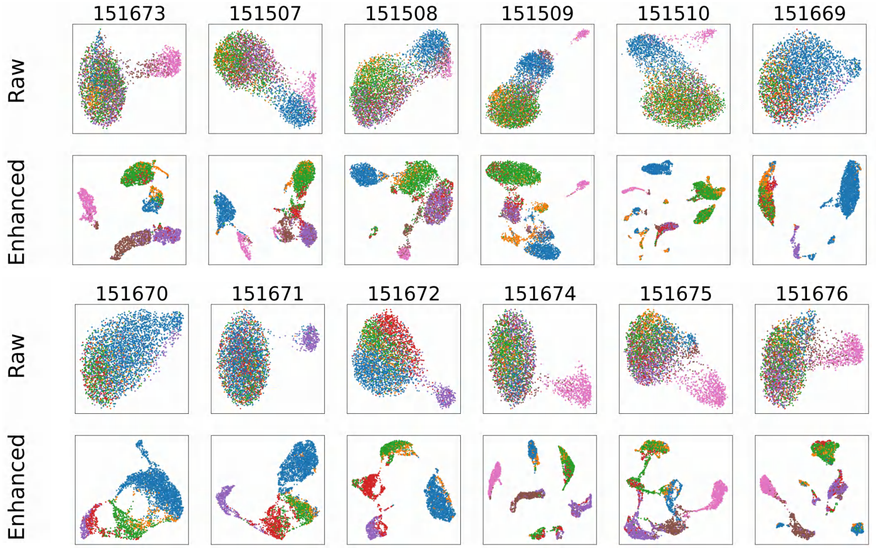

Fig. S4: Raw and enhanced clustering performance visualization in the latent space of DLPFC slices. The first and third rows represent the raw data, and the second and fourth rows correspond to the S3RL framework-enhanced data. The S3RL enhanced embeddings show exceptionally elevated separation and compactness compared to the raw embeddings, facilitating the identification of cellular heterogeneity within the DLPFC tissue architecture.

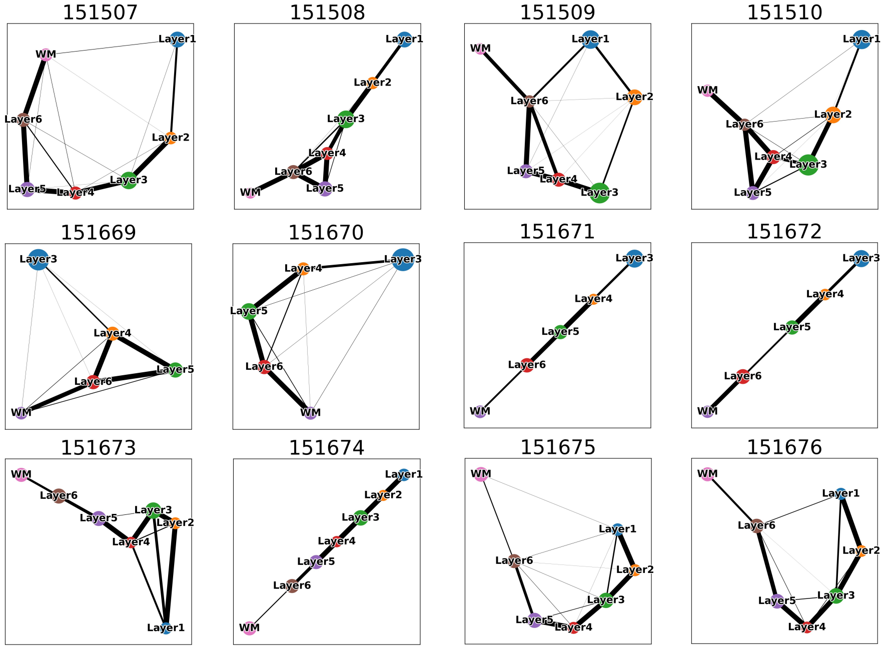

Fig. S5: Cell trajectory analysis on 12 slices of the 10X Visium DLPFC dataset using S3RL. Each subplot represents a different tissue slice, where layers (e.g., Layer1–Layer6 and WM) are connected based on inferred cellular transitions. The thickness of the edges indicates the strength of inferred connections, reflecting potential spatial interactions and lineage relationships among layers. This visualization demonstrates the ability of S3RL to preserve biologically meaningful spatial connectivity while distinguishing distinct cellular compartments.

5 Biological Interpretability of Histological Semantic Features

To validate that the high-level semantic information extracted from histological images by S3RL is biologically meaningful rather than a "black box," we conducted an interpretability analysis on the DLPFC dataset. Specifically, we extracted the latent visual embeddings (H) generated by the contrastive learning module *prior* to any integration with gene expression data. We then applied Leiden clustering solely to these visual features to assess their spatial coherence independent of transcriptomic signals. As illustrated in Fig. S6, the resulting visual-only clusters reveal substantial correlation with ground truth anatomical structures:

1. **Laminar Organization:** The visual feature clusters approximate the broad laminar organization of the human DLPFC (Layers 1–6). These transitions are consistent with classical cytoarchitectonic distinctions, such as gradients in cell density and variations in soma size, which are captured by the S3RL visual encoder.
2. **White Matter vs. Gray Matter:** The visual embeddings clearly distinguish white matter (WM) from gray matter (GM), reflecting established histological contrasts (e.g., the density of myelinated axons versus neuronal somata).

While visual features alone yield coarser boundaries compared to the full multi-modal S3RL framework, this analysis confirms that the visual module captures interpretable cytoarchitectural patterns that contribute meaningfully to the final spatial reconstruction.

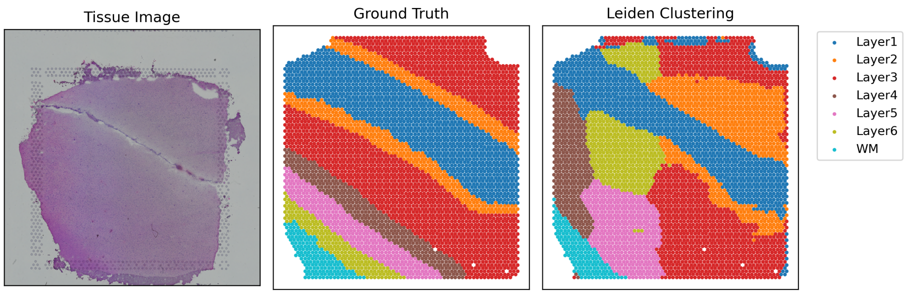

(a) Slice 151509 of DLPFC

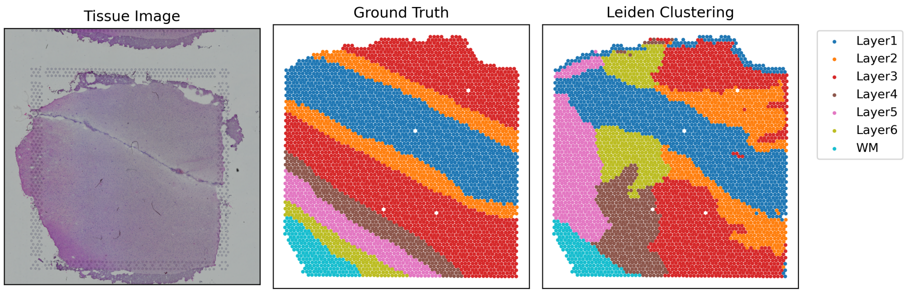

(b) Slice 151510 of DLPFC

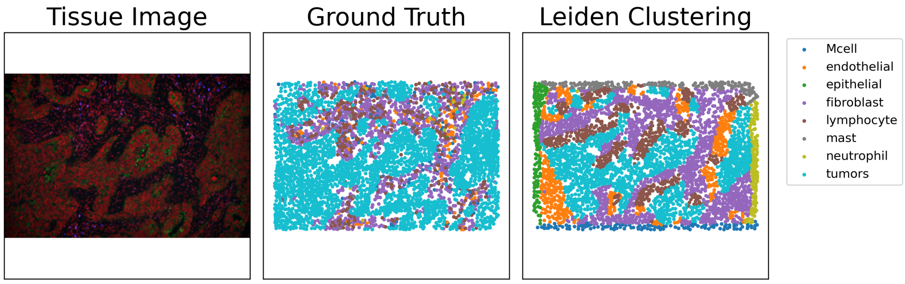

(c) Fov 014 of Nanostring

Fig. S6: (Left) Raw H&E tissue image. (Middle) Ground-truth annotations indicating known anatomical or cell-type regions. (Right) Spatial domains obtained by applying Leiden clustering *solely* to the visual embeddings extracted by S3RL, without using gene expression information. The visual-feature–based clusters recover coarse but biologically meaningful spatial patterns, including approximate cortical laminar organization and gray–white matter separation in DLPFC slices, as well as partial alignment with tumor- and stroma-enriched regions in the Nanostring lung cancer slice.

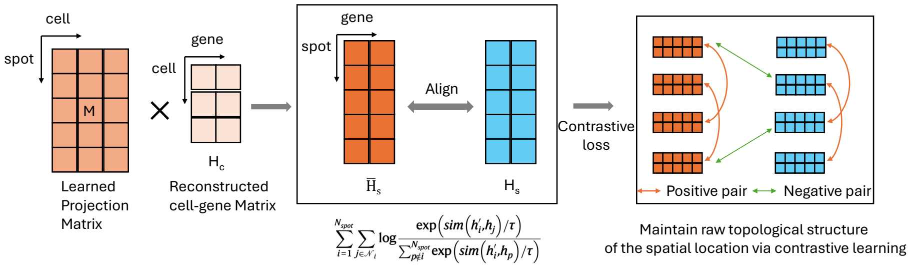

Fig. S7: Overview of the contrastive learning framework for spatial gene expression alignment. Instead of using an auto-encoder, the reconstructed cell-gene expression matrix H_c is directly utilized. A learned projection matrix M maps single-cell RNA-seq data to the spatial transcriptomics domain, generating the reconstructed spatial gene expression matrix $\overline{H}_s$. The alignment process ensures similarity between $\overline{H}_s$ and the true spatial gene expression H_s by optimizing a contrastive loss function. The loss maximizes similarities for positive pairs (spatially adjacent spots) and minimizes similarities for negative pairs (spatially non-adjacent spots), preserving the raw topological structure of spatial locations.

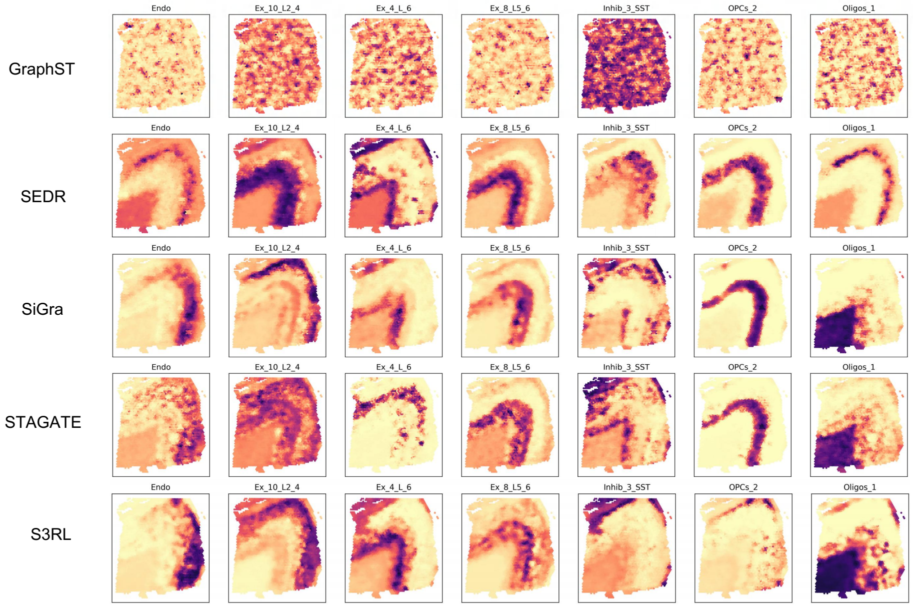

Fig. S8: Visualization of deconvolution results across methods on DLPFC slice 151673. Comparison of cell-type proportion maps generated by different reconstruction-capable methods (GraphST, SEDR, SiGra, STAGATE, and our proposed S3RL) under the same deconvolution framework. All models share the same contrastive deconvolution strategy as proposed in this work. Despite the lack of precise quantitative evaluation, visual inspection shows that S3RL yields the clearest spatial boundaries and the most coherent cell-type distributions, especially for major cell types such as Ex (excitatory neurons), Oligos (oligodendrocytes), and Endo (endothelial cells). These results further demonstrate the strong reconstruction quality and spatial consistency achieved by S3RL.

6 Latent Space Clustering Results on NanoString dataset

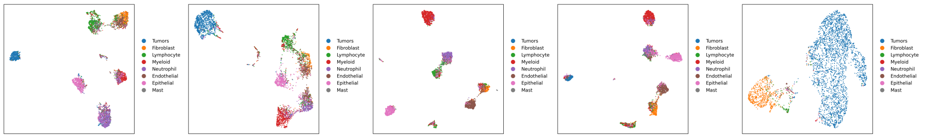

(a) FOV1 **(b)** FOV2 **(c)** FOV3 **(d)** FOV4 **(e)** FOV5

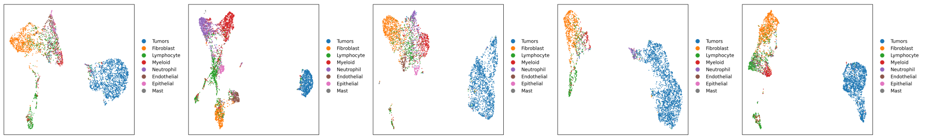

(f) FOV6 **(g)** FOV7 **(h)** FOV8 **(i)** FOV9 **(j)** FOV10

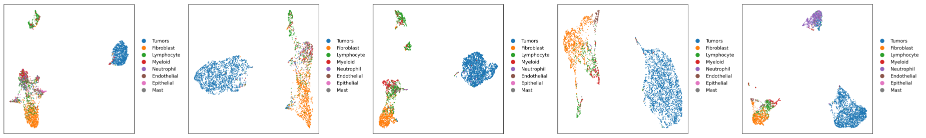

(k) FOV11 **(l)** FOV12 **(m)** FOV13 **(n)** FOV14 **(o)** FOV15

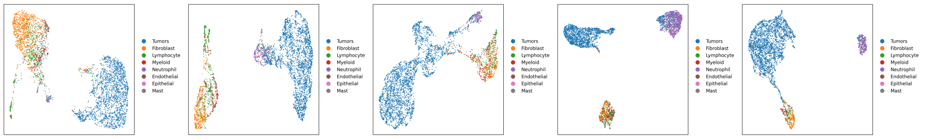

(p) FOV16 **(q)** FOV17 **(r)** FOV18 **(s)** FOV19 **(t)** FOV20

Fig. S9: Clustering results in the latent space for 20 lung cancer slices from the Nanostring dataset using S3RL. Each subplot represents the latent space embedding of one slice, where spots are colored according to their predicted clusters. The clustering results highlight the spatial organization of different cell populations.

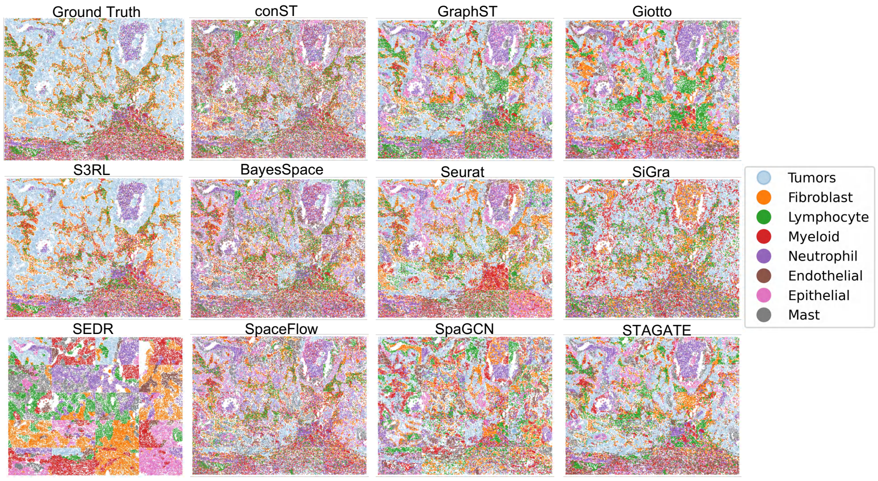

Fig. S10: Comparison of clustering results across 20 Nanostring lung cancer slices using different methods. The first image (top-left) represents the ground truth annotations, while the remaining images depict clustering results obtained from various spatial transcriptomics analysis frameworks, including conST, GraphST, Giotto, S3RL, BayesSpace, Seurat, SiGra, SpaceFlow, SpaGCN, and STAGATE. Our proposed S3RL method demonstrates improved spatial consistency and boundary precision in capturing cellular heterogeneity across lung tissue samples.

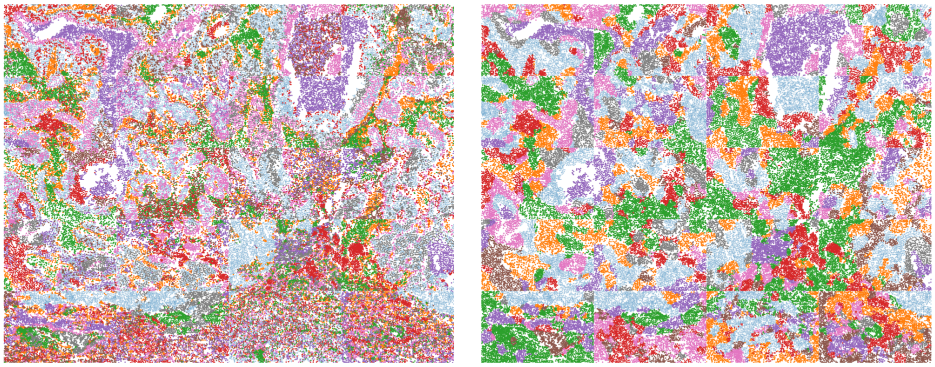

(a) stDCL **(b)** Banksy

Fig. S11: Clustering results of the stDCL and BankSY methods on the 20 slices of the Nanostring dataset.

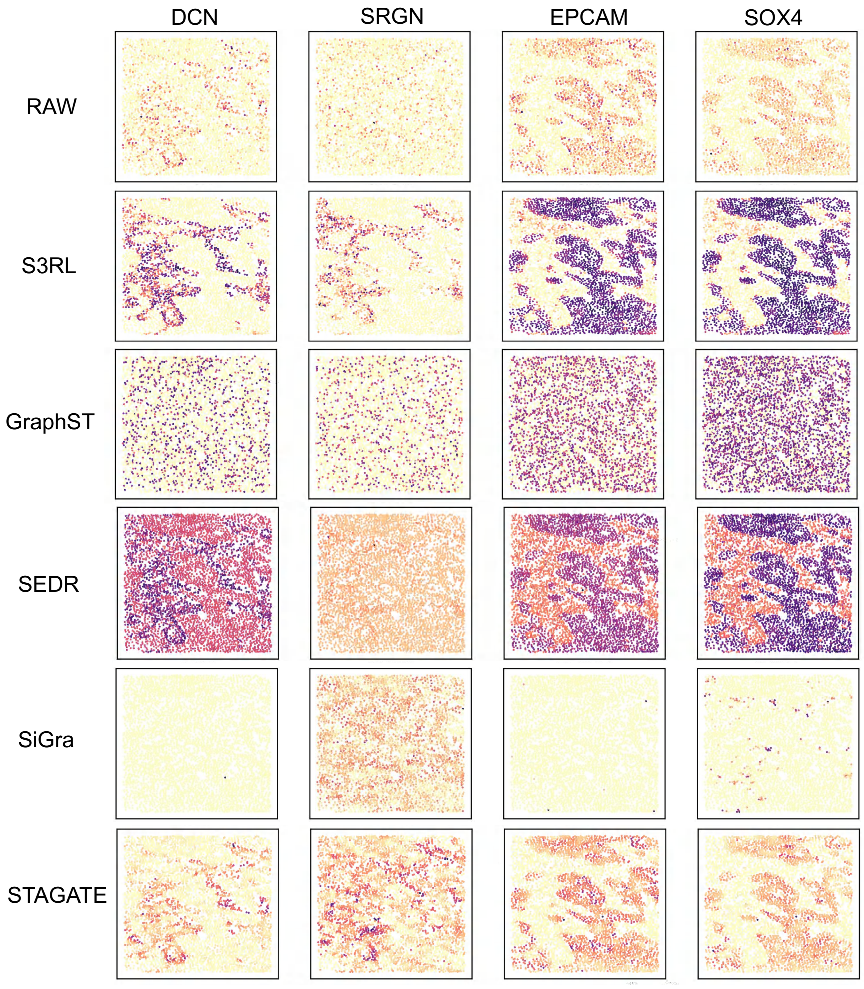

Fig. S12: Visualization of marker gene reconstruction performance across generative spatial transcriptomics models. Comparison of reconstructed spatial expression patterns for four key marker genes (*DCN*, *SRGN*, *EPCAM*, and *SOX4*) across different methods, including the raw ST data (top row), and the reconstructed outputs of S3RL, GraphST, SEDR, SiGra, and STAGATE. All methods were evaluated under the same reconstruction framework using their model-generated latent outputs. The S3RL reconstruction shows the clearest spatial boundaries and expression contrast, especially for *SRGN*, where it achieves a more distinguishable region separation compared to other methods. These results demonstrate the effectiveness of S3RL in capturing biologically relevant gene expression patterns during spatial reconstruction.

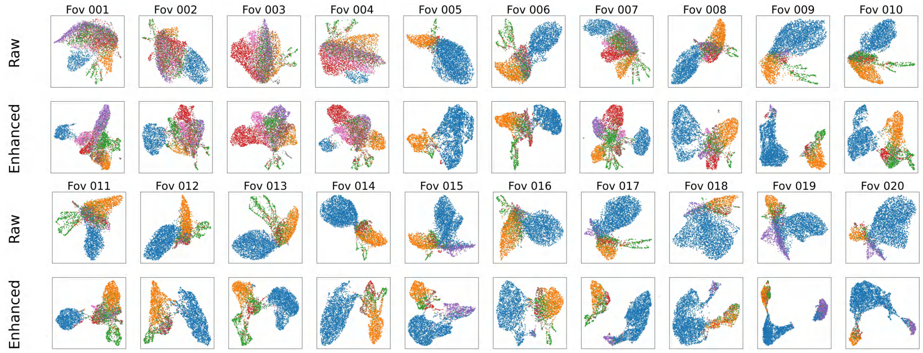

Fig. S13: Comparison of the latent space clustering performances in raw and enhanced clustering for Nanostring lung cancer slice data. The first and third rows show unprocessed data, while the second and fourth depict S3RL-enhanced embeddings. These enhanced embeddings illustrate cluster separability improved spatially and with cell-type distinction based on more appropriate clustering within the tumor microenvironment. Again, S3RL shows promise in spatial transcriptomics studies.

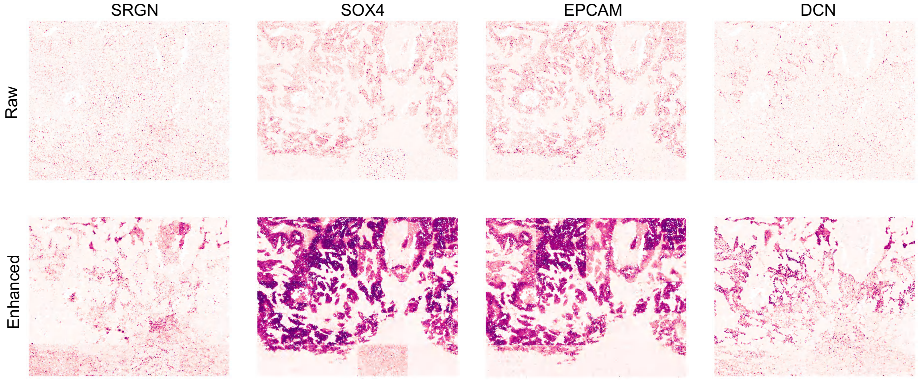

Fig. S14: Comparison of marker gene expression in raw and enhanced data using the S3RL in Nanostring lung cancer slice data. The top row is raw data, and the bottom row is enhanced data. The four selected markers (SRGN, SOX4, EPCAM, and DCN) are critical in lung cancer: SRGN is influenced by the tumor microenvironment modulation, SOX4 is a transcription factor that regulates epithelial-mesenchymal transition (EMT), EPCAM is an epithelial cancer marker associated with metastasis, and DCN (Decorin) is involved in ECM remodeling. The data enhancement clearly shows spatial expression patterns more consistent with expected tissue structure, underscoring S3RL's utility for resolution and biological interpretability.

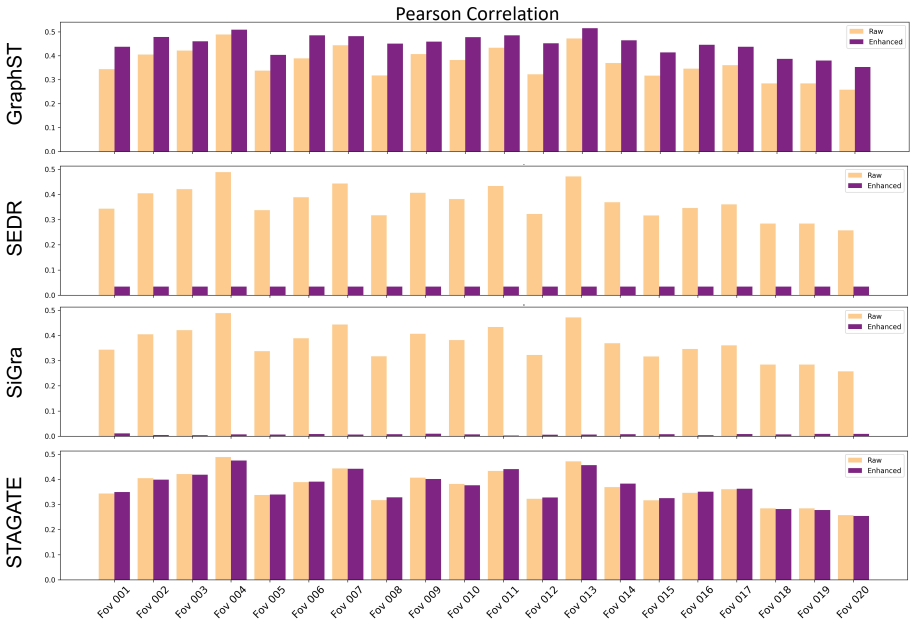

Fig. S15: Comparison of Pearson correlation between bulk RNA-seq (TCGA-LUAD) and raw/enhanced spatial transcriptomic data across 20 Nanostring lung slices of different methods. Bar plots showing the Pearson correlation between bulk RNA-seq (TCGA-LUAD) and spatial transcriptomic data (raw vs. enhanced) across 20 Nanostring lung slices for four reconstruction-capable methods: GraphST, SEDR, SiGra, and STAGATE.

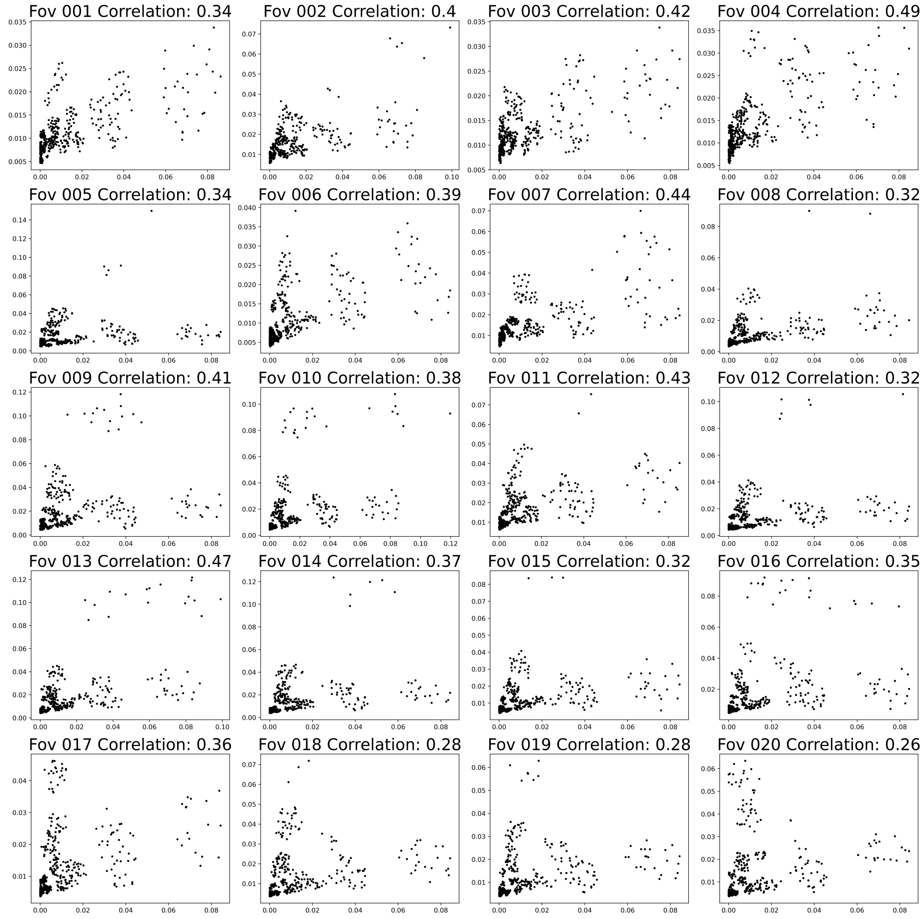

Fig. S16: Correlation analysis between bulk RNA-seq and raw spatial transcriptomics data in the Nanostring dataset. Each scatter plot corresponds to one tissue slice (FOV), where each point represents a gene. The x-axis and y-axis denote gene expression levels in the bulk RNA-seq and raw spatial transcriptomics data, respectively. The plots illustrate the correlation of expression patterns between bulk and spatial data under raw conditions, showing that S3RL enhancement improves consistency across most slices.

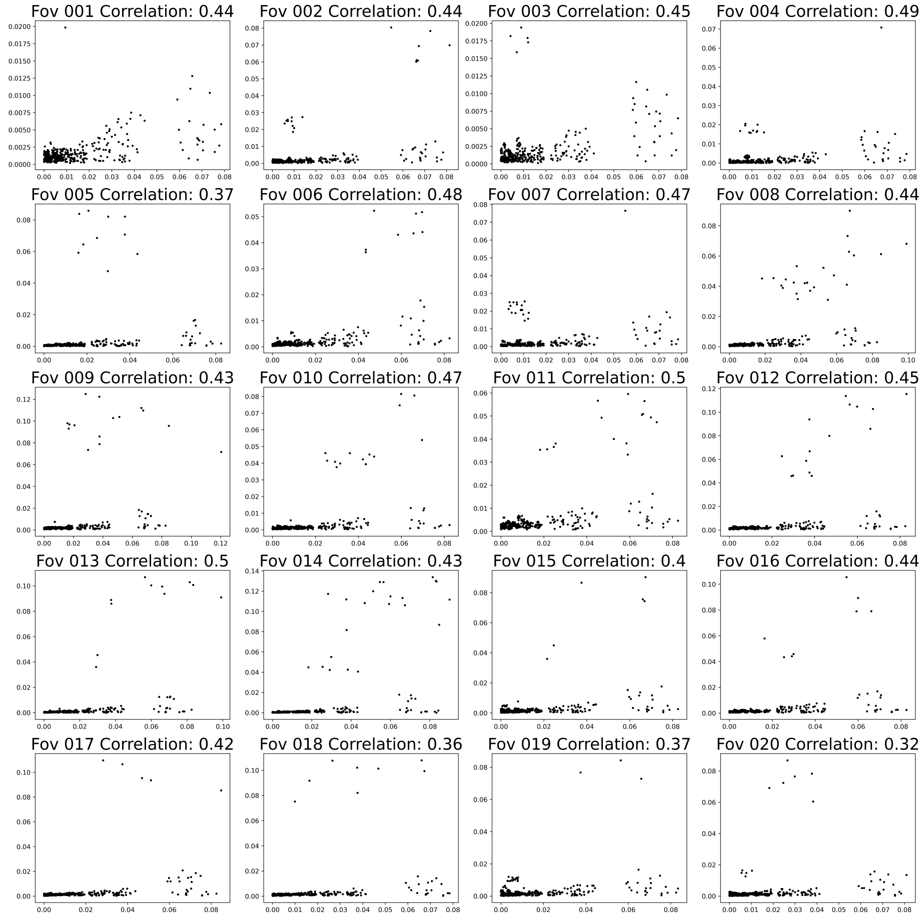

Fig. S17: Correlation analysis between bulk RNA-seq and enhanced spatial transcriptomics data in the Nanostring dataset. Each scatter plot corresponds to one tissue slice (FOV), where each point represents a gene. The x-axis and y-axis denote gene expression levels in the bulk RNA-seq and enhanced spatial transcriptomics data, respectively. The plots illustrate the correlation of expression patterns between bulk and spatial data under enhanced conditions, showing that S3RL enhancement improves consistency across most slices.

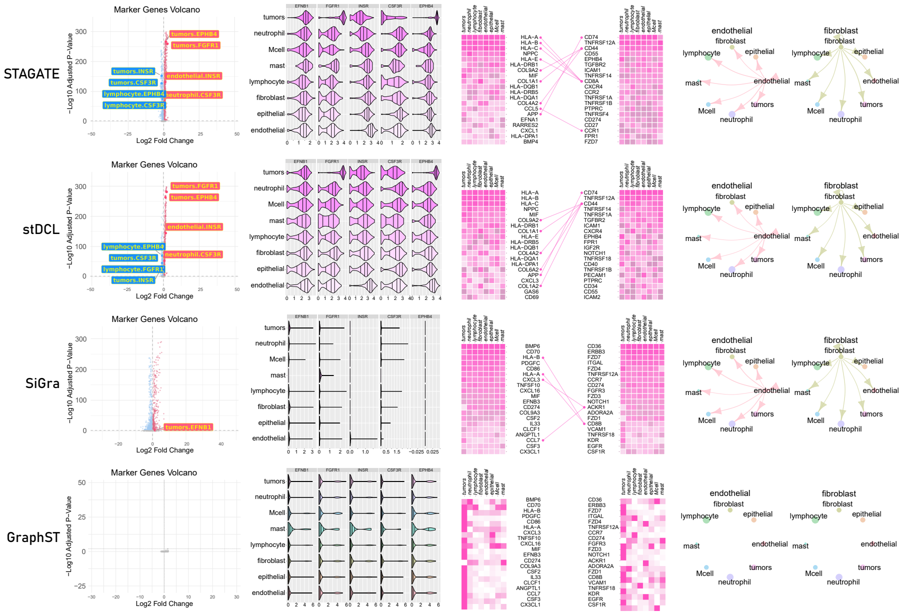

Fig. S18: Comparison of reconstruction-based methods on Nanostring lung cancer slice 001. Differential gene expression analysis, cell-type–specific expression profiles, and cell–cell communication patterns obtained using representative spatial reconstruction methods, including STAGATE, stDCL, SiGra, and GraphST. While several methods exhibit over-smoothed representations—characterized by highly similar violin plot distributions across distinct cell types and dense, non-specific cell–cell communication networks—the resulting patterns vary substantially in their ability to preserve biologically meaningful cell-type specificity and interaction structure, particularly within tumor and immune-related regions.

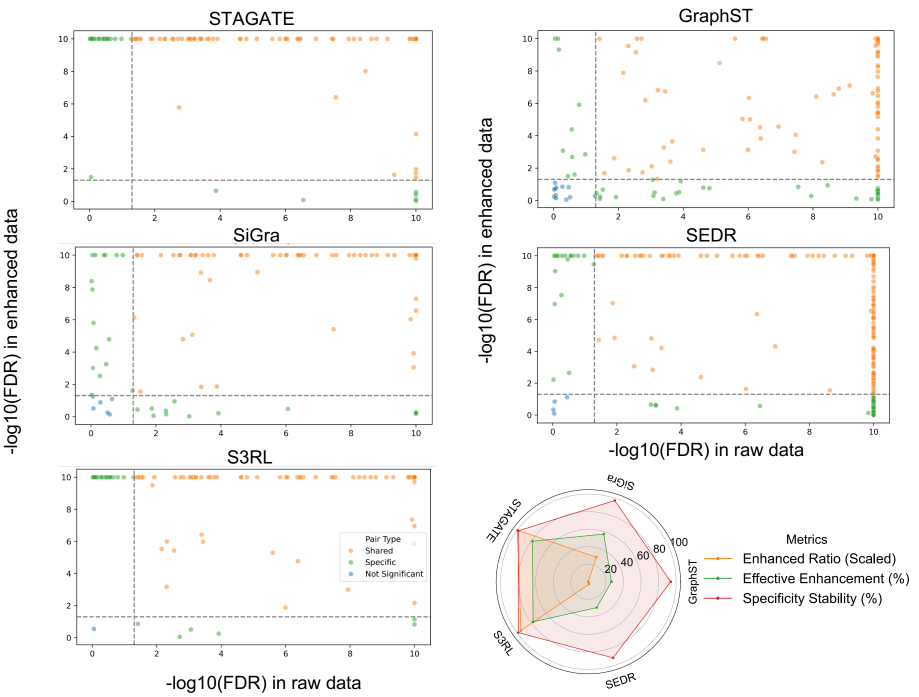

Fig. S19: Comparison of ligand-receptor (L-R) pair significance across different enhancement methods. Scatter plots show the significance level of L-R pairs based on $-\log_{10}(\text{FDR})$ in raw data (x-axis) and enhanced data (y-axis) for five methods: STAGATE, GraphST, SiGra, SEDR, and S3RL. Each dot represents an L-R pair, colored by its classification: shared (orange), enhanced-specific (green), or not significant (blue). The radar plot summarizes three evaluation metrics across methods: Enhanced Ratio (scaled), Effective Enhancement (%), and Specificity Stability (%).

7 Spatial Clustering Results on Mouse Brain Anterior and Human Breast Cancer Datasets

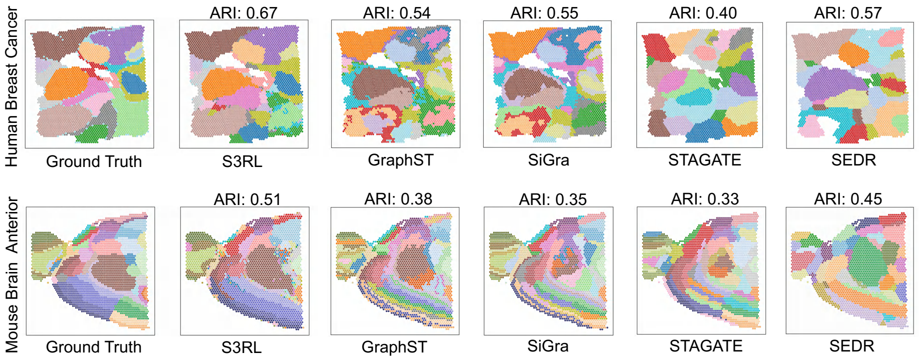

Fig. S20: Clustering comparison on Mouse Brain Anterior and Human Breast Cancer datasets. The spatial clustering results obtained by different methods (GraphST, SEDR, SiGra, STAGATE, and S3RL) on two representative datasets (10X Visium spatial transcriptomics datasets): Mouse Brain Anterior (top) and Human Breast Cancer (bottom). Ground truth annotations are provided as references. Adjusted Rand Index (ARI) values are reported above each result to quantify clustering accuracy. The results demonstrate that S3RL achieves higher ARI scores and better boundary alignment compared to other methods, providing more accurate spatial domain segmentation in both neural and tumor tissues.

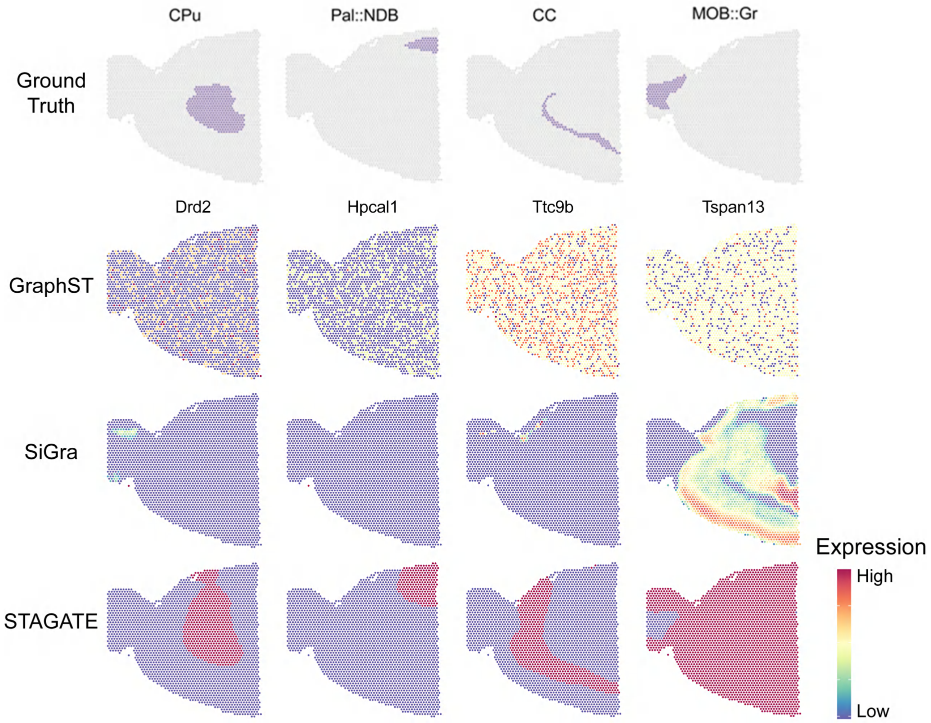

Fig. S21: Comparison of reconstructed marker gene expression patterns by baseline methods on the Mouse Brain Anterior dataset. The top row shows the ground truth cell-type annotations for each spatial region, while each subsequent row presents the spatial expression patterns of selected marker genes reconstructed by baseline models, including STAGATE, GraphST, and SEDR. Although certain patterns are partially recovered, the reconstructions often appear spatially diffused or lack boundary specificity, making it difficult to clearly delineate anatomical regions. This highlights the limitations of current generative methods in recovering fine-grained spatial gene expression in complex neural tissues.

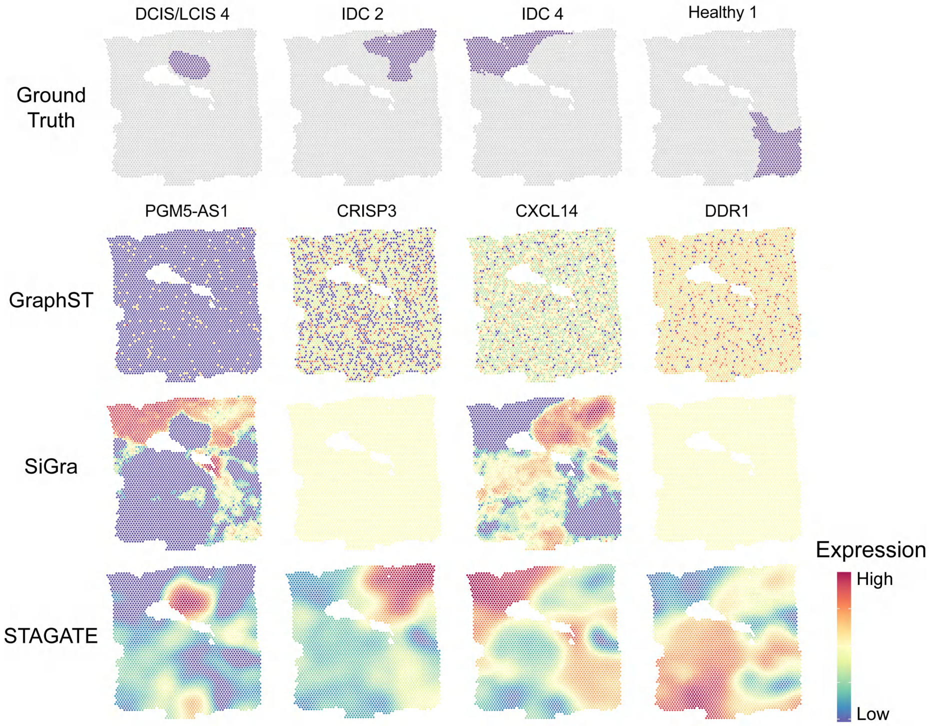

Fig. S22: Reconstruction of marker gene expression in human breast cancer tissue by different baseline methods. The top row displays ground truth annotations of spatial domains based on expert interpretation. The rows below show the reconstructed spatial expression patterns of key marker genes by different generative models. While some spatial structures are partially recovered, most methods exhibit diffuse or blurred boundaries, making it difficult to distinguish fine-grained tumor microenvironmental regions. This highlights the current limitations of generative frameworks in accurately capturing spatial heterogeneity in complex cancer tissues.

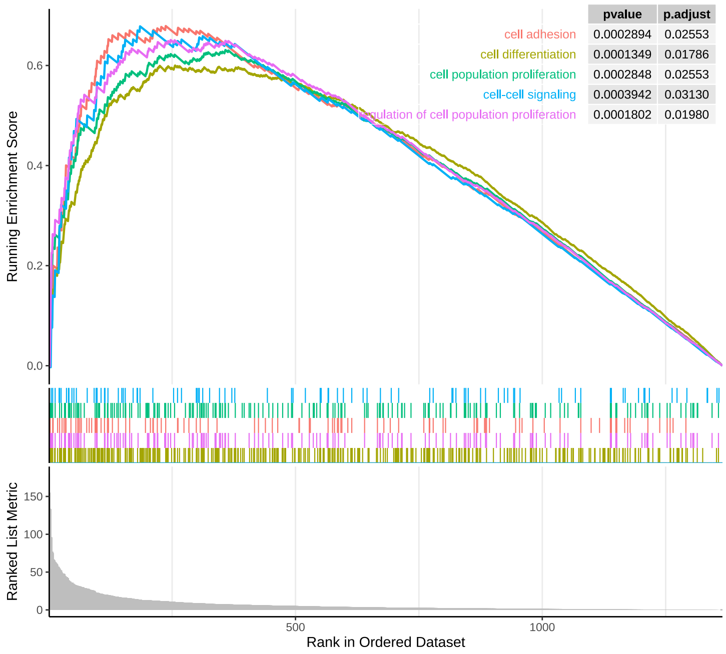

Fig. S23: GSEA enrichment plots for selected biological processes in human lung cancer spatial transcriptomics data. Enriched pathways include cell adhesion, cell differentiation, cell population proliferation, and cell-cell signaling. These processes are closely associated with tumor progression: abnormal cell adhesion facilitates metastasis, dysregulated differentiation contributes to tumor heterogeneity, and enhanced cell-cell signaling promotes immune interactions and angiogenesis.

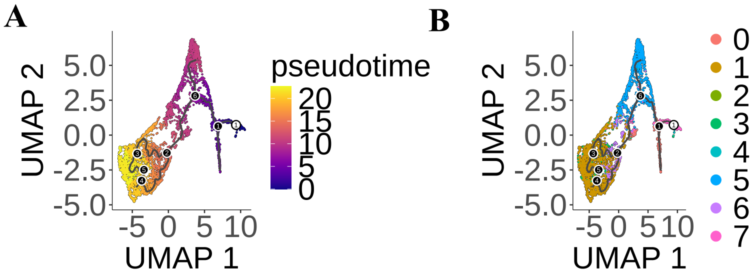

Fig. S24: Pseudotime trajectory inference in S3RL-enhanced latent space. (A) Visualization of the inferred pseudotime progression colored by pseudotime values, showing a continuous trajectory from early to late cellular states. (B) Cluster assignments identified in the latent space, illustrating the spatial distribution of cellular states across the tissue section. The S3RL-enhanced data enables clear delineation of pseudotemporal ordering and distinct cluster boundaries, supporting trajectory analysis in complex tumor microenvironments.

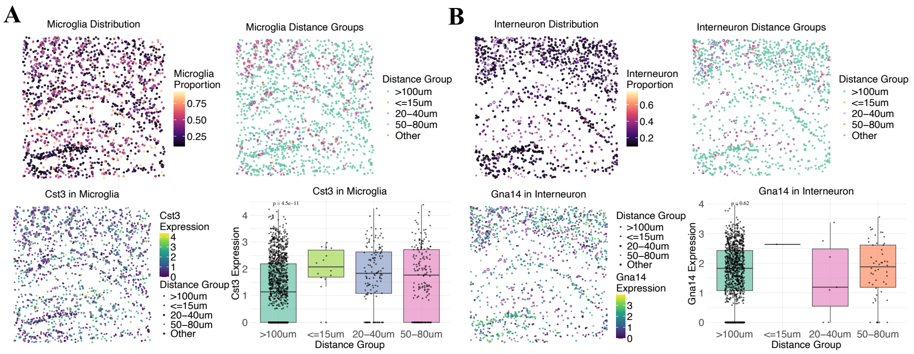

Fig. S25: Spatial distribution and gene expression patterns of microglia and interneurons in raw data. (A) Microglia distribution and Cst3 expression in mouse hippocampal plaque regions. The top row shows microglial proportions and distance-based groupings relative to plaques. The bottom row displays Cst3 spatial expression and boxplots comparing expression levels across distance groups. (B) Interneuron distribution and Gna14 expression in the same regions. The top row shows interneuron proportions and distance groupings, while the bottom row presents Gna14 spatial expression and boxplots of expression across distance groups. The raw data reveals limited spatial trends, with weak or non-significant differences in Gna14 expression relative to plaque proximity.

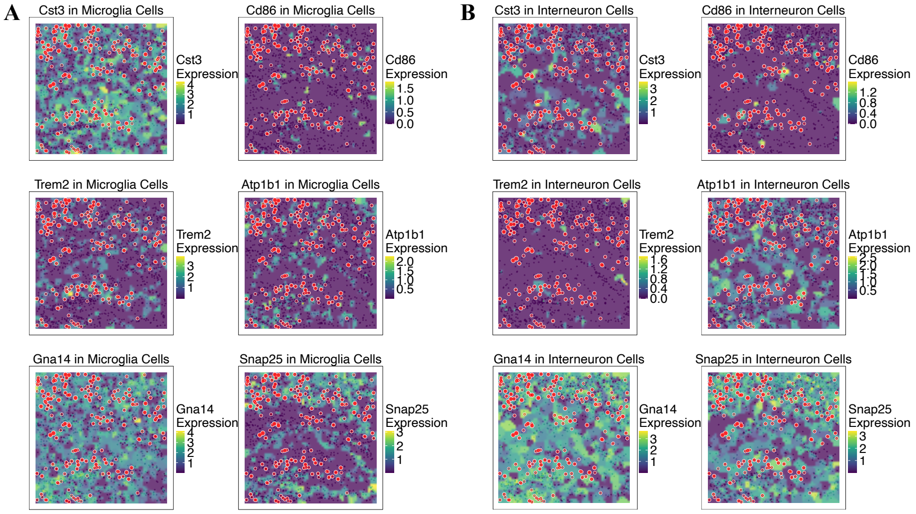

Fig. S26: Spatial expression of key genes in raw mouse hippocampal data. Heatmaps show the expression of Cst3, Cd86, Trem2, Atp1b1, Gna14, and Snap25 within microglia (A) and interneurons (B). Red points mark amyloid plaque locations. In the raw data, gene expression gradients appear diffuse and less distinct, making it challenging to discern fine-scale spatial patterns around plaques.

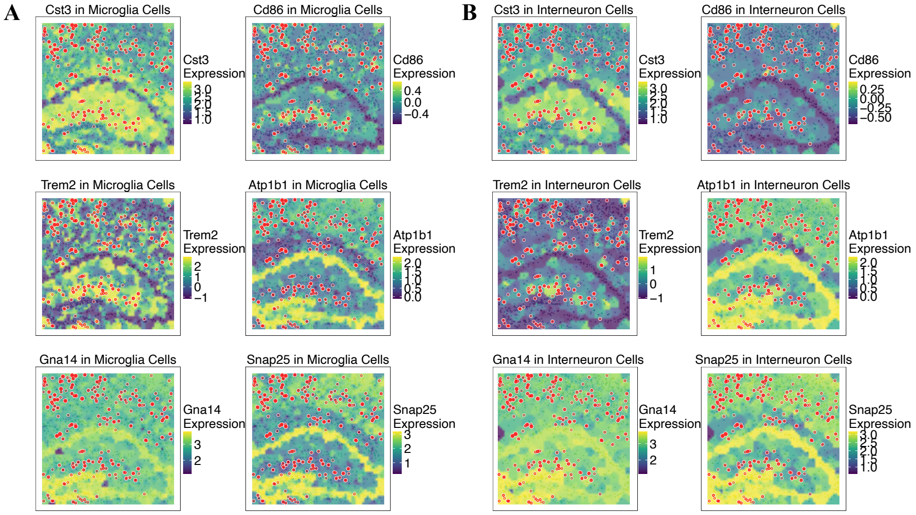

Fig. S27: Spatial expression of key genes in S3RL-reconstructed mouse hippocampal data. Heatmaps show enhanced expression patterns of Cst3, Cd86, Trem2, Atp1b1, Gna14, and Snap25 within microglia (A) and interneurons (B). Red points indicate amyloid plaque positions. Compared to raw data, S3RL reconstruction reveals clearer spatial gradients and sharper gene expression boundaries around plaques.

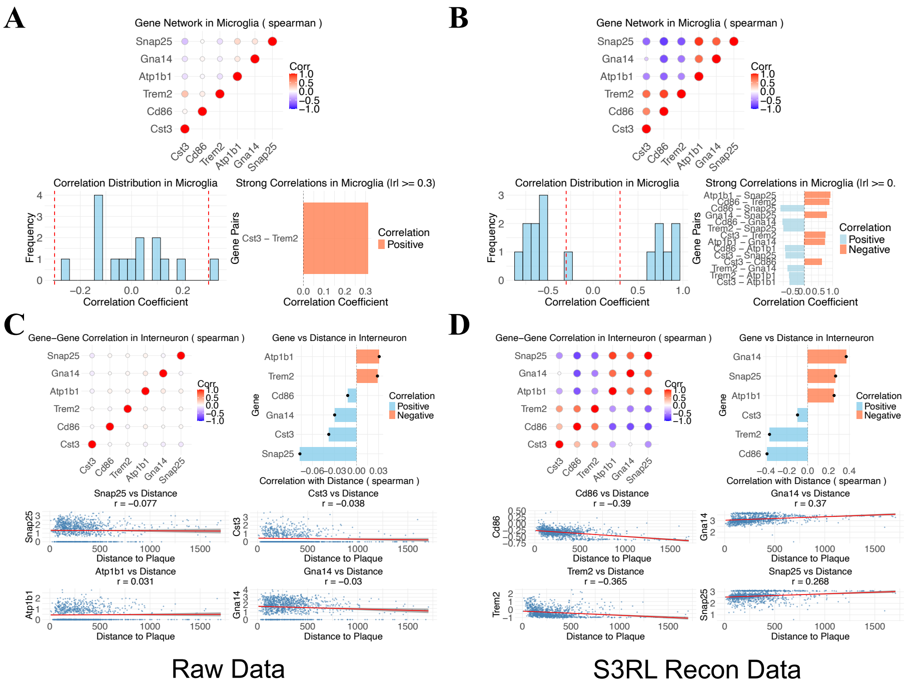

Fig. S28: Gene co-expression networks and spatial correlations in microglia and interneurons between raw and S3RL-reconstructed data. (A, B) Gene–gene co-expression networks and correlation distributions in microglia. Raw data (A) exhibits minimal detectable correlations, with most gene pairs showing weak or no co-expression and only a single pair (Cst3–Trem2) exceeding the correlation threshold. In contrast, S3RL-reconstructed data (B) reveals multiple strong positive and negative correlations, forming clear co-expression modules among inflammatory markers. (C, D) Gene–gene co-expression networks and correlations in interneurons. Raw data (C) shows low correlation coefficients and weak spatial trends, while S3RL-reconstructed data (D) presents stronger correlations and clearer associations with plaque proximity (e.g., Cd86 and Gna14). Scatter plots (bottom) visualize gene expression changes relative to plaque distance for selected genes in both datasets.

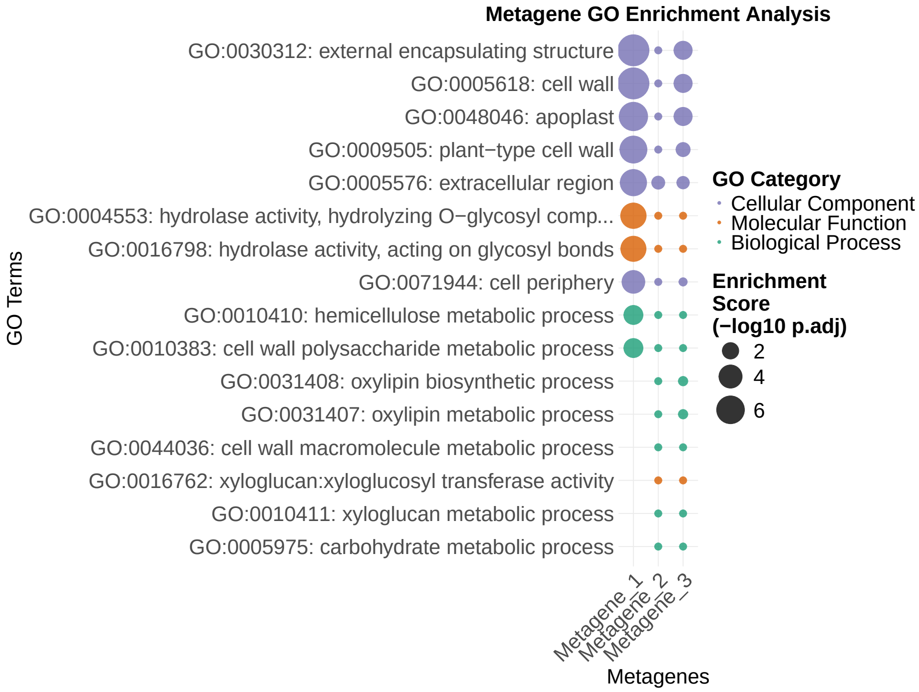

Fig. S29: Gene Ontology (GO) enrichment analysis of metagenes in soybean vascular parenchyma cells. The heatmap displays GO terms enriched in three metagenes identified from S3RL-reconstructed data using CELINA. GO categories include Biological Process, Molecular Function, and Cellular Component, with enrichment scores represented as $-\log_{10}$(adjusted p-values). Metagene 1 shows strong enrichment in cell wall-related processes such as hemicellulose metabolic process, xyloglucan metabolic process, and cell wall polysaccharide metabolic process, aligning with the structural and transport roles of vascular parenchyma cells. Metagene 2 and Metagene 3 are enriched in additional processes including oxylipin biosynthetic process and localization to the cell periphery, reflecting functional diversity within vascular tissues.

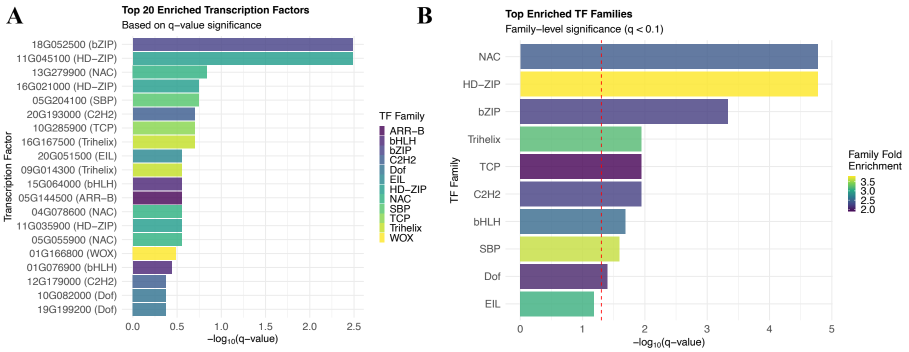

Fig. S30: Transcription factor enrichment analysis based on S3RL-reconstructed data. (A) The top 20 enriched transcription factors (TFs) identified in SC vascular parenchyma cells using the PlantRegMap platform, visualized as a bar plot ranked by $-\log_{10}(q\text{-value})$. Significant TFs include NAC, HD-ZIP, bZIP, Trihelix, and C2H2 families. (B) Family-level TF enrichment analysis showing fold enrichment and significance levels ($q < 0.1$) for major TF families. Families such as HD-ZIP, NAC, and bZIP are highly enriched, consistent with their roles in vascular development and secondary cell wall formation.

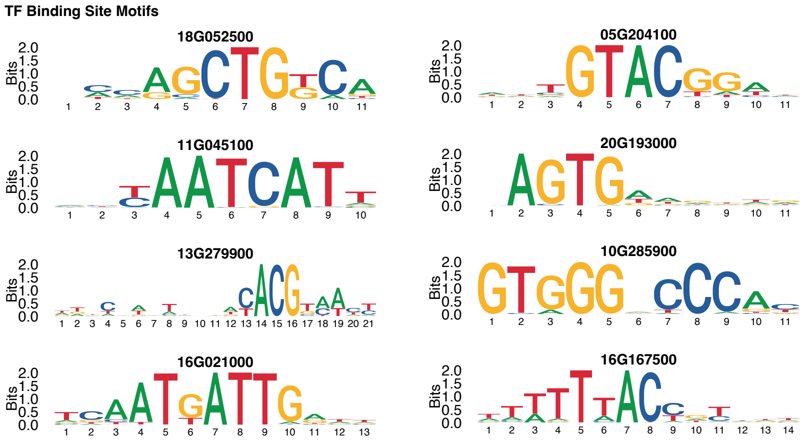

Fig. S31: Predicted transcription factor (TF) binding site motifs for soybean SC vascular parenchyma regulatory network. TF binding motifs identified for the top 8 enriched transcription factors (TFs) in SC vascular parenchyma cells, including 18G052500 (bZIP), 11G045100 (HD-ZIP), 13G279900 (NAC), 16G021000 (HD-ZIP), 05G204100 (SBP), 20G193000 (C2H2), 10G285900 (TCP), and 16G167500 (Trihelix). The motif logos were derived using FIMO analysis from the MEME suite.

8 Detailed Ablation Study and Module Contributions

To rigorously dissect the mechanistic contribution of each component within the S3RL framework, we conducted a comprehensive ablation study. We evaluated three specific variants to isolate the effects of visual semantic guidance and dynamic prototype learning.

8.1 Definition of Ablation Variants

- **S3RL-1 (Fixed Prototypes):** This variant retains the semantic information extraction module (constructing signed edges from visual similarity) but utilizes static prototypes. It isolates the effect of prototype dynamics while keeping spatial semantic guidance intact.
- **S3RL-2 (No Visual Semantics):** This variant removes the visual-feature-based semantic graph while retaining dynamic prototypes. It allows us to evaluate the importance of visual semantic guidance for spatial smoothness.
- **S3RL-3 (Baseline):** The most basic configuration, relying solely on location-based adjacency and static prototypes. This serves as the baseline for assessing the incremental contribution of each module.

8.2 Visual Semantics as a Spatial Denoiser

The qualitative impact of visual semantics is evident in the spatial domain reconstruction. As illustrated in Fig. S32, variants lacking visual semantics (S3RL-2 and S3RL-3) exhibit noticeable "salt-and-pepper" noise and discontinuous tissue boundaries. For instance, in DLPFC Slice 151510, S3RL-2 fails to maintain laminar continuity due to expression dropouts. In contrast, by incorporating visual semantics through signed-edge construction, S3RL and S3RL-1 enforce spatial coherence. The visual features effectively act as a spatial regularizer, correcting isolated misclassified spots and yielding smooth, biologically consistent cortical layers.

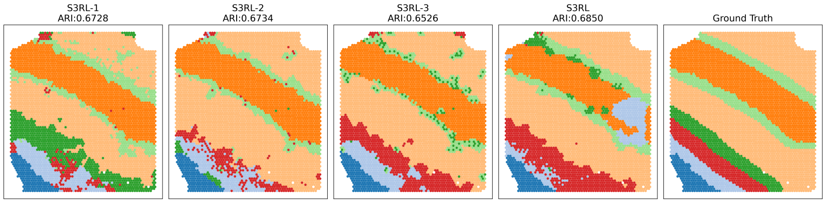

(a) Slice 151509: Spatial Clustering

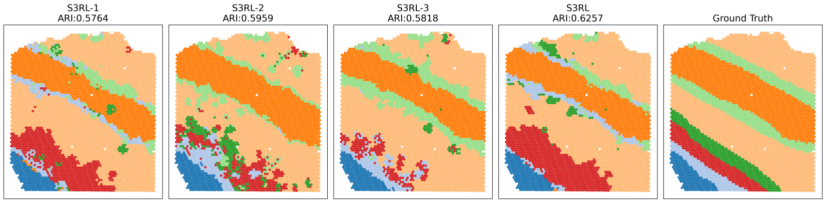

(b) Slice 151510: Spatial Clustering

Fig. S32: Models without visual semantic guidance (S3RL-2, S3RL-3) produce fragmented domains, whereas the full S3RL model yields spatially coherent tissue structures.

8.3 Dynamic Prototype Learning Enhances Latent Separability

We further analyzed the latent space structure using UMAP visualizations (Fig. S33). Variants with fixed prototypes (S3RL-1 and S3RL-3) tend to produce scattered clusters with blurred inter-class boundaries. This suggests that static prototypes fail to adapt to the heterogeneous expression distributions inherent in complex tissues. Conversely, the dynamic update strategy employed in S3RL and S3RL-2 successfully pulls intra-class samples together while pushing inter-class samples apart. This mechanism results in highly compact and well-separated clusters, which directly correlates with the improved ARI scores observed in the quantitative benchmarks.

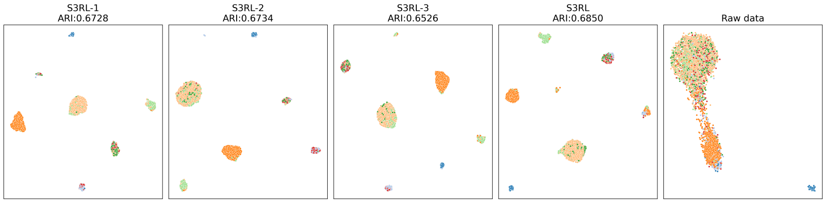

(a) Slice 151509: Latent Embeddings

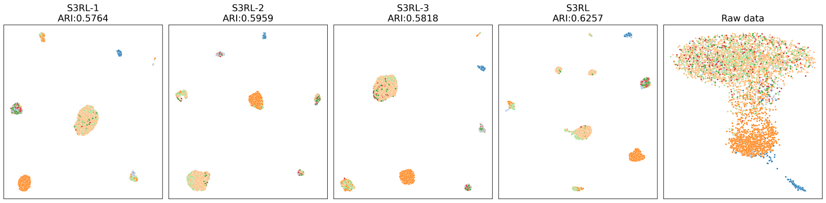

(b) Slice 151510: Latent Embeddings

Fig. S33: Dynamic prototype learning (present in S3RL and S3RL-2) significantly improves cluster compactness and separability compared to static approaches.

Table S1: Comparison of clustering performance measured by ARI (mean ± std) across different methods on the DLPFC dataset (12 slices) and the Nanostring dataset (20 slices).

**Method**	**DLPFC**	**Nanostring**
BayesSpace	0.4615±0.1090	0.2919±0.0735
Giotto	0.3464±0.1021	0.2017±0.0554
Seurat	0.2880±0.0741	0.3989±0.1179
SiGra	0.5492±0.0757	0.2748±0.1386
conST	0.3540±0.0937	0.1999±0.1177
SpaceFlow	0.2172±0.0617	0.3025±0.1004
SpaGCN	0.3880±0.0837	0.2700±0.0847
STAGATE	0.4119±0.0947	0.2716±0.1075
GraphST	0.5200±0.0803	0.2887±0.0955
stDCL	0.4185±0.0866	0.1313±0.0552
Banksy	0.4653±0.1114	0.2948±0.0985
SEDR	0.5283±0.1172	0.2959±0.1460
S3RL	0.6508±0.0700	0.7274±0.1512

9 Supplementary References

References

1. Suoqin Jin, Christian F Guerrero-Juarez, Lihua Zhang, Ivan Chang, Raul Ramos, Chen-Hsiang Kuan, Peggy Myung, Maksim V Plikus, and Qing Nie. Inference and analysis of cell-cell communication using cellchat. *Nature communications*, 12(1):1088, 2021.
2. Yangli Xie, Nan Su, Jing Yang, Qiaoyan Tan, Shuo Huang, Min Jin, Zhenhong Ni, Bin Zhang, Dali Zhang, Fengtao Luo, et al. Fgf/fgfr signaling in health and disease. *Signal transduction and targeted therapy*, 5(1):181, 2020.
3. Pei Zhang, Lin Yue, QingQing Leng, Chen Chang, Cailing Gan, Tinghong Ye, and Dan Cao. Targeting fgfr for cancer therapy. *Journal of Hematology & Oncology*, 17(1):39, 2024.
4. Venkatram Yellapragada, Nazli Eskici, Yafei Wang, Shrinidhi Madhusudan, Kirsi Vaaralahti, Timo Tuuri, and Taneli Raivio. Fgf8–fgfr1 signaling regulates human gnrh neuron differentiation in a time-and dose-dependent manner. *Disease Models & Mechanisms*, 15(8):dmm049436, 2022.
5. Xingrao Ke, Sheng Xia, Wei Yu, Sherry Mabry, Qi Fu, Heather L Menden, Venkatesh Sampath, and Robert H Lane. Delta like 4 regulates cerebrovascular development and endothelial integrity via dll4-notch-cldn5 pathway and is vulnerable to neonatal hyperoxia. *The Journal of physiology*, 602(10):2265–2285, 2024.
6. Jin-feng Zhang, Yao Chen, Xian-xin Qiu, Wen-long Tang, Jian-dong Zhang, Jian-huang Huang, Guo-shi Lin, Xing-fu Wang, and Zhi-xiong Lin. The vascular delta-like ligand-4 (dll4)-notch4 signaling correlates with angiogenesis in primary glioblastoma: an immunohistochemical study. *Tumor Biology*, 37:3797–3805, 2016.
7. Patrycja Nowak-Sliwinska, Judy R van Beijnum, Elisabeth JM Huijbers, Paula C Gasull, Laurie Mans, Axel Bex, and Arjan W Griffioen. Oncofoetal insulin receptor isoform a marks the tumour endothelium; an underestimated pathway during tumour angiogenesis and angiostatic treatment. *British journal of cancer*, 120(2):218–228, 2019.
8. Harriet R Ferguson, Michael P Smith, and Chiara Francavilla. Fibroblast growth factor receptors (fgfrs) and noncanonical partners in cancer signaling. *Cells*, 10(5):1201, 2021.
9. Melanie A Krook, Julie W Reeser, Gabrielle Ernst, Hannah Barker, Max Wilberding, Gary Li, Hui-Zi Chen, and Sameek Roychowdhury. Fibroblast growth factor receptors in cancer: genetic alterations, diagnostics, therapeutic targets and mechanisms of resistance. *British Journal of Cancer*, 124(5):880–892, 2021.
10. Elena B Pasquale. Eph receptors and ephrins in cancer progression. *Nature Reviews Cancer*, 24(1):5–27, 2024.
11. Glinton Hanover, Frederick S Vizeacoumar, Sara L Banerjee, Raveena Nair, Renuka Dahiya, Ana I Osornio-Hernandez, Alain Morejon Morales, Tanya Freywald, Juha P Himanen, Behzad M Toosi, et al. Integration of cancer-related genetic landscape of eph receptors and ephrins with proteomics identifies a crosstalk between ephb6 and egfr. *Cell reports*, 42(7), 2023.
12. Benjamin D Ferguson, Ren Liu, Cleo E Rolle, Yi-Hung Carol Tan, Valery Krasnoperov, Rajani Kanteti, Maria S Tretiakova, Gustavo M Cervantes, Rifat Hasina, Robyn D Hseu, et al. The ephb4 receptor tyrosine kinase promotes lung cancer growth: a potential novel therapeutic target. *PloS one*, 8(7):e67668, 2013.
